# Demography, dynamics and data: building confidence for simulating changes in the world's forests

**DOI:** 10.1111/nph.70643

**Published:** 2025-10-23

**Authors:** Annemarie H. Eckes‐Shephard, Arthur P. K. Argles, Bogdan Brzeziecki, Peter M. Cox, Martin G. De Kauwe, Adriane Esquivel‐Muelbert, Rosie A. Fisher, George C. Hurtt, Jürgen Knauer, Charles D. Koven, Aleksi Lehtonen, Sebastiaan Luyssaert, Laura Marqués, Lei Ma, Guillaume Marie, Jonathan R. Moore, Jessica F. Needham, Stefan Olin, Mikko Peltoniemi, Karl Piltz, Hisashi Sato, Stephen Sitch, Benjamin D. Stocker, Ensheng Weng, Daniel Zuleta, Thomas A. M. Pugh

**Affiliations:** ^1^ Department of Physical Geography and Ecosystem Science Lund University Lund 22362 Sweden; ^2^ Met Office Hadley Centre Exeter Devon EX1 PB UK; ^3^ Department of Silviculture, Institute of Forest Sciences Warsaw University of Life Sciences Warsaw 02‐776 Poland; ^4^ Department of Mathematics and Statistics, Faculty of Environment, Science and Economy University of Exeter Exeter EX4 4QF UK; ^5^ School of Biological Sciences University of Bristol Bristol BS8 1TQ UK; ^6^ School of Geography, Earth and Environmental Sciences University of Birmingham Birmingham B15 2TT UK; ^7^ Birmingham Institute of Forest Research University of Birmingham Birmingham B15 2TT UK; ^8^ CICERO Center for International Climate Research Oslo 0349 Norway; ^9^ Department of Geographical Sciences University of Maryland College Park MD 20742 USA; ^10^ Hawkesbury Institute for the Environment Western Sydney University Penrith NSW 2751 Australia; ^11^ School of Life Sciences, Faculty of Science University of Technology Sydney Ultimo NSW 2007 Australia; ^12^ Climate and Ecosystem Sciences Division Lawrence Berkeley National Laboratory Berkeley CA 94720 USA; ^13^ Natural Resources Institute Finland (Luke) Helsinki FI‐00790 Finland; ^14^ Amsterdam Institute for Life and Environment Vrije Universiteit Amsterdam Amsterdam 1081 the Netherlands; ^15^ Institute of Geography University of Bern Bern 3012 Switzerland; ^16^ Oeschger Centre for Climate Change Research University of Bern Bern 3012 Switzerland; ^17^ Science Partners Paris 75010 France; ^18^ Research Institute for Global Change (RIGC), Japan Agency for Marine‐Earth Science and Technology (JAMSTEC) 3173‐25 Showamachi, Kanazawa‐ku Yokohama 236‐0001 Japan; ^19^ Department of Geography, Faculty of Environment, Science and Economy University of Exeter Exeter EX4 4QF UK; ^20^ Center for Climate Systems Research, Columbia University and NASA Goddard Institute for Space Studies New York NY 10025 USA; ^21^ Forest Global Earth Observatory Smithsonian Tropical Research Institute Washington DC 20013‐7012 USA; ^22^ Department of Biological and Environmental Sciences University of Gothenburg Gothenburg 413 90 Sweden

**Keywords:** demographic vegetation model benchmarking, forest demography, growth–mortality dynamics, land‐surface modelling, model intercomparison, postdisturbance recovery, self‐thinning, vegetation carbon

## Abstract

- Vegetation demographic models (VDMs) are advanced tools for simulating forest responses to climate and land‐use changes, and are essential for projecting carbon cycling and large‐scale forest management strategies. Despite their increasing incorporation into Earth System Models, VDMs differ in their demographic assumptions, with no prior quantitative comparison of their performance.
- We benchmarked nine VDMs against observational data from boreal, temperate and tropical sites, assessing their accuracy in predicting tree growth, carbon turnover, biomass stocks and size distributions. Models were simulated under consistent climate conditions with postdisturbance recovery monitored for at least 420 yr.
- Postdisturbance carbon recovery trajectories showed significant variability while remaining within observational ranges. Initial regrowth rates varied substantially (0.03–0.60, 0.18–0.70 and 0.35–1.10 kgCm^−2^ yr^−1^ for boreal, temperate and tropical sites, respectively), influenced by each model's initial forest state. Models captured mature forest carbon content but showed compensating effects between overestimated growth and underestimated mortality rates.
- This first multi‐model benchmarking identifies growth and mortality rates as critical calibration targets and highlights the need to refine postdisturbance establishment conditions for model development. We outline specific benchmarking variables needed to improve predictions of forest responses to environmental change.

## Introduction

Forests play a key role in the climate system, via storage of carbon, interactions with the water cycle, and provision of numerous other ecosystem services. Large‐scale assessments of future forest dynamics, state and distribution in response to climatic and land‐use changes, are commonly simulated using Dynamic Global Vegetation Models (DGVMs; Cramer *et al*., 2001; Sitch *et al*., 2008; Friend *et al*., 2014). The first generation of such models that emerged in the late 1990s and early 2000s is still widely applied, but is limited by their omission of forest structural heterogeneity and demographic process representation (Friend *et al*., 2014). Recent studies indicate that a substantial portion of global forests is currently recovering from historical land‐use change and disturbances (Shevliakova *et al*., 2009; Hurtt *et al*., 2011, 2020; Pugh *et al*., 2019, 2024; Besnard *et al*., 2021; Dalmonech *et al*., 2022), a phenomenon that cannot be captured without model representation of forest size structure. These recovering forests display varying strength of carbon uptake and storage over their regrowth period (Pretzsch *et al*., 2014). They further influence the extent to which forests reflect incoming radiation and exchange water vapour with the atmosphere (Bonan, 2008). Meanwhile, ongoing environmental change influences individual‐tree physiology and hence demographic rates and turnover times. For example, environmental conditions affect tree growth and mortality rates (cf. Pretzsch *et al*., 2023), both at the level of the individual (Bugmann & Bigler, 2011; Needham *et al*., 2020) and manifesting at the stand level (Yu *et al*., 2024). These are dynamics that can most realistically be captured by models representing the interplay of tree growth and mortality rates with forest structure. Therefore, realistically simulating current and future vegetation demographics is necessary for forecasting both carbon cycling and local water and energy dynamics. Furthermore, anticipating the impacts of forest management strategies as a means to reach net zero commitments requires a representation of forest demography.

The first generation of DGVMs largely approximates vegetation heterogeneity through distinguishing plant functional types (PFTs), while each PFT is represented by a population of geometrically and functionally identical individuals. This approach simplifies the behaviour of a population to that of an ‘average individual’, meaning that a change in environmental conditions triggers a single PFT‐specific average physiological response. This response manifests on all associated carbon pools (e.g. leaves, stems and roots) in the same proportional way, rather than allowing for variability among individuals. The size of the individual depends on productivity and mortality rates of the PFT in a given gridcell and PFT‐specific allometric relationships. Even in models where the vegetation distribution is dynamic, these vegetation dynamics are limited to simplified competition between PFTs, based on the resource use efficiency and productivity of a given PFT's average‐individual response to climatic conditions. Well‐performing individuals would ultimately inhabit a larger fraction of a gridcell. Notably, demography is highly simplified. Mortality is considered by reducing the number of (even‐sized) individuals. Recruitment is considered by increasing the number of trees but (artificially) mixing the new saplings' mass with existing stock, thus reducing the size of the average individual to conserve carbon (C) mass. This necessary artefact precludes any meaningful comparison with the large number of individual‐tree‐level observations that exist (e.g. tree rings or dendrometer data, or repeated forest inventories tracking individuals), foregoing a powerful potential constraint on the modelled behaviour. Overall, while computationally efficient, the average‐individual approach obscures the impact of demographic processes, with substantial implications for the accurate simulation of vegetation recovery and responses to environmental change.

In reality, demographic processes are fundamental to forest compositional and structural dynamics. For example, growth and mortality processes of individual trees change the self‐organisation of tree crowns and gap dynamics, thus affecting the light profile within the forest and driving successional changes in forest structure and composition (Brown & Parker, 1994; Canham *et al*., 1994; Montgomery & Chazdon, 2001; Matsuo *et al*., 2021). In turn, the light profile and other micro‐environmental conditions caused by forest structure influence establishment rates (Gleason, 1917; Klopcic & Boncina, 2012). More specifically, tree growth and establishment rates are driven by species‐specific strategies (Wright *et al*., 2010; Reich, 2014), modified by climatic conditions (Lempereur *et al*., 2015; Fridley & Wright, 2018; Babst *et al*., 2019) and forest light conditions (Klopcic & Boncina, 2012). For example, both within regrowing natural (Chave *et al*., 2020; Pretzsch *et al*., 2023) and plantation forests (West *et al*., 1981), growth rates can vary driven by competitive tree–tree interactions alone. As competition for resources (e.g. light, soil water and nutrients) intensifies with plant growth, this can lead to mortality. In even‐aged monospecific forests, this competition‐based mortality process has been described as the ‘self‐thinning law’ (Reineke, 1933; Yoda *et al*., 1963; Enquist *et al*., 1998), and is used extensively in plantation forest management (Reineke, 1933; West *et al*., 1997). Other mechanisms of mortality are dependent on tree size (Metcalf *et al*., 2009; Johnson *et al*., 2018), as many physiological processes are size‐dependent. Likewise, tree size is a critical agent for light competition: tall trees can overtop the shorter trees and gain advantage. Size is a major determinant of the resilience and vulnerability of trees to drought (Nepstad *et al*., 2007; da Costa *et al*., 2010; Bennett *et al*., 2015; McDowell & Allen, 2015), wind damage (Yap *et al*., 2016), fire (Pausas & Keeley, 2021) and insect outbreaks (Carnicer *et al*., 2011; Oliva *et al*., 2014). The interplay of these demographic processes in a forest collectively determines the structure, resilience and vegetation response to environmental changes.

Overall, the change in tree growth and mortality rates throughout the course of forest regrowth changes the trajectory of carbon uptake and storage through time (Pretzsch *et al*., 2014). Likewise, albedo and exchange of water with the atmosphere also vary in response to successional dynamics (Bonan, 2008), influencing carbon and local water and energy dynamics. Demographic processes influence and are influenced by forest functional diversity (Muscarella *et al*., 2017; Ruiz‐Benito *et al*., 2017) and impact mammal habitat structure (Grelle, 2003; Sukma *et al*., 2019). Therefore, forest demography ultimately impacts many aspects of ecosystem biogeochemical cycles, making the realistic modelling of these processes crucial (Bonan, 2008; Levine *et al*., 2016) even for LSMs.

Vegetation demographic models (VDMs), in contrast to DGVMs, more closely approximate real forest structure and demographic processes, allowing them to realistically simulate vegetation in a transient state (Friend *et al*., 1997; Moorcroft *et al*., 2001; Smith *et al*., 2001; Hurtt *et al*., 2002; Sato *et al*., 2007; Strigul *et al*., 2008; Fisher *et al*., 2015; Naudts *et al*., 2015; Haverd *et al*., 2018; Weng *et al*., 2019; Pugh *et al*., 2018; Argles *et al*., 2020; Ma *et al*., 2022; Weng *et al*., 2022). VDMs represent size and/or age‐structured ecosystem demographics, and in doing so resolve the processes of recruitment, growth and mortality. VDMs track either individual trees (Sato *et al*., 2007) or, more commonly, ‘cohorts’ (groups of individuals with identical properties, such as size, age and functional type, simulated as a single representative individual). Size‐based competition between these units (for light, water and nutrients) leads to an emergent forest structure. This structure is a property of the boundary conditions (climate and soil forcing variables, disturbances and human management), and plant physiological responses, determined by the tree's life history (e.g. small–large tree) and PFT traits that collectively determine growth rates and mortality. Overall, the unique feature in contrast to first‐generation DGVMs is that vegetation carbon emerges from these demographic processes and competitive interactions.

An increasing number of land‐surface modelling groups are working towards incorporating demographic vegetation into their models, with the aim of more realistically representing vegetation dynamics in the land component of Earth System Models (ESMs; Bonan & Doney, 2018; Bonan *et al*., 2024). These are not yet standard components of ESMs (e.g. Friend *et al*., 2014; Fisher *et al*., 2018; Fisher & Koven, 2020); however, with the exception of EC‐Earth (Döscher *et al*., 2022) and GFDL (Shevliakova *et al*., 2024). Furthermore, while international benchmarking systems for land‐surface models exist (Eyring *et al*., 2016; Collier *et al*., 2018; Melton *et al*., 2020; Abramowitz *et al*., 2024), they are not yet prepared for the types of outputs delivered by demographic models (e.g. forest structural variables, such as tree size distribution, and dynamic variables, such as growth and mortality rates).

So far, a multi‐model benchmarking exercise on VDMs has been lacking, possibly due to several challenges we faced during the course of this work: the large variety of model philosophies, the need for identical definitions of output variables, (data available for multi‐variable constraints (to avoid compensating effects during calibration), and benchmark definitions that are both ecologically plausible and model‐inclusive. Here, we make the first combined assessment of how nine VDMs used in this study perform against observations and explore the pathway to developing effective benchmarking. Simulations cover the regrowth trajectory and mature forest state at each of a boreal, temperate and tropical site. We compare models against: a biome‐wide chronosequence of carbon content during forest regrowth, plot‐level mature forest carbon content, plot‐level forest structure, plot‐level growth and mortality rates and biome‐wide self‐thinning relationships. We discuss: the similarities and differences between models and between models and observations. Finally, we highlight process uncertainties and make recommendations on critical benchmarking variables and observations going forward that could substantially reduce uncertainty in modelling of forest futures.

## Materials and Methods

### Models

Nine DVMs (BiomeE, BiomeEP, CABLE‐POP, EDv3, ELM‐FATES, JULES‐RED, LPJ‐GUESS, ORCHIDEE and SEIB‐DGVM), capable of global simulations were used to simulate forest regrowth and mature forest dynamics, emergent from forest structures under constant repeated 30‐yr climate cycles.

Model differences relevant to this study are highlighted in Table 1. More model‐specific information can be found in Supporting Information Methods S1. Model‐specific PFT‐species mapping is found in Table S1, and model‐specific modes of biomass reduction are found in Table S2.

**Table 1:** Key differences in model structure and assumptions that are relevant in this project.

Process\model	BiomeE	BiomeEP	CABLE‐POP	EDv3	ELM‐FATES	JULES‐RED	LPJ‐GUESS	ORCHIDEE	SEIB‐DGVM
Self‐thinning prescribed or emergent	Emergent	Emergent	Prescribed	Emergent	Emergent	Emergent	Emergent	Prescribed	Emergent
Initial recruits postdisturbance	Restocking	Restocking	C from atmosphere. function of existing biomass (growth suppression)	Restocking	C from seedbank with seedlings, restocked from NPP‐turnover with decay‐rate	C from extern npp. Number of saplings emerges	Sapling C: from estimate of first year's productivity. Sapling size depends on first year's productivity+ PFT allometry. Sapling count: stochastic	Saplings, from C and N from atmosphere	C from litter; initial size always 0.01 m dbh; sapling count stochastic
NPP (productivity) – level	Cohort	Cohort	Whole canopy	Cohort	Cohort	Whole canopy	Cohort	Cohort	Individual
Canopy structure (based on Fisher *et al*., 2018)	Flat top crown with prescribed maximum crown LAI; layering according to tree height and crown area (PPA)	Flat top crown with prescribed maximum crown LAI; layering according to tree height and crown area (PPA)	External	Flat top crown with prescribed maximum canopy height; layering according to tree height	PPA with leaf layers	External	Flat top crown, but cohorts/PFTs are vertically overlapping/multilayer	Aggregated individual crowns; layering according to tree height; shade depends on solar angle	Individual crowns represented by cylinder shape
Light competition	Within and between PFT and cohort; explicit	Within and between PFT and cohort; explicit	Within PFT; implicit	Within and between PFT; explicit	Within and between PFT and cohort explicit	Within and between PFT; implicit.	Within and between PFT and cohort; explicit	Within PFT; explicit	Within and between PFT; explicit
Water competition	Between PFTs and cohorts	Between PFTs and cohorts	Extern	Between PFTs and cohorts	Between PFTs and cohorts	Extern	Between PFTs	Between PFTs	Between PFTs
Nutrient competition	Between PFTs and cohorts	Between PFTs and cohorts	Extern	Between PFTs and cohorts	Optional (not simulated in these simulations)	Extern	Between PFTs		Not available
Active mortality mechanisms	Density‐dependent, density‐independent	Density‐dependent, density‐independent	Density‐dependent, background	Density‐dependent, density‐independent	Density‐dependent, background, stress‐based	Background	Density‐dependent, stochastic; density‐independent, stochastic	Density‐dependent, density‐independent, background mortality	Density‐dependent, density‐independent, background mortality
Vegetation unit	Cohort	Cohort	Cohort	Cohort	Cohort	Cohort	Cohort	Cohort	Individual trees for woody PFTs, cohort for grass PFTs

For example, while some models simulate harvest, this is not listed here. Initial recruits postdisturbance: Model‐specific settings and assumptions determine the initial size and relative composition postdisturbance and hence influence carbon regrowth trajectory. Here, carbon for new recruits is normally taken from different sources to ensure carbon budget closure (see restocking). Recruit size is either prescribed or emerges. Recruit number normally emerges. Species composition (i.e. what was allowed to grow) was prescribed in this project. Where the term *restocking* was used: recruit number and size were forced, by resetting to the initial cohort structure, so these recruits were not dependent on previous conditions, such as PFT productivity/seedbank strength/litter size. Self‐thinning: *prescribed* self‐thinning can be thought of as a function that relates mortality to the number density and size of individuals. *Emergent* can be thought of as a mortality rate that increases when light conditions/productivity decline, likely caused by the presence of too many trees. NPP (productivity)‐level: *Whole canopy structure* indicates that productivity is calculated for the entire canopy as an integrated unit, which may include vertical light gradients and sunlit/shaded leaf distinctions, but does not separately track productivity for individual cohorts or size/age classes within PFTs. *Cohort:* often a ‘tree‐level productivity’, upscaled to cohort with stem density. Canopy structure: uses expressions from Fisher *et al*. (2018), to distinguish between the ways vertical light distribution and thus light resource acquisition/competition/crown organisation. *Individual*: light profile on every individual differs, shading can also occur from an angle. *PPA*: Crowns self‐organize into discrete canopy layers, within which all plants receive the same incoming radiation. The lower layer trees' light is determined by the leaves in upper layers. *Flat‐Top*: the total leaf area of each cohort is conceptually distributed evenly across the whole patch and can be very thin. Problem: marginally taller cohorts outcompete their neighbours in terms of light availability. Nuances of this approach to address this are described in the table. Light competition: *Implicit* – competitive effects approximated through empirical or other relationships without mechanistic calculation of light interception. *Explicit* – competitive advantage determined by directly modelling light absorption and carbon gain differences between cohorts. Active mortality mechanisms: density‐dependent (e.g. competition for resources, e.g. light (or carbon), water and nutrients); density‐independent (related to the individual: e.g. size or age); stress‐based mechanisms that are not density‐dependent (e.g. frost, heat or cavitation mortality); and background (e.g. a constant rate). Some demographic models do not treat all processes themselves, which is marked by ‘external’. For more model‐descriptions and references, see Supporting Information Methods S1; Tables S1, S2.

Comparisons of all these contrasting VDM structures are in many ways nontrivial, as there are many definitional and philosophical differences between their approaches. In order for these models to become a more mainstream feature of, for example, operational model intercomparison studies, it is necessary to find ways to consistently compare them both with each other and with data. For the purposes of this comparison study, for example, many models created bespoke output variables, such as gross woody biomass increment. We defined and performed a woody carbon budget balance check for each model to test for ‘demographic’ woody carbon balance (Methods S2; Eqn S1; Fig. S1). Not all modelling groups were able to implement all variables according to the ‘D‐BEN’ (Demographic benchmarking) format (see table S3.1 in Notes S1 for the full list of variables simulated); where variables deviate, it is reported in Table S4. This study provides a platform for the first comparison of this type, and we anticipate that it will pave the way for subsequent efforts using a wider range of data products and emerging models.

### Model simulations

Models were run following a common simulation protocol (Notes S1). Simulations were performed at stand level where possible, in order to be comparable against the observations. Simulations were performed for three locations representing three major forest types (boreal, Finland (FIN): 62.25 N, 23.25 E; temperate, Bialowieza (BIA): 52.75 N, 23.75 E; tropical, Barro Colorado Island (BCI): 9.25 N, −79.75 E). Simulations involved a spin‐up period (according to each model's own procedure), followed by 30 yr of equilibrium forest conditions. After this, all trees were killed, removed or reset (Table S2), and the forest was allowed to regrow for at least 420 yr or until equilibrium was reached (Table S5, column ‘lower’). PFTs were chosen to reflect the biome's/site's vegetation composition as closely as possible (Table S1). Observations of woody biomass regrowth dynamics, mature state woody biomass and forest structure were made available *a priori* for potential calibration. Some models were calibrated (by hand‐tuning or direct parameterisation from observations), but modified parameters stayed within plausible ranges. Which models were recalibrated and their updated parameters are reported in Table S3. The meteorological forcing data used were a repeated 30‐yr‐long randomised climate time series (Notes S1) for 1991–2020 from CRUJRA v.2.2 (University of East Anglia Climatic Research Unit, 2021), and carbon dioxide (CO_2_) levels were kept at 2020 levels at 412 ppm. Where applicable, models set nitrogen deposition values at constant 2015 levels specific to the modelled site: 5.01 kgNha^−1^ yr^−1^ (FIN), 10.07 kgNha^−1^ yr^−1^ (BIA) and 3.57 kgNha^−1^ yr^−1^ (BCI), based on Lamarque *et al*. (2010).

### Observations

We investigate a set of six benchmarks specific to VDMs and assess their contrasting structural and process assumptions. Forest size structure: this integrates assumptions on allometry (how carbon is partitioned among individuals of different sizes) and demographic processes representation (establishment, growth and mortality). Woody growth fluxes: these interact with tree allocation and can highlight biases in productivity assumptions and are themselves influenced by vertical forest structure and individual‐tree size. Mortality fluxes: these influence carbon turnover and shape forest dynamics. For example, the tree size at the time of death can have benign (small tree) or profound (large tree) effects on subsequent forest structure, dynamics and thus the overall carbon pools. Self‐thinning: this is an emergent density‐dependent mortality process that can serve as a multi‐constraint on productivity‐mortality compensatory effects in conjunction with ‘tree/carbon packing density’ in the simulated forest: The self‐thinning rule describes the relationship between mean individual biomass (*M*) and the number density (*N*) of trees in an even‐aged forest with: *M* = *k N* ^*p*^, with k being a species or environment‐specific constant. The exponent, *p*, has been found to vary around the originally proposed value of −3/2, due to factors like species, climate, provenance and stand history (Pretzsch, 2006; Comeau *et al*., 2010) or depending on the theoretical model applied (Mrad *et al*., 2020). We work on the assumption that self‐thinning operates as a general ecological process also in mixed‐species forests (Pillet *et al*., 2018). Carbon stock changes during regrowth: biomass (carbon) regrowth curves capture the net effect of forest dynamics during recovery after a disturbance, testing the ability of the VDMs to capture a phase in which many forests globally currently are. How the interplay of forest structure, mortality and growth differs during forest recovery and the mature forest state. During recovery after a strong disturbance, structure is initially homogeneous, but growth and mortality fluxes are overall very unequal and variable in time, whereas in mature forests, growth rates and mortality fluxes are relatively equal overall. A demographic model has to be able to replicate these diverse emergent properties and fluxes over the period of forest recovery.

Together, these benchmarks capture key interactions between growth, mortality and allometry, forming a robust multi‐constraint dataset for demographic model evaluation. For model calibration and comparison, we used multiple types of datasets, biome‐level regrowth curves of aboveground woody carbon (C_wood,AG_) and mass‐number density data as well as site‐level mature forest C_wood,AG_ dynamics and stand structure information. Besides the benchmarking datasets used in this study, VDMs uniquely enable the use of specific additional data constraints (Table 3) which are highly abundant but incompatible with nondemographic models.

#### Site descriptions

FIN: data were obtained from 57 conserved or low‐intensity managed (last management > 30 yr ago) sites in central and southern FIN (Peltoniemi & Mäkipää, 2011).

BIA: five permanent monitoring plots, spread across Białowieza National Park, situated in northeast Poland (at 52.500 to 53.000 N, 23.833 to 24.250 E), with a combined area of 15.44 ha (Brzeziecki *et al*., 2016).

BCI: BCI is a 50‐ha ForestGEO research plot in Panama (9.1543 N, −79.8461 E; Condit *et al*., 2019; Davies *et al*., 2021). It is located in a lowland tropical moist forest and contains *c*. 240 000 stems of 300 species of trees and shrubs with diameter at breast height (DBH) ≥ 1 cm.

#### Mature forest C_wood,AG_ dynamics

**Fig. 1:**
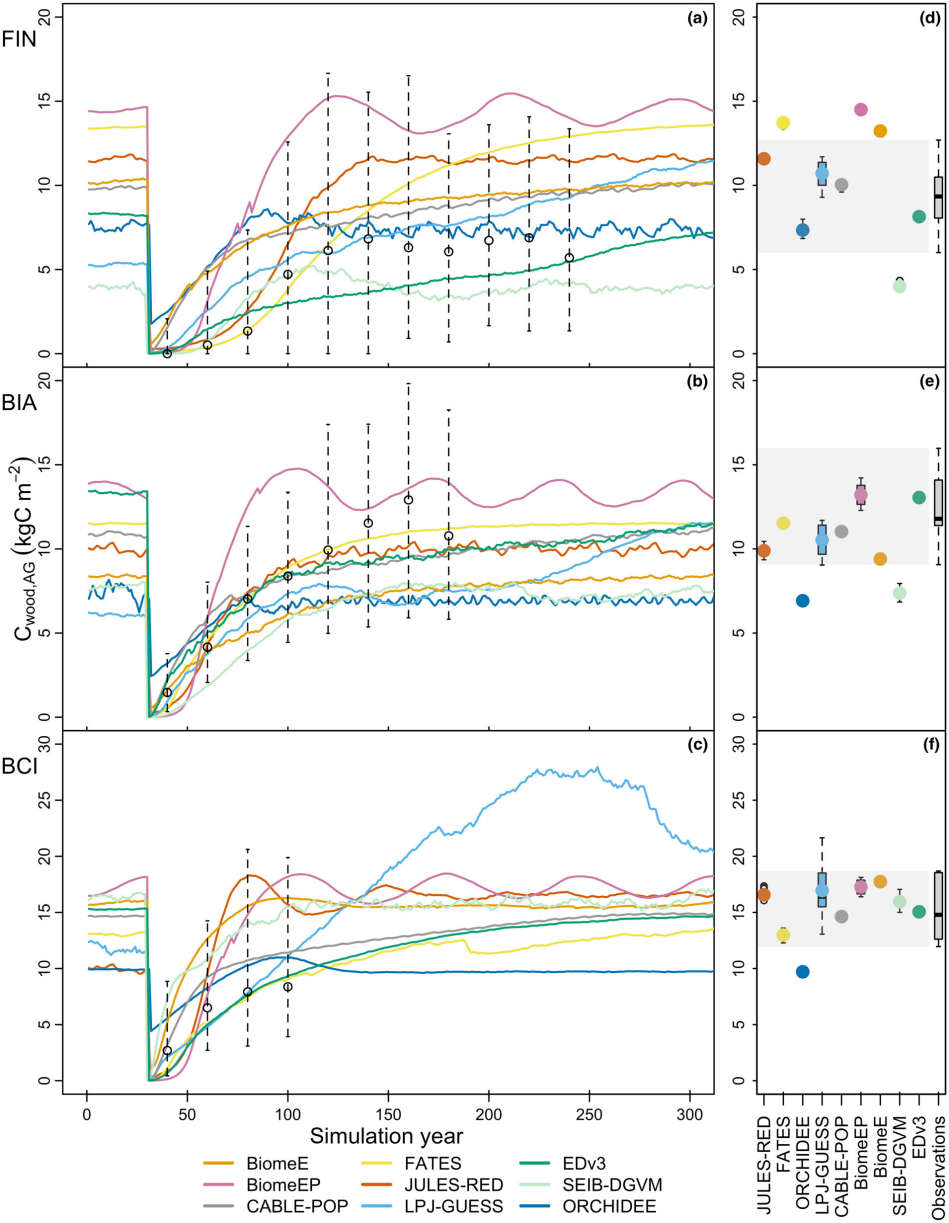
Comparison between aboveground woody carbon (C_cwood,AG_) recovery (a–c) and aboveground woody carbon at mature forest equilibrium (Supporting Information Table S5) (d–f) after stand‐replacing disturbance of the forest at a boreal (FIN), temperate (BIA) and tropical site (BCI). (a–c) Observations (black) of age bins of C_cwood,AG_ during regrowth and FIN mature forest C_cwood,AG_ are reported as median, and the whiskers end at the 10^th^ and 90^th^ percentile boundaries (*n*(age bins) or *n*(mature forest plot) > 20). (d–f) Mature forest C_cwood,AG_ observations at BCI are taken from median and upper and lower confidence interval values from spatial bootstrapping (6.25 ha) of a 50 ha plot with 95% confidence interval, across four repeated censuses. For mature forest at BIA, median, maximum and minimum observation values of five plots across six censuses were used. Mature forest at FIN observational range is derived from median, 10^th^ and 90^th^ percentile ranges from five census years. Model output at mature forest equilibrium was taken from a varying length of time series, depending on the model (Table S5). The time to equilibrium differed between models, and we also report individual model output graphs for their full time series in Notes S4. BCI, Barro Colorado Island; BIA, Bialowieza; FIN, Finland.

The nature of the three C_wood,AG_ (kgCm^−2^) datasets used for site simulations was diverse, ranging from a single site (BCI) to over five sites within a national park (BIA) to a multitude of largely unmanaged sites across southern and central FIN. As the plot sizes, number of plots and frequency of measurement differed greatly at the sites, the approach to obtaining variability ranges also differed by site. We report the number of data points used to derive each benchmark in Table S6 and all data processing in Methods S3. Mature forest benchmarking observations are shown in Fig. S2 and are used in a more aggregated form in Fig. 1.

#### Mature forest woody turnover time (*τ*) and woody biomass growth (WBgrowth)

The observations were aggregated from individual‐tree measurements to stand level and based on the above‐described census data from the sites. Calculations are described in Methods S3; we made use of all datapoints for the comparison.

#### Stand structure

DBH data from the same three sites as above were binned into dbh classes of < 1 cm, < 5, < 10, < 15, < 20, < 30, < 40, < 50, < 60, < 70, < 80, < 90, < 100, < 150, < 200 and ≥ 200, corresponding to simulation output. BCI, FIN and BIA report saplings starting from 1, 5 and 10 cm DBH, respectively, which map onto our dbh‐class categories (‘< 5’, ‘< 10’ and ‘< 15’).

Stand structure data for number of stems per dbh class (*n*stem_size), as well as aboveground woody carbon biomass per size class (cwood_size) are reported. To obtain an upper and lower observational range for BCI, we performed bootstrapping (Table S6) and report the 95% confidence interval and median for each dbh class for the latest year recorded (2015). For BIA, we report the median of five sites, alongside the minimum and maximum across all sites for each dbh class for the latest year recorded (2012). For FIN, we report median, maximum and minimum of 57 sites, from the census conducted in years 2006–2007. Stand structure benchmarking observations are shown in Fig. S3.

#### Biome‐level C_wood,AG_ regrowth curves

Regrowth curves were not available at the site level and were instead derived from chronosequence data. For the tropics and temperate regions, we used precompiled data from Teobaldelli *et al*. (2009) and Poorter *et al*. (2016), respectively. For boreal biome‐level data, we used biomass chronosequence data from conserved low‐intensity management (last management > 30 yr ago) stands in FIN (Central and South FIN regions; Korhonen *et al*., 2021). More detailed postprocessing is described in Methods S3. The data's spatial extent is shown in Fig. S4. For all biome datasets, C_wood,AG_ values were organised in 20‐yr age bins, and we report the median, lower 10^th^ percentile and upper 90^th^ percentile boundary for these bins. At least 20 observations are in each bin, following Pugh *et al*. (2019). The resulting C_wood,AG_ regrowth chronosequences cover 220, 160 and 80 yr for FIN, BIA and BCI, respectively (Figs 1, S2).

#### Mass‐number density data for self‐thinning‐benchmarking

Observations on C_wood,AG_ and number of stems (*n*stem) were extracted from chronosequence data in Teobaldelli *et al*. (2009). We selected for broadleaf groups and conifer groups for comparison against the BIA and FIN sites, respectively, and removed dbh classes < 10 cm DBH. Observations from Poorter *et al*. (2016) do not contain the information to create a mass–density scatter of their regrowing forests data. To visualise mass‐number density relationships of the observations, we calculated the logarithm of mean individual biomass log (C_wood_/*n*stem) and logarithm of *n*stem (Fig. 3a,b).

### Model data postprocessing

#### Identification of self‐thinning in models

To determine the self‐thinning slope in the models, we fitted a linear model to the natural logarithm of tree number (*n*stem) and mean tree biomass (C_wood_/*n*stem) for the subset of years during regrowth identified to be within the self‐thinning period, fitting a linear regression with the lm() function with default settings from the stats package in R Core Team (2023). Similar to the observations, we omitted dbh classes below 10 cm DBH, except for CABLE‐POP, where this dbh‐class distinction cannot be made in the current output. To determine the slope of the self‐thinning curve and compare to observations, it was necessary to determine which period of the simulation was relevant to the process of self‐thinning defined as even‐sized competition. The period was extracted based on a combination of criteria, depending on the model (Table S7). In models that provided diagnostic outputs for self‐thinning‐related mortality (e.g. CABLE‐POP, LPJ‐GUESS and ELM‐FATES), the selected period corresponds to when the self‐thinning mortality rate exceeds the 95^th^ percentile of annual self‐thinning mortality rates observed during the simulation (Method 1, Figs S5, S6). This approach ensures focus on periods when self‐thinning is the dominant mortality mechanism. For models with no explicit thinning‐related mortality mechanism, the thinning period was either (Method 2, Fig. S7) chosen as the period with the top 5% of total mortality rate (e.g. BiomeE, EDv3 for BIA), or (Method 3, Fig. S8) the consecutive points between the minimum and maximum densities, chosen semi‐automatically as the consecutive points between the furthest to the bottom‐left and the top‐right points in the time series in self‐thinning space (e.g. BiomeEP). Lastly, (Method 4, Figs S9, S10) the period was adjusted manually so that the trajectory of the simulation reflects what looks like a thinning trajectory (e.g. Enquist *et al*., 1998) but could not be achieved with the semi‐automatic approach of Method 3 (e.g. used for SEIB‐DGVM). More in‐depth explanations on the methods are found in Methods S4. Visualisations of the self‐thinning period as part of the whole simulation period for each model and each site are found in Fig. S11.

#### Forest phase classification

One notable feature of our comparison exercise is that the demographic models differed in their initial establishment conditions after the stand had been killed and therefore started their regrowth trajectory at different regrowth phases. We here defined additional qualitative ecological benchmarks, ‘forest phases’, which are distinct periods during forest regrowth describing the progression from open canopy to closed canopy. We use the forest phases benchmarks to meaningfully interpret the models' behaviour alongside each other during regrowth. In a given forest phase, a collection of variables, such as canopy area (CA), number of stems (*n*stems), mortality rate (cmort_rate_) and total vegetation carbon (C_veg_), must act together in a characteristic pattern that reflects the defining dynamics of that phase. For example, either small trees die in large numbers, detectable in the mortality flux, or they grow into larger trees, visible by them shifting over time from one size‐class category to another. The former indicates that self‐thinning is ongoing, whereas the latter indicates that there are still enough resources available for all trees to increase in size.

**Fig. 2:**
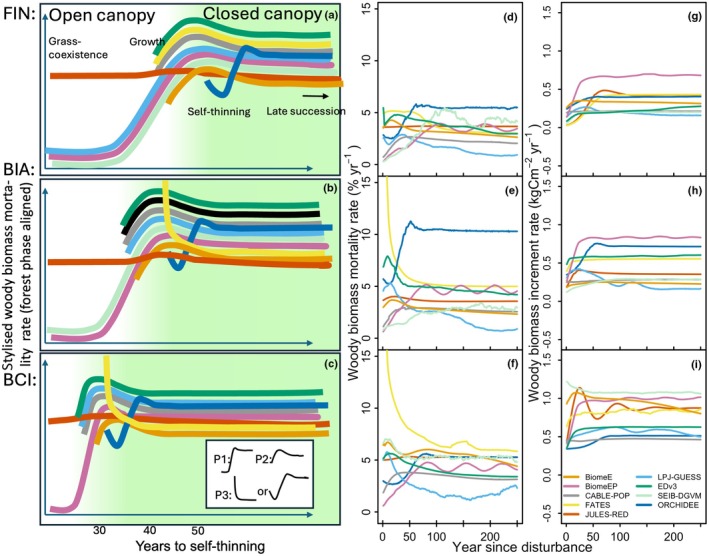
Stylised woody biomass mortality rates (a–c) aligned on the forest recovery‐phase timeline for FIN (a), BIA (b) and BCI (c). The years to self‐thinning shown on the x‐axis are an approximate ensemble mean for each site. Unaligned, 30‐yr‐smoothed woody biomass mortality rates (d–f) and unaligned, 30‐yr‐smoothed woody biomass increment rates (g–i). The different vegetation demographic models (VDMs) start their recovery after disturbance at different forest phases, but some mortality rate commonalities exist, expressed by the different shapes, representing each a different starting point or initialisation of regrowth postdisturbance: P1: mortality rate starts from low, nearly 0‐values, peaks and then either decreases or remains flat, caused by a model initialising in open‐canopy grass‐coexistence phase. P2: mortality rate starts from higher values and then either peaks or remains flat, caused by a model initialising in open‐canopy‐growth phase. P3: mortality is immediately initiated, which causes a decline towards an equilibrium or which causes a decline and relief‐period with less competition, until this peaks and stabilises (more detail in Supporting Information Methods S5), caused by a model initialised in closed‐canopy self‐thinning. The forest recovery phases are marked on the plots (a–c) as ‘grass‐coexistence’ and ‘growth’, during open‐canopy phase (white background) and a self‐thinning and a transition towards secondary successional forest and a dynamic equilibrium is shown through the arrow (green background) during closed canopy. The green background is in a soft transition not only to indicate the gradualness of the process but also to highlight a difference between gap‐models and PPA‐based models in the transition towards self‐thinning: where PPA‐models can allow for canopy closure, but growth to continue until canopy packing is optimised, whereas canopy closure in gap‐models initiates self‐thinning. Note that FATES mortality rates are derived from total biomass and total biomass loss. FIN, Finland; BIA, Bialowieza; BCI, Barro Colorado Island.

The characterisation framework we present in Table 2 was used to first determine which phase each model commences regrowth from (Table S9), followed by aligning the model output of growth and mortality rates along this common trajectory (Fig. 2). Interpretation of commonalities in growth and mortality rate dynamics was carried out once the model trajectories were aligned.

**Table 2:** Forest recovery‐phase classification necessary for forest phase alignment.

Phase	Variables involved/dominant	Description of dynamics in concert with auxiliary variables
Open‐canopy phase – presence of grasses	%C_veg_, *n*stems (*n* ha^−1^), CA (m^2^ ha^−1^), cmort_rate_ (% yr^−1^)	Caused by delayed onset in woody establishment, an open canopy (low CA) prevails with low *n*stems, higher fraction of Grasses in %C_veg_, and no tree–tree competition (low cmort_rate_)
Open‐canopy phase – growth	*n*stems (*n* ha^−1^), %C_veg_, $\text{cmor}t_{\text{rate}}$ (% yr^−1^), CA (m^2^ ha^−1^)	Rapid increase in CA and fraction of trees in %C_veg_; *n*stems are relatively stable, but will decrease, once cmort_rate_ increases (a gradual rise in cmort_rate_ may begin towards the end of this phase)
Closed‐canopy self‐thinning	cmort_rate_ (% yr^−1^), *n*stems (n ha^−1^), CA (m^2^ ha^−1^), %C_veg_, CAI (−)	A mortality (cmort_rate_) spike and steep decline *n*stems (n ha^−1^), where not all established individuals grow into a larger size class, and self‐thinning mortality occurs. Also indicated by canopy closure. CA *c*. 10 000 m^2^ ha^−1^ (but see Table S8)
Closed‐canopy late‐successional phase	%C_veg_, cmort_rate_ (% yr^−1^)	A shift in composition is evident in %C_veg_ of the PFTs present (where models simulate this) and cmort_rate_ can show a slight directional change in the trajectories, caused by both the PFT composition and/or the forest structure now shift into a secondary succession stage, moving towards a dynamic equilibrium

Description of variables that are indicative of certain phases in forest development, as found in the model ensemble. These definitions were used to re‐align the models to a similar forest state postdisturbance. The output time series of all variables per model and site are found in Supporting Information Notes S2 where modelling groups provided evidence and comments on the potential transition and starting points. CAI, crown area index. A more detailed description of forest phase classification, complementing this table, is provided in the Table S8.

#### Modelled mortality and growth rate postprocessing

The fractional mortality rate, cmort_rate_ (% yr^−1^) was postprocessed from the mortality flux, C_mort_ (kgC m^−2^ yr^−1^) and woody carbon, C_wood_ (kgC m^−2^):

(Eqn 1) $${\text{cmort}}_{\text{rate}}=\frac{C_{\text{mort}}}{C_{\text{wood}}}100$$

Stand growth rate was directly obtained from the model output as the growth increment flux, WBgrowth (kgC m^−2^ yr^−1^). For this time series analysis, we applied a 30‐yr smoothing on the mortality and growth increment flux to remove climate features from the repeated 30‐yr scrambled sequence. This approach also reveals the dominant patterns in the model dynamics. To better capture postdisturbance dynamics, we excluded the 30 yr immediately following spin‐up from the time series before applying the smoothing. We applied the function rollmeans (*k* = 30, align = ‘left’), with otherwise default settings from the zoo package (Zeileis & Grothendieck, 2005) in R.

Turnover time τ is given by:

(Eqn 2) $$\tau =\frac{C_{\text{wood}}}{C_{\text{mort}}}$$

For turnover time (Eqn 2) and mature forest woody growth flux comparison, we used annual output values from each model's respective equilibrium time period.

For comparison against observed values of turnover time and WBgrowth rate (Fig. 4), we did not smooth the output in this analysis, as smoothing the model time series over 30 yr would eliminate the temporal variability that serves as an approximation of the spatial variability captured in the observations. This would effectively remove the range depicted in the boxplots for many models in Fig. 4.

## Results

### Forest recovery

#### Carbon pools

Ecosystem carbon recovery of all VDMs was evaluated against observations across three biomes (Fig. 1). All models, with one or two temporary exceptions, fall within the observed range of variability of aboveground woody carbon (C_cwood,AG_) recovery dynamics (Fig. 1a–c). At FIN, modelled C_cwood,AG_ recovery is predominantly above the median observed recovery during all time intervals (seven out of nine models). At BIA, C_cwood,AG_ recovery is initially spanning the whole range (first 20 yr) and progresses to be generally lower than the observed median in the following 100 yr (seven to eight out of nine models). Similarly, at BCI models span the full range of variability in the first 20 yr postdisturbance, but then continue to C_cwood,AG_ values at or above the median (seven of nine models). Consistent model‐specific biases across all three sites, for example one model recovering consistently faster than the others, are not visible. Site conditions and PFT‐physiological (i.e. parametric) differences may cause some models to react more strongly than others (for instance), SEIB‐DGVM has one of the fastest C_cwood,AG_ recoveries in the first 20 yr postrecovery at BCI, and one of the slowest at FIN, whereas EDv3 recovers fast in the first 20 yr at BIA, but is in the mean to low model range after 20 yr at BCI. Regrowth speed does not seem to be linked to final equilibrium biomass values (Methods S7, Fig. S12). Initial C_cwood,AG_ wood levels postdisturbance differ across models, with ORCHIDEE being highest earliest at all sites. While the models fall within the range of the observations of C_cwood,AG_ at any given time point, there is significant variation both during recovery after disturbances and in mature forests among models. For example, after 30 yr of recovery, aboveground woody carbon ranges from 0.45 kgC m^−2^ to 5.3 kgC m^−2^ for FIN, 1.84 kgC m^−2^ to 6.10 kgC m^−2^ for BIA and 4.90 kgC m^−2^ to 12.84 kgC m^−2^ for BCI (see Table S10 for model‐specific values). In mature forests, equilibrium values range from 4.0 to 14.50 kgC m^−2^, 6.92 to 13.21 kgC m^−2^ and 9.71 to 17.74 kgC m^−2^, depending on the model (Table S10).

#### Woody growth and mortality rates during recovery

Dynamics of woody biomass increment rates and woody mortality rates are highly variable between VDMs and across the first 250 yr after disturbance (with the least variability observed in JULES‐RED).

In terms of mortality dynamics (Fig. 2d–f), the VDMs investigated here show a change in mortality rate over the course of stand development (largest change 23% yr^−1^, mean change 4% yr^−1^, smallest change 0.8% yr^−1^). Modelled mortality rates are highest at different timings of regrowth, but more commonly highest during early regrowth phases. These higher mortality rates during establishment and regrowth differ also in their magnitude relative to a model's dynamic equilibrium that follows (not shown in the stylised graphs in Fig. 2a–c, but visible in Figs 2d–f, S13–S15), and the rate of decline of mortality rate towards the equilibrium is also different (Fig. 2d–f).

In terms of growth dynamics (Fig. 2g–i), a similar picture emerges, with initially increasing woody growth increment rates for all VDMs, which normally peak or flatten early during regrowth. Models differ a lot, however, in the magnitude of change in growth rate during the first 250 yr, the amplitude of which ranges between 0.05 and 0.6 kgC m^−2^ yr^−1^ depending on the VDMs. Even in shorter periods, such as 30 yr directly after disturbance, model annual growth rates can span 0.03–0.60, 0.18–0.80 and 0.35–1.10 kgC m^−2^ yr^−1^ for FIN, BIA and BCI, respectively (Table S10).

To meaningfully assess the differences in simulated dynamics during forest regrowth, we realign mortality rates (% total C_cwood_ yr^−1^) of the VDMs during the first 250 yr of disturbance recovery (Fig. 2) and put them in context with prevailing forest recovery phases (Tables 2, S8, S9, S11; Methods S5).

Models begin their forest recovery at different phases of the regrowth trajectory and can be roughly divided into three groups (Fig. S16): Group 1: ‘open canopy – grass coexistence’ (BiomeEP, SEIB‐DGVM, JULES‐RED and LPJ‐GUESS at FIN): relatively low mortality rates prevail initially (but can also be high if a model's saplings are allowed to compete with grasses or have a high mortality rate), and a significant presence of grasses is allowed due to either the low density and/or size of the first saplings. Group 2: ‘open canopy/growth’ (CABLE‐POP, EDv3, SEIB‐DGVM at BCI, BiomeE, LPJ‐GUESS at BIA and BCI): seedlings are established, and grass may be present but not as a significant fraction of vegetation biomass (%C_veg_; note again that there is no between‐PFT competition in CABLE‐POP and ORCHIDEE, so the mechanism that reduces the %C_veg_ of grasses is purely the increase in woody PFT biomass, rather than the competition between woody PFTs and grasses). The length of this phase varies substantially between models. Group 3: ‘closed canopy/self‐thinning’ (FATES at BIA and BCI, ORCHIDEE): while FATES forest phase identification (Table S9; Notes S2) shows a clear open‐canopy grass‐coexistence period, followed by an open‐canopy‐growth phase at BIA and BCI, their combined time is 20 to 6 yr respectively, and the dominant feature visible in the smoothing is the self‐thinning mortality; therefore, FATES is here reported in group 3.

#### Self‐thinning

**Fig. 3:**
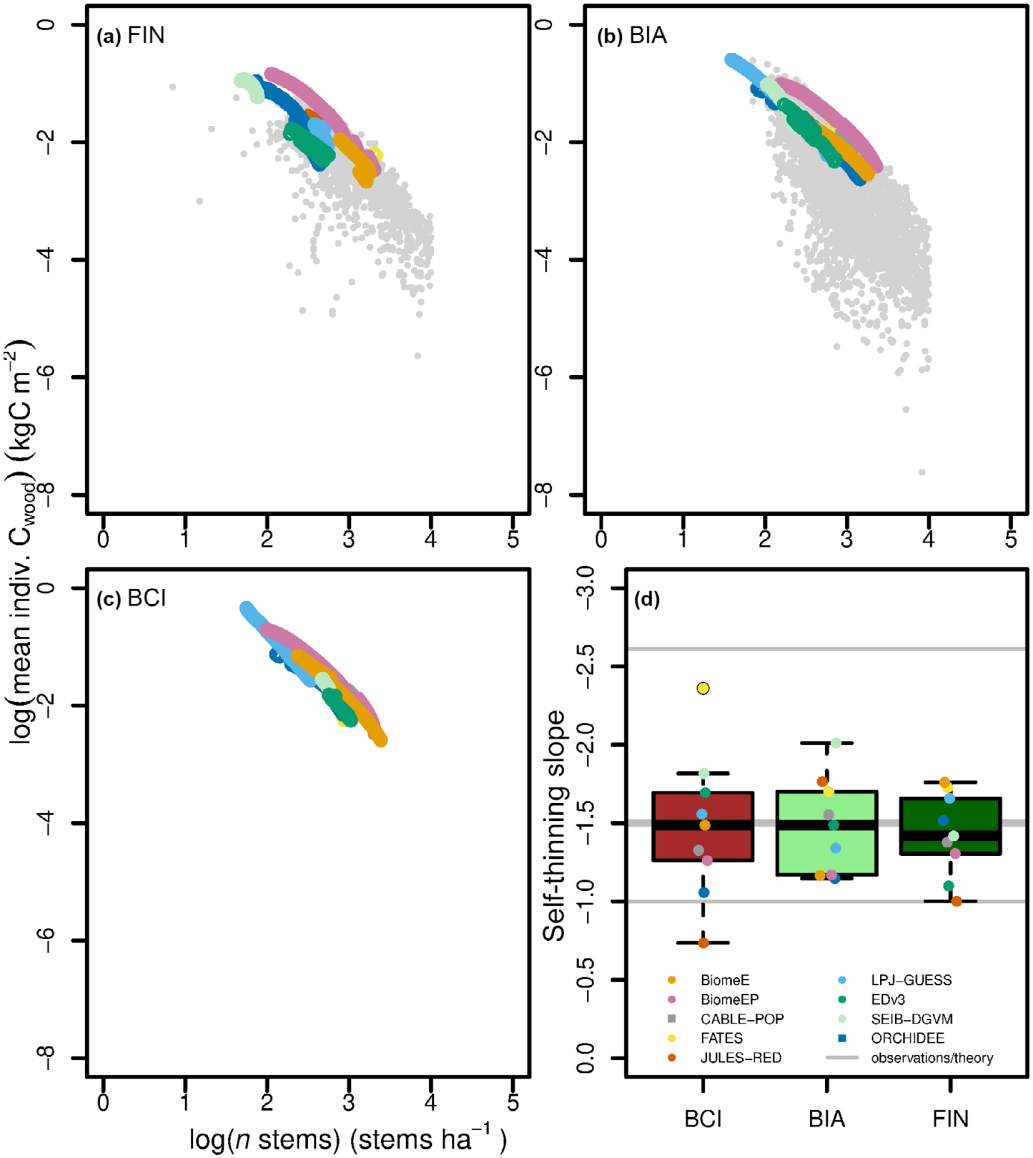
Model output on average‐individual woody biomass and stem numbers in ‘self‐thinning space’ (a–c) and model self‐thinning slopes (d) for all three sites. Grey dots in (a) and (b) are observed mass–density relationships of regrowing temperate and boreal forests, taken from Teobaldelli *et al*. (2009), filtered by tree size and stand age (> 10 dbh and > 10 yr). The coloured dots are the model‐specific subset of the period of regrowth where the trajectory coincides with thinning behaviour (see also Supporting Information Fig. S11). These points were then used to fit the self‐thinning line/slopes of each model, which are reported in the boxplot in (d) in context with the self‐thinning slope – 3/2, and observed lower (−2.612; Pretzsch, 2006) and theoretical higher (−1; Mrad *et al*., 2020) thresholds, shown as grey lines. Boxplot whiskers extend to 1.5× interquartile range. Self‐thinning slope values are also reported in Table S7. Models with square instead of circle dots in (d) have prescribed or implicit self‐thinning mortality.

Self‐thinning trajectories for most models are aligned along the upper boundary of the observations of observed mass–density relationships for BIA, and more spread at FIN (Fig. 3a,b). BiomeEP, CABLE‐POP and FATES' self‐thinning trajectories are the closest to the observed edge of mass‐number density scatter. The number of points per model varies (Fig. 3a–c), representing the years the regrowing forest spent in a period of thinning. Thinning period duration varies across models and sites (BCI: ensemble mean: 44.75, min: 12, max: 98 yr; BIA mean: 70, min: 30, max: 128 yr; FIN mean: 80, min: 38, max: 175 yr; Table S7), but there is a general trend to fewer years of thinning at BCI. There is a mass‐number density space in the observations with a high number of small trees and low mean individual mass which models do not occupy. Self‐thinning slopes that emerge from the self‐thinning lines are reported in Fig. 3d and fall within observed/theoretical ranges. There is no systematic behaviour visible for any model for only three sites in terms of self‐thinning slope, but the ensemble spread is largest in the tropics and the ensemble median closely resembles the theoretical self‐thinning slope.

### Mature forest

#### Carbon pools

Simulated mature forest C_cwood,AG_ (Fig. 1d–f) either displays a very stable equilibrium (dots cover the boxplot for FATES, BiomeE, BiomeEP, CABLE‐POP, EDv3, JULES‐RED at FIN) while other models exhibit greater variability over time (whiskers are visible for LPJ‐GUESS, SEIB, BiomeEP at BIA, JULES‐RED at BIA and BCI). Models' mature forest C_cwood,AG_ lies largely within the range of observations, with the highest number of models (*n* = 3) deviating at FIN and BIA (where in the latter case all models underestimate mature forest carbon).

#### Forest structure

**Fig. 4:**
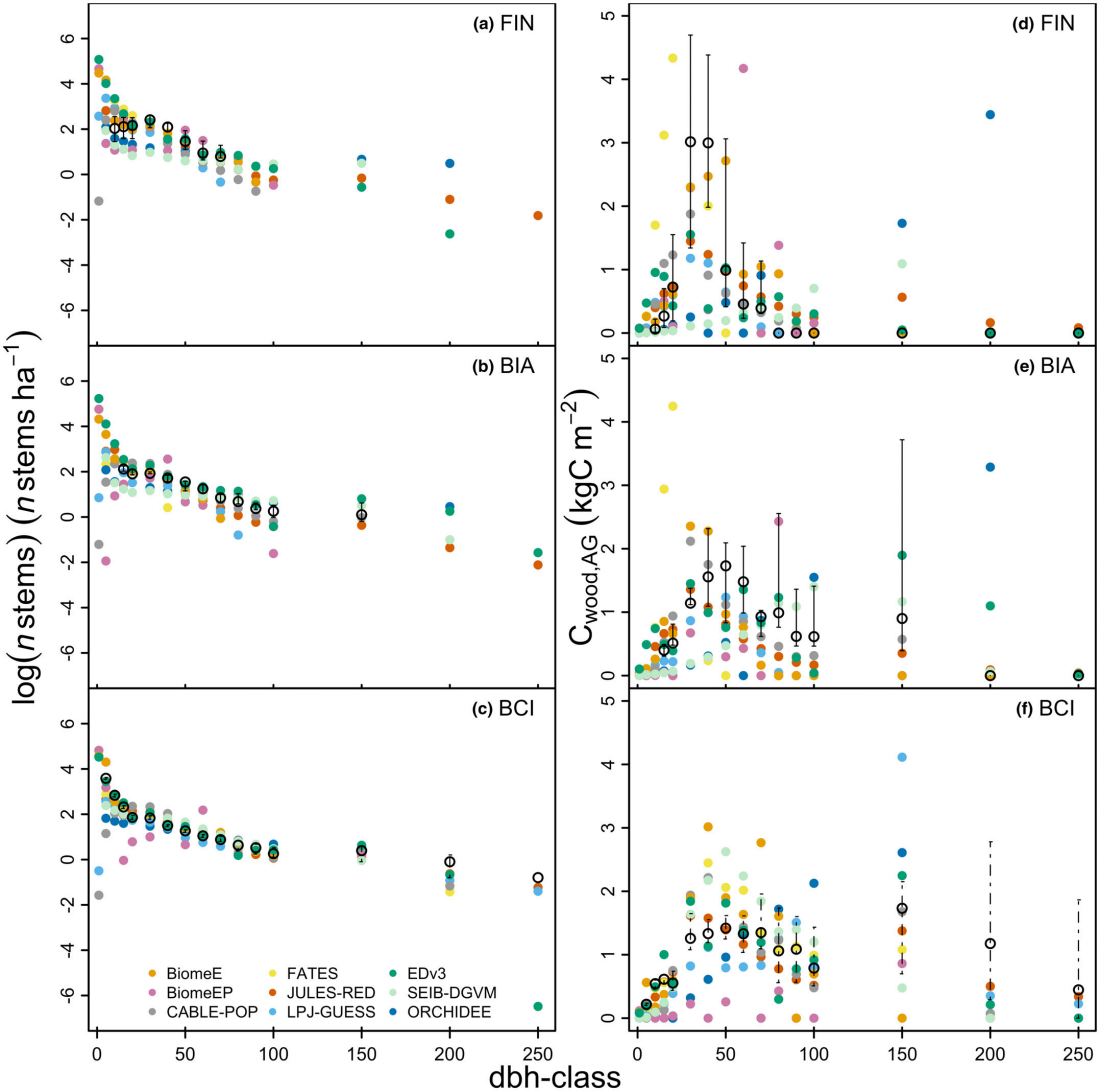
Mature forest structure in terms of number of stems across multiple diameter at breast height (DBH) size‐class categories (‘dbh‐classes’) (a–c) and aboveground woody carbon (C_cwood,AG_) across multiple DBH across multiple size classes (d–f) against a boreal (FIN), temperate (BIA) and tropical (BCI) site. Observations (black): the median (open black circles) and whisker boundaries are derived using different methods: BCI is derived from spatial bootstrapping of a 50 ha plot and reported as with a 95% confidence interval (Supporting Information Table S6). For FIN, the range shown is the 10^th^ and 90^th^ percentile of binned size classes across multiple plots, and for BIA, we use the minimum and maximum available plot (*n* = 5) values. Note that LPJ‐GUESS output origin deviates from the ensemble (Methods S6; Fig. S17). Model‐specific individual plots can be found in Notes S3. BCI, Barro Colorado Island; BIA, Bialowieza; FIN, Finland.

The character of the stem number distribution across DBH size‐class categories (‘dbh classes’) is generally captured in the models (Fig. 4a–c; Notes S3), although some models tend towards too low densities at FIN and BIA. The exception is the smaller size classes, the densities of which are commonly overestimated, apart from the tropical site BCI, where they are underestimated by the majority of models. Among the three sites, the general trends in tropical stem number distribution are captured best, while the highest number of models falls within the observational range at FIN, where variability in the observations is largest. Models tend to predict larger trees than are observed at FIN and BIA (0 trees observed at dbh class 200 and 250), and underestimate tree numbers at BCI at dbh class 200 and 250, which manifests in high C_cwood,AG_ in trees at FIN and BIA, but less than is observed at BCI. The general trend in forest structure (i.e. a high peak of C_cwood,AG_ in the 40–50 DBH class, followed by a marked decline at FIN, a gradual decline after a peak at 50 DBH at BIA, and a gentler decline at 50 DBH at BCI) is generally replicated by the ensemble, if not by the individual models. ORCHIDEE and SEIB underestimate C_cwood,AG_ in small‐to‐medium tree size classes while overestimating it in large classes. BiomeEP ‘oscillates’ in the number of stems at size classes below 100 DBH (i.e. some DBH classes can have 0 trees), and FATES has a small‐tree bias at FIN and BIA, both in terms of stem numbers and C_cwood,AG_ in stem.

#### Growth fluxes and carbon turnover time

**Fig. 5:**
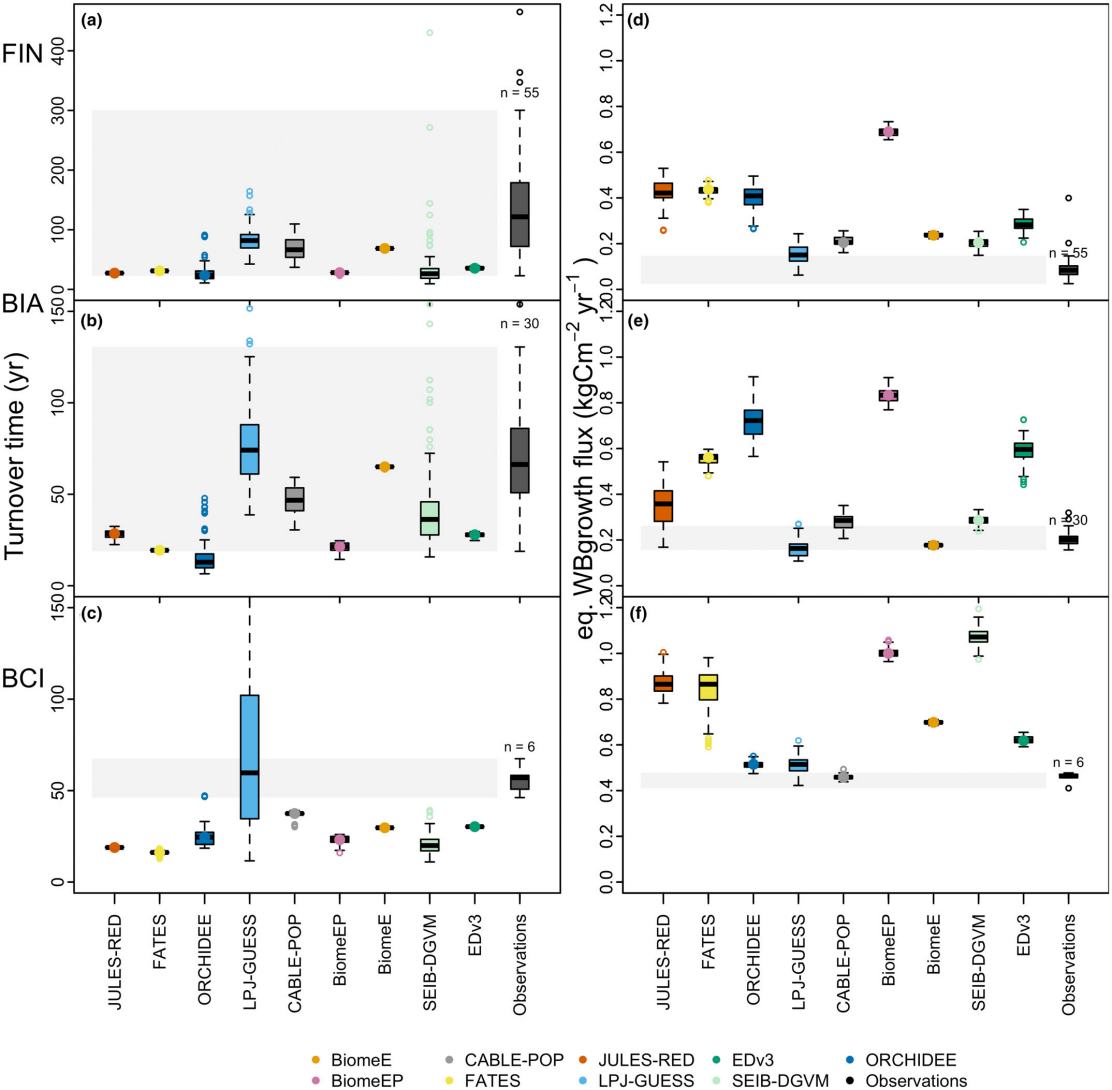
Simulated and observed annual mature forest turnover time (a–d) and woody biomass increment flux (e, f) at a boreal (FIN), temperate (BIA) and tropical (BCI) site. Benchmark boxplots consist of observations from each year available and each plot available (e.g. five plots at BIA × 6 census years; 1 plot at BCI × 6 census years; and a total of 55 plot‐year combination at FIN). Note that FATES includes nonwoody growth and mortality in its turnover variables plotted here. For BCI, the grey polygon shows the full data range. The upper whisker (1.5× interquartile range) value for LPJ‐GUESS boxplot in (c) is 193.34. BCI, Barro Colorado Island; BIA, Bialowieza; FIN, Finland.

Observed ecosystem woody growth fluxes and turnover times from three mature forest sites were compared against model output (Fig. 5). Most deviation from the observations is visible at the tropical site BCI, where models overestimate the growth flux and in turn underestimate the turnover time. For BIA, most VDMs are within the observational variability of the turnover time, but this is more a feature of the high variability of turnover time recorded across the five plots than of a well‐constrained model response. Generally, models at BIA cluster below the median turnover time of the observations and above the observational range for woody growth rate, as at BCI. Lastly, at FIN, the wide observational range allows more models to fall within growth increment flux variability, though similar to BIA, the majority of the models are below the median turnover time, and all models are above the median growth rate (Fig. 5d). VDM woody growth fluxes are generally increasing from the boreal to the tropical site, similar to the observations. BiomeE, LPJ‐GUESS and CABLE‐POP most often fall within the range of observations. Overall, gross woody growth increment (kgCm^−2^  yr^−1^) is overestimated by the majority of the models at all sites (up to 0.6 kgCm^−2^ yr^−1^), and turnover time is within the range of the variability or underestimated.

## Discussion

### Benchmarking forest recovery

While within the observational variability, VDMs differed significantly in the timing and speed of recovery following disturbance, especially in the first *c*. 30 yr (Fig. 1). We were not able to benchmark growth rates during the recovery phase due to a lack of suitably resolved observations. However, the large variability and overestimation in mature forest growth rates (Fig. 5d–f) are highly likely to be representative of the behaviour of the VDMs throughout the recovery phase, leading to the divergent forest carbon recovery trajectories in Fig. 1.

Furthermore, early recovery differences are caused by VDMs starting their regrowth trajectory from different forest ‘initial establishment conditions’ (open‐canopy grass coexistence, open‐canopy growth or immediate thinning conditions; Fig. 2). Once we aligned the forest recovery phases, the general dynamics (i.e. high mortality rates during self‐thinning, high growth rates before canopy closure) of the models are broadly similar, suggesting that important emergent ecological behaviours are captured. However, the underlying processes governing the timing of the start and end of the phases and the magnitude of growth and mortality rates are in need of further development (Fig. 2).

We note that successional dynamics are probably a further contributor to the differences in forest recovery dynamics, but due to a lack of data, we did not address this within this study. Plausible succession behaviour from shade‐intolerant to shade‐tolerant is visible in several models (Fig. S18), but the duration of their presence and degree of coexistence with late‐successional PFTs varies, which can greatly contribute to different forest structure or turnover time, while the overall C_cwood,AG_ may be similar. This is a source of uncertainty in VDMs that requires benchmarking attention.

Growth and mortality rates during recovery show plausible dynamics in all models. In terms of growth rates during recovery, VDMs replicated some or all stages of ‘boom‐and‐bust’ growth dynamics described for tropical forests by Chave *et al*. (2020). After canopy closure, woody productivity decreases or remains at a stable peak, depending on the model. Similarly, observed stand‐level production increases in Chave *et al*. (2020) throughout the 40 yr of record but declines sharply in the smaller size classes following canopy closure after 10 yr. In the models, the reductions in simulated woody productivity at the stand level could generally be attributed to some combination of increased respiration, changes in allocation of carbon to woody tissues as opposed to leaves, fine roots or reproduction, and ultimately changes to GPP due to community‐weighted trait changes or due to height increases and succession in PFTs over time during development. For example, the two‐phase pattern in growth rates in LPJ‐GUESS and BiomeE is caused by a change in PFT‐species composition with different physiologies (more clearly seen in Fig. S19).

Similarly, and in concert with growth rates, simulated mortality rates change over time in the stand as a result of competition. This is supported by observations of both temperate (Pretzsch *et al*., 2023) and tropical forests (Rozendaal & Chazdon, 2015). The VDMs herein, which are capable of simulating self‐thinning, are, in principle, able to account for these mortality dynamics and thus provide a crucial connection between forest recovery dynamics, the evolution of deadwood stocks, and hence forest soil dynamics. However, the large deviations in the timing of the different phases simulated by the models highlight the need for observational benchmarks that clearly differentiate between different forest phases in recovery. With forests in flux, and an increasing fraction of the landscape being in such a state (Shevliakova *et al*., 2009; Pugh *et al*., 2019), accurate representation and calibration of these regeneration and regrowth phases are clearly of high importance.

Overall, while we think that the novel qualitative benchmark (Table 2) used for the forest recovery‐phase alignment is a cheap, pragmatic way of monitoring model‐useful demographic transitions quickly. Nevertheless, the variables (e.g. CA, *n*stems, %C_veg_) and definitions will need refinement in the future. For example, the definition and monitoring of the closed‐canopy self‐thinning phase may need to accommodate the PPA multi‐storey canopy organisation approach more appropriately, as there, understory can form and self‐thinning is not immediately initiated once the canopy is closed. We faced challenges when attempting to balance ecological realism with model inclusivity. We view this forest phase framework as an important first attempt at aligning models within our community and with observed ecological processes, and are committed to improving these benchmarks in our community.

The self‐thinning rule (Yoda *et al*., 1963) is an ecological principle fundamental to understanding plant population dynamics and resource allocation. VDMs were able to replicate the self‐thinning slope, demonstrating their capability to simulate key ecological processes, such as intertree competition for light based on size. However, the models varied substantially in the number of years they spent in self‐thinning. This variation is likely determined by (a) the combination of growth rate and allometric assumptions that determine how quickly a recovering stand enters self‐thinning and (b) the point at which other causes of mortality start to kill off the dominant trees. The general trend towards fewer years of thinning at BCI across models possibly reflects the higher growth rates at that site. As assumptions on canopy light environments impact the individual cohort's productivity, the differences in how VDMs treat canopy layers and light partitioning in the vertical canopy profile highlight an opportunity for future in‐depth comparisons, particularly in the context of self‐thinning and succession, but also coexistence.

The self‐thinning period (time period a model spent in self‐thinning) can also be influenced by the method of selecting self‐thinning duration, which was kept as objective as possible, but varied depending on the model (see Fig. S11). We note that while the self‐thinning period selected using selection Methods 1–4 was independently confirmed by the modeller in the forest recovery‐phase analysis, it does not always align (Table S11). Self‐thinning mortality in the models is conceptualised through carbon starvation mortality (EDv3, FATES, BiomeE, BiomeEP and SEIB‐DGVM), growth‐efficiency mortality (LPJ‐GUESS and SEIB‐DGVM) or explicit rules based on biomass–stem number relationships (ORCHIDEE, crowding mortality in CABLE‐POP). Specifically, for ORCHIDEE, the biomass–stem number relationship is the main mechanism for triggering resource competition mortality more generally and therefore differs from the strict definition of self‐thinning for even‐aged stands in this work. ORCHIDEE's biomass–stem number relationship emerges well from the output but is a prescribed feature and cannot be used to determine the (even‐sized) self‐thinning period length. JULES‐RED's self‐thinning slope emerges as a combination of metabolic scaling theory, mortality and canopy area limiting seedling establishment (Argles *et al*., 2020). Realistically replicating this well‐established ecological behaviour (Yoda *et al*., 1963; Enquist *et al*., 1998) is crucial for more realistically simulating forest recovery and ecosystem carbon residence times.

### Benchmarking mature forest

Simulations of mature forest carbon and structure largely align with observations across all three biome sites (Fig. 1). This is consistent with previous studies which have shown that DGVMs, even when nondemographic, are generally able to simulate realistic levels of biomass (Forkel *et al*., 2016; Pugh *et al*., 2020), although regional biases exist (Forkel *et al*., 2019).

Yet, analysis of mature forest growth and mortality rates (Fig. 5) shows that similar biomass pools across models result from compensatory interactions between gross growth fluxes and turnover times, which also diverge significantly between models: Most VDMs overestimated growth rates (six to seven models above the observed median, site‐dependent) and, consistent with Pugh *et al*. (2020), underestimated carbon turnover times (six to eight models below the observed median turnover, though often within the observed variability). This accelerated cycling of carbon through wood in the models, compared with the real world, is potentially a result of not properly representing environmental constraints on woody growth (Delpierre *et al*., 2016; Friend *et al*., 2019), assumptions on allometry and allocation (Wolf *et al*., 2011; Massoud *et al*., 2019), or other poorly constrained demands on reproductive processes or root exudates, the latter found to make up 9–14% of global gross primary productivity (Chari *et al*., 2024).

The general character of the forest structure (carbon and stem number distribution across size classes) is captured by the model ensemble with some individual model scatter (Fig. 4), although the log scale may obscure discrepancies. Some of the differences likely result from a lack of information on site history, as forest structure at the plot scale is strongly dependent on the management and gap–disturbance history at the observational sites. Here, the absence of forest management more than 200 yr ago cannot be guaranteed in the observations at FIN. If some selective logging did occur in the past, this could explain why we found the presence of large trees in the models but not in the observations. Averaging across larger areas typically dilutes the impact of any given historical disturbance on the overall statistics. However, it is possible that the observed total areas at FIN (56 plots, 8.88 ha) and BIA (5 plots, 44 ha) are too small relative to the typical size and frequency of disturbances to provide a general characterisation of the ecosystem. This is difficult to assess as the disturbance rates that would exist in the absence of management in European forests are not well known (e.g. Pugh *et al*., 2024).

Notably, most models were able to simulate large trees (with the exception of BiomeE, BiomeEP and FATES in FIN and BIA), which have a very large influence on the total biomass simulated. Given the lack of available observations on the dynamic rates of large trees, particularly for mortality (Coomes & Allen, 2007; Gora & Esquivel‐Muelbert, 2021), there is a particularly high potential for error in large tree mortality rates in VDMs.

#### Summary of results and outlook

Overall, the benchmarking highlights that VDMs generally replicate carbon pools and forest structure in mature forests reasonably well. They also replicate (often emergent) processes, such as self‐thinning and demonstrate plausible interactions between forest structural variables and carbon fluxes during regrowth phases. However, there are uncertainties in the duration of different stand development phases and divergence of growth rates and turnover time from observations. In this study, growth and mortality rate data were withheld from the modellers; these rates, if properly constrained, could propagate into addressing other issues, such as the length of the self‐thinning period and the rate of C_cwood,AG_ recovery. We suggest that focusing on leveraging growth and mortality rate data to calibrate the models is a ‘low‐hanging fruit’ that is likely to provide major improvements in model performance.

### Assessment of available Benchmarks

In the benchmarks used in this study, there is variation in data suitability across variables from different biomes and stages of forest development, which consequently impacts the assessment of model performance (Seiler *et al*., 2022). Similarly, model output outside the observed variability can result from a misalignment between the scale of the model and the scale at which observations were made (Fritsch *et al*., 2020; Knapp *et al*., 2022). Therefore, the quality of the benchmarks themselves and the scales at which they are compared must be treated with care. Below, we further address several aspects to which attention needs to be paid in the formulation of such benchmarks, both in terms of sampling and in terms of modelling. Generally, the benchmark data have to be collected, aggregated, down‐ or upscaled, and documented such that they can be meaningfully used for process validation.

Similarly, models have a range of different conceptual scale assumptions, meaning that individual VDMs or groups of VDMs each may need their own tailored benchmarks. At the same time, there are pragmatic trade‐offs to be made between the time invested in complicated simulation setups and output procedures and realistically running and obtaining output for benchmarking efforts like this. Benchmarks need to be fit for purpose, but not necessarily perfect.

#### Trajectories through time

The chronosequence data used for regrowth benchmarking in this study were only able to provide a very loose constraint on stand regrowth rates. Continental (BCI + BIA) to regional (FIN) scale data were used to construct chronosequences (Fig. S2), which integrates not only over stand regrowth stages but also over substantial environmental variability. Datasets for the temporal benchmarking of forest carbon pools are relatively numerous, but are normally derived from chronosequence data from multiple sites, which do not allow for an accurate determination of the time to recovery (Walker *et al*., 2010). The effects of environmental variability could be mitigated against by simulating each plot individually using site‐specific environmental driving data. This was, however, not practical to realise across many models in this study. Our sites' mean annual temperatures are, however, generally representative of the regrowth benchmark population, with BIA and BCI within typical ranges and FIN slightly above the benchmark median, suggesting above‐median regrowth dynamics (Methods S8; Fig. S20).

An alternative to chronosequences is comparisons made against longitudinal data from individual sites. Although we are aware of relatively few such plot‐level datasets and they rarely extend beyond a few decades of observations, there are enough to create a benchmark at least in the tropics (Longworth *et al*., 2014; Mesquita *et al*., 2015; Rozendaal *et al*., 2017); 2ndFOR collaborative research network, Tropical Managed Forest Observatory. These datasets come with the substantial benefit that they can often also provide growth and mortality rates, as well as overall net biomass increment, further constraining dynamics. Satellite‐derived chronosequences (Heinrich *et al*., 2021) can support but not replace these plot‐based benchmarks because of the importance of resolving growth and mortality dynamics and composition change.

#### Spatial scale

The range in the mature forest aboveground woody carbon content at BIA (Fig. 1b) is derived from a limited number of plots (*n* = 5, with a total combined size of 44 ha), which are area‐based averages of aboveground woody carbon content. The variability in biomass is a function of the area over which the individual data points are summed, with larger areas dampening the variability resulting from what are essentially stochastic processes. If model results representing one scale are compared with observational results derived from another scale, then the comparison is likely to be flawed. This will particularly be the case when variances (Knapp *et al*., 2022), as opposed to the means, are compared. For future benchmarks, the scaling of observed forest variables will be both useful and necessary for making more appropriate use of small‐scale sites, but transfer functions (Knapp *et al*., 2022) at a meaningful sampling frequency level must be derived for more ecosystems than the tropics. Ideally, the combination of spatial scale and the time period observed (Urban *et al*., 1987) needs to be large enough to provide a representative sample of the dynamics. For example, if a large fraction of the overall mortality derives from relatively rare events that kill many trees at the same location, such as blowdowns, then the observations and model outputs need to have sufficient spatial and temporal extent to characterise the return periods of these events. Because mortality regimes are regionally specific, the necessary temporal and spatial extent will therefore vary by forest region and vegetation type (Reilly *et al*., 2016).

### Suggestions for additional benchmarks

While this study has highlighted that process uncertainty is highest during establishment and regrowth, we cannot provide enough data constraints to address these deficiencies with confidence. To better validate demographic model process behaviour requires site‐level data that are higher resolved (in time), diverse (in the variables measured, covering carbon pools and fluxes and structure), and at a process‐relevant scale to adequately validate the models. We have identified a set of observations (Table 3) which would be desirable for process validation and present the state of data availability (to our knowledge).

**Table 3:** Benchmarking variables useful for demographic model benchmarking.

Forest state/time period	Benchmark (1 = priority; 2 = desirable)	Process/state benchmarked	Benchmark variable (units)	Status	Requirements	Example studies/projects found (Ecosystem)	Process to be constrained
Recruitment	1	Recruitment	Seedling survival (%survival) seedling allometry: dbh (cm), height distribution (m)	Available	Multiple environmental gradients and ecosystems, Land‐use history	EuFoRia (Díaz‐Yáñez *et al*., 2024) ForestGEO, https://hubbardbrook.org/data‐catalog/	To determine trajectory of the ecosystem recovery speed, necessary, especially with increased disturbance/land‐use change and at policy‐relevant timescale.
Regrowth	2	Structure	Carbon content per size classes (kgC m^−2^)	Exists (as growth increment rates exist) but not processed suitably	Multiple environmental gradients and ecosystems, Land‐use history	Chave *et al*. (2020)	To constraint competitive interactions as forest regrows. Necessary to get correct, as it determines forest resilience, too
Regrowth	1	Dynamics	Growth increment rates (kgC m^−2^ yr^−1^)	Available: Few plot‐level studies exist. Exists: Many satellite products exist, but the observations would need postprocessing from Benchmark ‘Regrowth Pool Forest carbon content’	10–20 yr intervals, finer at the beginning, depending on ecosystem productivity (e.g. Chave *et al*., 2020), different units need conversion. Multiple environmental gradients and ecosystems, Land‐use history. Need to standardise: Different definitions of ‘biomass’ for growth rates. Some used volume, others biomass	Plot level: Pretzsch *et al*. (2023) (temperate forests), Chave *et al*. (2020) (tropics, size class), Rozendaal & Chazdon (2015) (tropics), Chazdon (2021) (tropics)	To assess recovery sensitivity to climate and resource availability, as growth increment rates integrate forest structure and competition, they can to some extent replace these datasets. To constrain physiological processes related to woody biomass increment, using multi‐site data
Regrowth	1	Structure	Number of stems per size class (n ha^−1^)	Exists	Described in the previous section	Mesquita *et al*. (2015) (Total number of stems only)	To constrain productivity, recruitment, competition and allometric assumptions
Regrowth	2	Trait dominance	Progression of dominant traits over time	Exists, numerous ecological observational studies.	Described in the previous section	Mesquita *et al*. (2015); Rozendaal *et al*. (2017)	This information could be leveraged to benchmark against number or diversity of functional traits present during the course of recovery, and how that relates to simulated forest resilience. To constrain the presence and (relative) number of early and late‐successional traits, also in different environmental conditions
Regrowth	1	Pool	Forest carbon content (kgC m^−2^)	Available	Limited time (Nonchronosequene) series length available < 30 yr stand age	(1) 2ndFor (2) Cook‐Patton *et al*. (2020), (3) Palviainen *et al*. (2020), this study. Need clear method/equation of how carbon content was estimated. Need age of the forest. (4) Satellite data: Heinrich *et al*. (2021)	A high‐level variable to constrain forest recovery speed.
Regrowth	1	Dynamics	Mortality rates (kgC m^−2^ yr^−1^, %/yr)	Available	10–20 yr intervals, finer at the beginning, depending on ecosystem productivity Desirable: by size class	Rozendaal & Chazdon (2015); Chazdon (2021)	To assess recovery sensitivity to climate and resource availability, as mortality rates integrate forest structure and competition, they can to some extent replace these datasets
Regrowth	1	Dynamics	Open‐canopy phase – grass competition – duration (yr)	Required: Ecological survey design and data collection. Exists: satellite data	Created from a multitude of variables thought to act together to classify phases, qualitative combined use of: %Cveg, *n*stems, CA	Ecological surveys and satellite data for example Mandl *et al*. (2024) (satellite data for Europe)	All four phases are used to constrain carbon and ecological dynamics (growth rates, self‐thinning rates) and successional trajectories, where simulated, and using multiple variables together, even if qualitatively, will get a link to different types of resilience. Vital data in times of more frequent disturbances
Regrowth	1	Dynamics	Open‐canopy phase – growth duration (yr)	Available	Qualitative combined use of *n*stems, %Cveg, cmort_rate_ (% yr^−1^), CA	Described in the previous section	Described in the previous section
Regrowth	1	Dynamics	Closed‐canopy self‐thinning duration (yr)	Available	Qualitative combined use of cmort_rate_ (% yr^−1^), *n*stems CA	Described in the previous section	Described in the previous section
Regrowth	1	Dynamics	Closed‐canopy self‐thinning duration (yr)	Exists to some extent in the tropics, but not postprocessed	Quantitative: postprocessed from mass and number density and growth rates.	Chazdon (2021)	To benchmark mortality processes and rates. A critical duration of carbon loss which cannot be explicitly simulated by nondemographic models
Regrowth	1	Dynamics	Closed‐canopy late‐successional phase (yr)	Required: Ecological survey design and data collection. Exists: satellite data	Qualitative combined use of %Cveg, cmort_rate_ (% yr^−1^)	See qualitative phases above	See qualitative phases above
Mature forest	1	Pool, structure	Carbon content, per size classes (kgC m^−2^)	Available	Need clear methods+ allometric equations for how carbon content was estimated	This study; NFI‐data; https://www.remoteforests.org/ (Bin *et al*., 2012)	A high‐level variable to constrain forest carbon boundary, but most meaningful when used in context with forest structure (cwood_size *n*stem_size)
Mature forest	2	Dynamics	Growth rates (kgC m^−2^ yr^−1^)	Available	Ideally also by dbh‐size class	This study, https://forestplots.net/ NFI‐data example: https://hubbardbrook.org/data‐catalog/	To constrain biomass dynamics, age‐related productivity changes, ecosystem stability in context with the below mortality rates
Mature forest	1	Dynamics	Mortality rates (kgC m^−2^ yr^−1^)	Available	Ideally also by dbh‐size class	This study, Bennett *et al*. (2015); Piponiot *et al*. (2022)	To constrain age‐related and competition‐related mortality and overall forest turnover.
Mature forest	2	Trait dominance, coexistence	Dominant traits	Available	Stand age information or metadata information on the forest having reached closed‐canopy late‐successional phase (further above in this table)	Kattge *et al*. (2020); Rüger *et al*. (2020)	To constrain life‐trait and successional trait parameterisation
Regrowth /recovery after different disturbance agents	1	Combination of all	Collection of many of the above	Available	Different types, scales and severities of disturbance agents will impact forest structure and subsequent dynamics differently, temperate forest only	Defoliation (FoRTE experiment; Atkins *et al*., 2023) Girdling (accelerated succession experiment; Gough *et al*., 2021) Fire/harvest chronosequences (Gough *et al*., 2007)	With increasing disturbance severity and frequency, these disturbance manipulation experiments and datasets are unique, and give us insights into DVM's realism in recovery behaviour. Useful for comparing against a variety of variables, structural, fluxes and traits
Responses to Environmental change, environmental limits, canopy status.	1	Dynamics	Normalised ring width index	Available	Some postprocessing and method‐development on how it can be compared against model output will be necessary.	International Tree‐Ring Database: https://www.frames.gov/catalog/56 Dendroecological network: https://www.uvm.edu/femc/dendro African Tree Ring network: https://pastglobalchanges.org/science/wg/atrn/intro Tropical tree‐ring network: https://tropicaltreeringnetwork.org	To benchmark demographic models' physiological growth response to environmental change. Potentially also useful to distinguish canopy and subcanopy growth responses to environmental change
Actual forest state and dynamics	1	Combination of all	Collection of many of the above	Exists	Summaries at a grid level for comparison with large‐scale VDM simulations. Requires that the same of observations is representative of forest in that grid cell	National Forest Inventory plots. Combinations of research plots and systematic forest age maps	The overall ability of the model to capture the actual forest state and dynamics, combining across all processes and input datasets is a fundamental characteristic to assess

Benchmarks can be categorised as necessary or desirable/optional for demographic models. The benchmarks can guide future development and can therefore further be divided into (1) priority for this generation of VDMs, and (2) desirable to further develop VDMs. Status column specifications: Exists: studies have been done, but time investment into collecting and cataloguing the studies and collective formatting is required. Available: where networks have been creating a common data standard, which modellers could benefit from, or studies have collected multiple studies' data. Some of these networks are not openly accessible and data agreements are needed, this is not distinguished here. Required: In need of collection.

#### Initial establishment conditions

Benchmarks on seedling survival and regeneration (increase in a size class vs death) rates are useful approaches to evaluating initial conditions of the models, one of the processes that would benefit from further developments. Díaz‐Yáñez *et al*. (2024) conducted a comprehensive intercomparison study on modelled forest recovery, revealing that models which overestimate recruitment compared with observations tend to show the highest mortality in small size classes, exceeding values seen in data. This suggests compensatory mechanisms at play, and while eventually stabilising, this would lead to artificial simulated turnover during the early stages of forest recovery. However, while short‐term dynamics are critical, the initial seedling establishment conditions also have an impact on long‐term processes. Fisher *et al*. (2010) found that, among five demography‐related parameters, sapling mortality had the greatest impact on community structure and ecosystem properties, highlighting the long‐term influence of seedling survival.

Recruitment data along an environmental gradient across Europe are now available through the EuFoRia network (Díaz‐Yáñez *et al*., 2024), but such data are typically scattered across individual studies and often not compiled for benchmarking. Compilations of observations on seedling mortality, regrowth and small tree allometry (particularly crown area and bole height) across diverse ecosystems are urgently needed to benchmark initial conditions and their impact on later stages of forest recovery. This data would not only aid benchmarking, it would also be highly valuable to inform process development of seedling survival, especially at larger scales.

#### Forest recovery

In the absence of regrowth observations for this study, we generated qualitative benchmarks (‘forest recovery phases’) containing a collection of plausibility criteria to reflect forest recovery‐phase behaviour. While this may be a useful method to overcome data scarcity, it would be preferable to enhance these benchmarks with observations on ecosystem‐specific boundary‐values between the phases (if they exist). Two options for improving both our definitions of the forest phase benchmarks and reducing data scarcity are to engage satellite sequences and on‐the‐ground ecological field surveys. These two approaches could be pursued independently or, ideally, in tandem. Improved satellite spectral analysis on recovering forests (Mandl *et al*., 2024) can provide information on canopy openness, and quick questionnaires for local ecologists can provide further information to help with determining a given forest's phase.

#### Forest structure

While forest structure data from inventory plots with stand age exists, it is often not organised to specifically capture early recovery dynamics. Efforts to collate and structure such data would greatly enhance our ability to benchmark and constrain recovery‐phase model processes with accuracy. Regrowth is especially crucial to resolve correctly in a time when many forests are in disequilibrium and our understanding of the future capacity of these young forests to gain and retain carbon needs improving.

Organising other benchmark variables into size classes could allow for deducing or constraining general mortality behaviour patterns across ecosystems (e.g. Piponiot *et al*., 2022) or, for example, size‐specific mortality reactions to water availability (Bennett *et al*., 2015). Such data are altogether absent (with some few exceptions, see Table 3) for many types of forests.

Self‐thinning lines integrate forest structure by showing the interplay between mass and number density within a forest and are therefore useful observations for constraining demographic models. Beyond self‐thinning, general forest structure features, such as number and mass–density relationships exist between ecosystems, with the variability linked in part to rainfall seasonality (Yu *et al*., 2024). These findings suggest that these relationships can be used to constrain not only competition for light but also for water. Self‐thinning lines or older‐growth mass–density relationships would be valuable tools for model verification. Longitudinal self‐thinning (while also keeping track of other mortality causes) throughout the whole forest recovery trajectory would be a useful dataset for model constraint, as it would implicitly integrate forest structure observations with forest growth rates. Active remote sensing also offers new opportunities to benchmark forest structure in VDMs (Drake *et al*., 2002; Dubayah *et al*., 2010, 2020). Airborne lidar data on vegetation 3‐D structure have been powerfully used together with the Ecosystem Demography model in a range of studies from local to regional scales (Thomas *et al*., 2008; Dubayah *et al*., 2010; Antonarakis *et al*., 2011; Hurtt *et al*., 2016, 2019, 2024; Ma *et al*., 2021). Recently, these same benefits have been demonstrated globally using spaceborne lidar (Dubayah *et al*., 2022; Ma *et al*., 2022, 2023).

#### Growth interannual variability

Tree‐ring data, though abundant, has not yet been systematically used to benchmark VDM woody biomass growth responses to environmental conditions, with few site‐specific, single‐model exceptions (e.g. Jeong *et al*., 2021; Xu *et al*., 2024). Tree‐ring data reflect both individual‐tree growth and collective biomass changes across plots in response to the environment. Forest structure and carbon result from the balance between biomass loss through mortality and accumulation through growth, with the latter being both a physiological response and demographic response that serves as a key driver in ecosystem dynamics. While reusing samples that have been taken for climate reconstruction is feasible, it requires working around their sample biases (Jeong *et al*., 2021), and networks are emerging that target the use of tree rings in ecological studies (DendroEcological Network (DEN), Tropical tree‐ring network (TTR), African Tree Ring Network (ATRN), REMOTE forests).

#### Other benchmarks

Besides structural information, species data, while not directly comparable to large‐scale simulations and PFT abstraction in global VDMs (Table 3, Regrowth – ‘Trait dominance’), are also required to determine whether models can correctly represent succession dynamics, something this study has not covered. Achieving successional dynamics and coexistence in VDMs is still challenging and would be a valuable next step in benchmarking. Individual studies on species abundance (which could be abstracted through PFT or functional trait abundance in VDMs) and resilience on abandoned farmland exist (e.g. in the Tropics; Mesquita *et al*., 2015). However, knowledge of typical successional dynamics, including the timescales on which forests typically transition from one species to another, is often well established regionally, at least for northern‐hemisphere temperate and boreal forests (Van Cleve & Viereck, 1981; Shorohova *et al*., 2009; Taylor *et al*., 2020). This could be assembled through the literature review or surveys of local experts.

In addition to land‐use and forest management, forest structure is also influenced by a diversity of disturbance events (Table 3, ‘Regrowth/recovery after different disturbance agents’), expected to increase in frequency and severity in the future (Seidl *et al*., 2017; McDowell *et al*., 2020), where different agents (bark beetle, wind, fire) leave behind different forest structures, and therefore recovery trajectories and levels of resilience. Large‐scale disturbance manipulation studies, with a comprehensive suite of benchmark variables measured, would provide an additional valuable benchmark for this (e.g. FoRTE project; Atkins *et al*., 2023).

#### Benchmarking against the overall state of global forests

The combination of all variables (biomass, forest structure, mortality and harvest rates, growth rates, as realised with all legacies, in response to environmental change) into a coherent constraining system should be the ultimate goal of a benchmarking effort for VDMs.

High‐quality constraints of forest dynamics and structure require that the benchmark suite not only isolates behaviour during particular phases of stand development and management, but also covers the actual state of the forest. This requires benchmarking data that are representative of the current state of the forest. Available forest plot data broadly fall into two categories: research plots for which there is often fairly detailed information on management or its absence, and National Forest Inventory (NFI) plots for which management information is often very limited or absent.

While the limited meta‐information on NFI plots makes them challenging to use to isolate particular aspects of forest behaviour, their systematic sampling across the forest makes them very well suited to benchmark the actual forest structure and dynamics that have emerged from the combination of ecology, management and land‐use history. Making use of this constraint, however, requires that VDMs can simulate not only demography but also land‐use change and forest management, and is naturally not only a test of VDMs themselves but also the input datasets used. Research plots are well suited to isolate behaviour, although those plots which are suitable to derive dynamics tend to focus on mature or old‐growth forests. It is crucial that observational data are appropriately selected to benchmark the feature of interest. To gain the spatial representativeness needed to benchmark VDMs effectively, large compilations of plots are needed. These are challenging to assemble both for reasons of diverse data ownership and also because of the diverse sampling protocols used. Emerging efforts to analyse forest dynamics at continental/biome scales are making benchmark assembly increasingly feasible (Hubau *et al*., 2020; Astigarraga *et al*., 2024; Bialic‐Murphy *et al*., 2024; EuForIA network). To make maximal leverage of these efforts to improve VDMs, modellers must be closely involved in their analysis, requiring the development of close collaborations across disciplines.

Ultimately, the choice of benchmarks depends on the processes studied. However, establishing a standardised minimum set of benchmarks to validate fundamental demographic and ecological principles, as well as carbon stocks and fluxes, would provide a crucial baseline for ensuring the robustness and credibility of VDM predictions. Implementing such benchmarks as routine checks should become standard practice in the field. Standardisation of benchmark definitions (including metadata, units and data‐formatting rules) is a work in progress (see Table S6 for observations), and needs to be paired conceptually and technically with the correct output (table 3.1 in Notes S1 for model output), and requires further refinement in the future.

### Call for benchmarking and implications

We have compared the performance, assumptions and outputs of nine VDMs. The models differ in their fundamental assumptions on how forests are conceptualised both in time and vertical space. Yet, all replicate observed forest dynamics and structure reasonably well, both during regrowth and mature forest stages at three sites, each representing a distinct biome. However, we find that development attention needs to be focused on (1) disturbance regeneration and recovery/forest regrowth, where models diverge the most, and (2) calibration to improve woody growth and mortality rates to determine turnover time. Finally, the performance of the models against a limited set of observations showcases the need for more in‐depth benchmarking studies, standardised model evaluation, routine demographic carbon budget checks and harmonisation of definitions.

Models differed the most in the initial conditions and subsequent years, but since all models behave ecologically plausibly along a sequence of forest recovery phases, we believe that, once calibrated, these models have the structural capabilities to represent forest dynamics throughout the full regrowth trajectory directly from postestablishment through to old growth. By resolving forest structure, growth and mortality at the individual or cohort‐level, VDMs typically introduce more parameters compared with nondemographic models, but because those parameters have direct ‘points of contact’ with real‐world observations of individual trees and stands, there is substantial potential to tightly constrain their ranges. Our expectation is that with process representation at this fundamental level of process detail adequately constrained/calibrated, we can increase the realism in the simulated future vegetation dynamics. This advancement could help contribute to decreasing the persistent problem of vegetation carbon stock divergence among models in simulations under future climate change (Arora *et al*., 2013, 2020; Friedlingstein *et al*., 2014; Lovenduski & Bonan, 2017), provide assessments of the potential and permanence of nature‐based solutions (e.g. Cook‐Patton *et al*., 2020), as well as our ability to resolve the current forest carbon sink (O'Sullivan *et al*., 2024).

We proposed a list of benchmarking variables specific to demographic models that are necessary or desirable to comprehensively challenge our demographic models at all stages of forest development (Table 3). Future challenges will be to collate and format existing data to the processes and scales VDMs are designed to represent. Comprehensive datasets covering forest dynamics, pools and structure at all stages of forest development and biomes are needed. It is much easier to call for more data collection than it is to carry it out. We have identified some cases where additional collection would be valuable. But for the most part, suitable observations exist but are not always available to model developers, whether for reasons of being collated and analysed in the form needed or because of data ownership. Close collaboration between groups making the observations and those developing VDMs will be required to bridge this gap.

Technical and conceptual modelling and postprocessing challenges pertained to the output variables themselves. During the course of the initiative, evolving definitions and outputs of variables and a need for defining a ‘demographic’ woody carbon balance (Methods S2) underscore the need for standards for variable definitions, formats and model testing. Enhanced formatting is likewise required if the benchmarking variables become components of land‐surface suites, such as iLAMB. For example, frameworks and outputs will need to be modified/extended to accommodate the specific structure and dimensions of demographic data (e.g. the ‘self‐thinning’ space). Our simulation protocol (Notes S1) can provide a basis for further efforts in this space.

This study is a first step towards a currently lacking demographic model benchmarking suite. Even a subset of these variables from our benchmarking suite (Table 3) will be an indispensable tool to evaluate and provide credibility to these increasingly policy‐relevant models at the level of detail relevant for policy makers and for the simulation of future vegetation responses to climate and land‐use change. Our combined efforts of the demographic modelling community in this study are a first step towards this. With improved calibration and postrecovery initialisation, VDMs are ready to support realistic assessments of afforestation and reforestation efforts, enhancing the credibility of policy‐relevant short‐term and long‐term forest carbon and structure projections.

## Competing interests

None declared.

## Author Contributions

TAMP and AHES conceived the initiative and initial simulation protocol and coordinated the intercomparison work. RAF, CDK, PMC, JN, APKA, SL, AEM, SO, JK, SS, DZ and MGDK refined the simulation protocol. JK (CABLE‐POP), Lei Ma and GCH (EDv3), AHES and SO (LPJ‐GUESS), Laura Marqués and BS (BiomeEP), EW (BiomeE), JN (FATES), APKA, PMC and JRM (JULES‐RED), HS (SEIB‐DGVM), SL and GM (ORCHIDEE) done model runs and output postprocessing into demographic benchmarking (‘D‐BEN’) format. KP, DZ, MP, AL, BB and AEM performed the observational data preprocessing and advice. Laura Marqués performed the analysis code review. AHES coordinated the simulation output analysis and wrote the first draft. All authors commented on the manuscript and accepted the final version.

## Disclaimer

The New Phytologist Foundation remains neutral with regard to jurisdictional claims in maps and in any institutional affiliations.

## Supporting information


**Fig. S1** Demographic (woody) carbon budget test results, and visualisation of the temporal trajectory of the outputted carbon pool variable C_wood_, and the flux‐derived carbon pool.
**Fig. S2** Observed chronosequence regrowth and mature forest dynamics benchmarking data.
**Fig. S3** Benchmarking data of stand structure, number of stems, and woody biomass by size class.
**Fig. S4** Distribution of data used for this study.
**Fig. S5** Example of the plots that can visualise the self‐thinning period and length.
**Fig. S6** An example of using Method 1.
**Fig. S7** An example of using Method 2.
**Fig. S8** An example of using Method 3.
**Fig. S9** An example of using Method 4.
**Fig. S10** Two‐step process for manual self‐thinning selection Method 4.
**Fig. S11** Self‐thinning method selection for each individual model and site.
**Fig. S12** Mean equilibrium biomass plotted against mean annual regrowth rate over the first 50 yr postdisturbance.
**Fig. S13** Woody mortality rates at FIN, for all models, smoothed using a 30‐yr running mean.
**Fig. S14** Woody mortality rates at BIA, for all models, for all models, smoothed using a 30‐yr running mean.
**Fig. S15** Woody mortality rates at BCI, for all models, for all models, smoothed using a 30‐yr running mean.
**Fig. S16** Example of a mortality rate trajectory within the forest recovery phases.
**Fig. S17** Stand structure from LPJ‐GUESS run output at simulation year 450 with no patch‐destroying disturbance, and a 100‐yr disturbance interval (default) turned on.
**Fig. S18** Succession patterns in models that simulate between‐PFT competition as part of demographic dynamics.
**Fig. S19** Growth rates at Bialowieza for LPJ‐GUESS and BiomeE, showcasing the impact of PFT succession on the growth rate during recovery (30‐yr‐smoothed).
**Fig. S20** Frequency distribution of mean temperature for the years 1900–2023 for all sites for which regrowth datapoints exist, and frequency distribution of mean temperature for years 1991–2020, for the three simulation sites.
**Methods S1** Model descriptions and model‐specific setups for this study.
**Methods S2** Demographic carbon balance.
**Methods S3** Observational data.
**Methods S4** Determination of the self‐thinning period and slope.
**Methods S5** Forest recovery phases and phase alignment.
**Methods S6** Stand structure benchmarks.
**Methods S7** ‘Naïve’ – unaligned regrowth rate analysis.
**Methods S8** Mean temperature analysis for regrowth sites.
**Notes S1** Simulation protocol used to perform the benchmarking simulations.
**Notes S2** Forest phase alignment, additional variables used for evidence for identifying the forest Phases.
**Notes S3** Stand structure benchmark per model and site, with the observational data ranges reported in Table S6.
**Notes S4** Regrowth benchmark per model and site, with the observational data ranges reported in Table S6.
**Table S1** Individual model‐PFTs mapped onto site‐specific species (plant categories for BCI).
**Table S2** Description of model‐specific modes of biomass reductions that was invoked to enable the complete forest removal after 30 yr of simulation.
**Table S3** Parameters adjusted from default parameter set to obtain P0 output.
**Table S4** Mapping of model‐output variables to D‐BEN variables, where these deviate from the ‘D‐BEN’ definition (table S3.1 in Notes S1).
**Table S5** Upper and lower simulation year that make up the period from which each model is considered in equilibrium.
**Table S6** Benchmark data overview in terms of range, type and justification for the method to derive observed ranges, alongside with some comments/observations on the behaviour of the data, and filtering criteria.
**Table S7** Self‐thinning selection method per model and site and resulting duration and self‐thinning slope identification Methods 1–4 are briefly explained.
**Table S8** Detailed forest phase classification.
**Table S9** Forest Recovery Phase Classification. Initial phase and self‐thinning timing.
**Table S10** Forest Carbon and dynamics variables summarised across the first 30 yr of regrowth and equilibrium.
**Table S11** Independent identification of self‐thinning phase onset from two methods, self‐thinning onset determination and forest phase determination.
Please note: Wiley is not responsible for the content or functionality of any Supporting Information supplied by the authors. Any queries (other than missing material) should be directed to the *New Phytologist* Central Office.

## Data Availability

All analysis scripts, benchmarking data and model output data specific to this manuscript are deposited on 10.5281/zenodo.14415143. Model versions and availability: LPJ‐GUESS: v.4.1, branch mechanistic_mortality_dben r11972 https://zenodo.org/records/15837787 and postprocessing from https://github.com/teatree1212/DBEN_postprocess_LPJGUESStoDBEN, also deposited on https://zenodo.org/records/15837636. ORCHIDEE: This study made use of ORCHIDEE r8696 10.5281/zenodo.15805716. EDv3.0 is from https://zenodo.org/records/6901510. CABLE‐POP (rev 9526): 10.5281/zenodo.17050747. BiomeEP: model version from https://github.com/lauramarques/rsofun; model runs and postprocessing scripts from https://github.com/geco‐bern/DBEN. FATES: model version from https://github.com/JessicaNeedham/fates/tree/jfn‐dben‐api35‐seedhist and postprocessing scripts: https://github.com/JessicaNeedham/dben‐archive. BiomeE: https://github.com/wengensheng/BiomeESS. SEIB‐DGVM: 3.10 is from https://seib‐dgvm.com/en/. JULES‐RED: both the model branch, r24142_add_red_sci_vn1.1, and the rose suite used in the simulation and postprocessing, u‐cq124, are available from the Met Office Science Repository Service: access can be applied for via the link https://jules-lsm.github.io/access_req/JULES_access.html .
