# Supplementary material for: Demography, dynamics and data: building confidence for simulating changes in the world's forests
Source: New Phytol. 2025 Oct 23;248(6):2722–49. doi: 10.1111/nph.70643 (PMC12630465; doi:10.1111/nph.70643)
Supplement: Supplementary file 1 — Fig. S1 Demographic (woody) carbon budget test results, and visualisation of the temporal trajectory of the outputted carbon pool variable Cwood, and the flux‐derived carbon pool. Fig. S2 Observed chronosequence regrowth and mature forest dynamics benchmarking data. Fig. S3 Benchmarking data of stand structure, number of stems, and woody biomass by size class. Fig. S4 Distribution of data used for this study. Fig. S5 Example of the plots that can visualise the self‐thinning period and length. Fig. S6 An example of using Method 1. Fig. S7 An example of using Method 2. Fig. S8 An example of using Method 3. Fig. S9 An example of using Method 4. Fig. S10 Two‐step process for manual self‐thinning selection Method 4. Fig. S11 Self‐thinning method selection for each individual model and site. Fig. S12 Mean equilibrium biomass plotted against mean annual regrowth rate over the first 50 yr postdisturbance. Fig. S13 Woody mortality rates at FIN, for all models, smoothed using a 30‐yr running mean. Fig. S14 Woody mortality rates at BIA, for all models, for all models, smoothed using a 30‐yr running mean. Fig. S15 Woody mortality rates at BCI, for all models, for all models, smoothed using a 30‐yr running mean. Fig. S16 Example of a mortality rate trajectory within the forest recovery phases. Fig. S17 Stand structure from LPJ‐GUESS run output at simulation year 450 with no patch‐destroying disturbance, and a 100‐yr disturbance interval (default) turned on. Fig. S18 Succession patterns in models that simulate between‐PFT competition as part of demographic dynamics. Fig. S19 Growth rates at Bialowieza for LPJ‐GUESS and BiomeE, showcasing the impact of PFT succession on the growth rate during recovery (30‐yr‐smoothed). Fig. S20 Frequency distribution of mean temperature for the years 1900–2023 for all sites for which regrowth datapoints exist, and frequency distribution of mean temperature for years 1991–2020, for the three simulation sites. Methods S1 Model descriptions and model [file NPH-248-2722-s001.docx]

## *New Phytologist* Supporting Information

Article title: Demography, Dynamics and Data: Building Confidence for Simulating Changes in the World’s Forests

Authors: Annemarie H. Eckes-Shephard^1^ , Arthur P. K. Argles^2^ , Bogdan Brzeziecki^3^ , Peter M. Cox^4^ , Martin G. De Kauwe^5^ , Adriane Esquivel-Muelbert^6, 7^ , Rosie A. Fisher ^8^ , George C. Hurtt^9^, Jürgen Knauer^10,11^ , Charles D. Koven^12^ , Aleksi Lehtonen^13^ , Sebastiaan Luyssaert^14^ , Laura Marqués^15,16^ , Lei Ma^9^, Guillaume Marie ^17^ , Jonathan R. Moore ^4^ , Jessica F. Needham ^12,8^ , Stefan Olin ^1^ , Mikko Peltoniemi^13^ , Karl Piltz ^1^, Hisashi Sato^18^ , Stephen Sitch ^19^ , Benjamin D. Stocker ^15,16^ , Ensheng Weng^20^ , Daniel Zuleta ^21,22^, Thomas A. M. Pugh ^1, 6, 7^

^1^Department of Physical Geography and Ecosystem Science, Lund University, 223 62 Lund, Sweden; ^2^Met Office Hadley Centre, Exeter, Devon, EX1 PB, United Kingdom; ^3^Department of Silviculture, Institute of Forest Sciences, Warsaw University of Life Sciences, 02-776 Warsaw, Poland; ^4^Department of Mathematics and Statistics, Faculty of Environment, Science and Economy, University of Exeter, Exeter EX4 4QF, UK; ^5^School of Biological Sciences, University of Bristol, Bristol BS8 1TQ, UK; ^6^School of Geography, Earth and Environmental Sciences, University of Birmingham, Birmingham, B15 2TT, UK; ^7^Birmingham Institute of Forest Research, University of Birmingham, Birmingham, B15 2TT, UK; ^8^CICERO Center for International Climate Research, 0349 Oslo, Norway; ^9^Department of Geographical Sciences, University of Maryland, College Park, MD 20742, USA; ^10^Hawkesbury Institute for the Environment, Western Sydney University, Penrith, NSW 2751, Australia; ^11^School of Life Sciences, Faculty of Science, University of Technology Sydney, Ultimo, NSW 2007, Australia;^12^Climate and Ecosystem Sciences Division, Lawrence Berkeley National Laboratory, Berkeley, CA, 94720, USA; ^13^Natural Resources Institute Finland (Luke), FI-00790 Helsinki, Finland; ^14^Amsterdam Institute for Life and Environment, Vrije Universiteit Amsterdam, Amsterdam, 1081, the Netherlands; ^15^Institute of Geography, University of Bern, 3012 Bern, Switzerland; ^16^Oeschger Centre for Climate Change Research, University of Bern, 3012 Bern, Switzerland; ^17^Science Partners, 75010 Paris, France; ^18^**Research Institute for Global Change (RIGC), Japan Agency for Marine-Earth Science and Technology (JAMSTEC), 3173-25 Showamachi, Kanazawa-ku, Yokohama, 236-0001, JAPAN;**

^19^Department of Geography, Faculty of Environment, Science and Economy, University of Exeter, Exeter, EX4 4QF, UK; ^20^Center for Climate Systems Research, Columbia University and NASA Goddard Institute for Space Studies, New York, NY 10025, USA; ^21^Forest Global Earth Observatory, Smithsonian Tropical Research Institute, Washington, DC 20013-7012, USA; ^22^Department of Biological and Environmental Sciences, University of Gothenburg, 413 90 Gothenburg, Sweden;

Article acceptance date: 01 August 2025

The following Supporting Information is available for this article:

**Methods S1** Model descriptions and model-specific setups for this study

#### BiomeE

BiomeES is a standalone demographic vegetation model, derived from the demographic module of LM3-PPA (Weng *et al*., 2015). In this model, plants are represented as cohorts of similarly sized individuals arranged in canopy layers following the rules of the Perfect Plasticity Approximation (PPA) model (Strigul *et al.*, 2008). Plant traits define the parameters of physiological and demographic processes, reflecting strategies for competing for and utilising limited resources under specific environmental (climatic and edaphic) conditions. Demographic processes within the model create and remove cohorts and adjust the size and density of individuals within these cohorts. By explicitly describing cohort size, organisation, and composition throughout the simulation, the model effectively simulates competition for light and soil resources, community assembly, and vegetation structural dynamics (Weng *et al*., 2017, 2019)

#### BiomeEP

BiomeEP is a derivative of BiomeE (Weng *et al*., 2015, 2019), which contains an implementation of the P-model (Prentice *et al.*, 2014; Wang *et al.*, 2017; Stocker *et al.*, 2020) for predicting acclimated photosynthetic parameters, assimilation, and dark respiration rates as a function of the environment. BiomeEP allows for an explicit representation of cohorts of equally sized individuals and for a treatment mortality. A size-dependent mortality rate was specified for the upper-canopy layer, assuming the yearly mortality rate of trees to follow a power law relationship with the tree's diameter. An understory mortality rate was applied to the model setup, with higher mortality rates for the smaller and younger understory cohorts.

#### LPJ-GUESS

LPJ-GUESS (Lund-Potsdam-Jena General ecosystem simulator) is a an individual or cohort-based gap model (Smith *et al.*, 2001) that simulates various ecosystems, including forests, and represents demographic processes such as growth, establishment and mortality. Within-and between PFT competition occurs through cohorts of different sizes competing for light, and patch-scale water and nitrogen competition (Smith *et al.*, 2014). Patches represent samples across the landscape and variability between these patches is achieved through stochasticity in the establishment, various mortality processes, including patch-destroying disturbances. It can simulate fire (Rabin *et al.*, 2017). Recent extensions include a detailed forest management scheme (Lindeskog *et al.*, 2021).

It was used in its standard setup and parameterisations as from (Pugh *et al.*, 2019), with PFT-specific changes for BCI following (see Table 3). The number of patches was set to 500 and the simulations were run for 1500 years, which is longer than 450 years in the protocol, in order to be able to obtain a reasonably long equilibrium period. Technical changes were made to accommodate DBEN-relevant output variables, scramble climate years and introduce a patch-destroying disturbance in a set year. The codebase for this analysis can be accessed here: 10.5281/zenodo.15837787.

#### ORCHIDEE

ORCHIDEE is the LSM of the IPSL Earth system model (Boucher *et al.*, 2020). As an LSM, its purpose is to simulate the response of the land surface to changing environmental conditions including unprecedented climate conditions. ORCHIDEE is developed to respond to changes in: (1) atmospheric CO2 (Krinner *et al.*, 2005) (2) climate (Krinner *et al.*, 2005), (3) nitrogen inputs (Vuichard *et al.*, 2019), and (4) land cover changes (Piao *et al.*, 2009) as well as land management and changes therein (Naudts *et al.*, 2016). The response variables can be grouped as: (1) energy (among other, surface temperature and albedo) (2) water (among other, evapotranspiration and soil water content) (3) carbon (among other, net biome production and soil carbon), (4) nitrogen (among other, leaching and N2O emissions), and (5) yield (among other, river discharge and wood, grass and crop production) responses. In this study ORCHIDEE r8696 was used with its default parameters except for the number of circumference classes which was set to 10 rather than its default of three. The model was configured in its land-only configuration. Land cover changes, forest management, wind damage, and bark beetle outbreaks were not explicitly accounted for. Hence, the main cause of mortality is self-thinning.

#### SEIB-DGVM

SEIB-DGVM version 3.10 (Sato *et al.*, 2007, 2023) was employed for all simulations. The SEIB-DGVM's simulation unit is an individual tree that establishes, competes, and dies at 1-ha forest stands. Hence, the simulation results always fluctuate significantly. To reduce this fluctuation, ten repeated simulations were conducted with the same parameters but different random seeds, and these repeats were averaged.

#### CABLE-POP

CABLE-POP (Haverd *et al.*, 2018) is the land surface model CABLE coupled to the woody demography model POP (Populations-Order-Physiology, (Haverd *et al.*, 2013, 2014)). In POP, the landscape (or grid cell) is partitioned into a number of patches which differ in their time since last disturbance. Each patch contains a number of cohorts, representing size classes of a PFT. POP receives stem biomass increments from CABLE, calculates establishment, size class distribution, mortality from crowding (self-thinning) and resource limitations, and returns stem biomass turnover rates to CABLE.

#### JULES-RED

JULES-RED (Argles *et al.*, 2020) is a development that introduces demographic processes for the vegetation dynamics in the JULES LSM (Clark *et al.*, 2011). The model partitions PFT number density into plant mass bins. To update the number density between bins, the model assumes metabolic scaling theory for the growth rate and, for the simulations, size-invariant mortality. These modelling assumptions have been evaluated for large scale datasets across different biomes (Moore *et al.*, 2018, 2020). Recruitment of seedling is limited to the gap areas, which is one minus the total PFT canopy cover.

#### FATES

FATES (Fisher *et al*., 2015; Koven *et al*., 2020;)is a size- and age-structured vegetation model that runs as a component of a land surface model, currently the Community Land Model (CLM;(Lawrence *et al.*, 2019)), the Energy Exascale Earth System Model (E3SM) Land Model (ELM;(Ricciuto *et al.*, 2017)), and NorESM (Tjiputra *et al.*, 2013) (Tjiputra *et al*., 2013). Ecosystem biophysics are based on CLM4.5 (Oleson *et al.*, 2013), disturbance dynamics are based on the ecosystem demography (ED) approach (Moorcroft *et al.*, 2001), and canopy organization is based on the perfect plasticity approximation (PPA, Purves *et al*., (2008)). FATES models cohorts: groups of individuals of the same size and PFT modelled as one representative individual. Cohorts compete with each other for light on patches defined by their age since the last disturbance. Cohort dynamics are governed by the interaction between traits and environmental conditions.

#### EDv3

The EDv3 model is an global and individual-based prognostic ecosystem model (Ma *et al.*, 2022). Originally developed as a regional-scale model (Hurtt *et al.*, 1998, 2002; Moorcroft *et al.*, 2001), it has recently been calibrated and evaluated at the global scale (Ma *et al.*, 2022). The ED model integrates submodules of growth, mortality, hydrology, carbon cycle and soil biogeochemistry, ED tracks plant dynamics, including growth, mortality and reproduction. Along with plant dynamics, ED tracks the carbon cycle, including carbon uptake by leaf photosynthesis, carbon allocation to biomass growth in leaves, roots and stems, carbon redistribution from plants to soil based on plant tissue turnover from dead plants due to mortality and disturbance, carbon decomposition in various pools (metabolic litter pool, structural litter pool, soil slow pool, soil passive pool, wood product pool, harvested crop pool, etc.), as well as carbon combustion from fire. Vegetation structure (e.g., height and diameter at breast height) and physiological processes (e.g., leaf photosynthesis and phenology) are modelled at the individual scale, where individual plants compete mechanically for light, water and nutrients. The model also tracks the impacts of land-use change on vegetation dynamics. By explicitly tracking vegetation structure and incorporating land-use change impacts, ED enables potential connections with remote sensing lidar data and land-use history to simulate land carbon dynamics from past to future.

**Table S1** Individual model-PFTs mapped onto site-specific species (plant categories for BCI). For models which prescribe the PFT, fraction this is reported. Where cells are merged, species were represented by a single PFT.

| **Site** | **Species** | **PFT characteristics of species** | **Model-specific PFTs used** | | | | | | | | |
| --- | --- | --- | --- | --- | --- | --- | --- | --- | --- | --- | --- |
|  |  |  | **LPJ-GUESS** | **ORCHIDEE** | **BiomeE** | **CABLE-POP** | **EDv3** | **FATES** | **SEIB-DGVM** | **JULES-RED** | **BiomeEP** |
| **FIN** | *Pinus sylvestris* L. | Shade intolerant needleleaf | Boreal shade intolerant needleleaf evergreen (BINE) | Boreal needleleaf evergreen (BoNE) (fraction:1) | Shade intolerant needleleaf (PFT1) | Needle-leaf evergreen tree (fraction: 0.95) | - | Needleleaf evergreen extratropical tree | Cold-temperate needleleaf evergreen, late-succession (CTeNE1) | - | Shade intolerant needleleaf |
|  | *Picea abies* ([L.](https://en.wikipedia.org/wiki/Carl_Linnaeus)) [H. Karst.](https://en.wikipedia.org/wiki/Gustav_Karl_Wilhelm_Hermann_Karsten) | Shade tolerant needleleaf | Boreal shade tolerant needleleaf evergreen (BNE) | - | Shade tolerant needleleaf (PFT2) |  | - | Needleleaf evergreen extratropical tree | Cold-temperate needleleaf evergreen, middle-succession (CTeNE2) | Needleleaf evergreen tree | Shade tolerant needleleaf |
|  | *Betula pendula* Roth | Shade intolerant broadleaf deciduous | Shade intolerant broadleaf summergreen (IBS) | - | Shade intolerant broadleaf deciduous (PFT3) | Broadleaf deciduous tree (fraction: 0.05) | - | Broadleaf cold deciduous extra tropical tree | Cold-temperate broad-leaved deciduous, Early-succession, drought-tolerant (CTeBS1) + Cold-temperate broadleaf deciduous, early-succession, drought-intolerant (CT2BS2) | Broadleaf Deciduous Tree | Shade intolerant broadleaf deciduous |
|  | Grass | C3 metabolism | C3 | - | C3 | - | - | Cool C3 grass | see below | C3 Grass | C3 grass |
| **BIA** | *Picea abies* ([L.](https://en.wikipedia.org/wiki/Carl_Linnaeus)) [H. Karst.](https://en.wikipedia.org/wiki/Gustav_Karl_Wilhelm_Hermann_Karsten) | Shade tolerant needleleaf | Boreal shade tolerant needleleaf evergreen (BNE) | Temperate needleleaf evergreen (TempNE) | Shade tolerant needleleaf (PFT2) | Needle-leaf evergreen tree (fraction: 0.5) | - | Needleleaf evergreen extratropical tree | Cold-temperate needleleaf evergreen, middle-succession (CTeNE2) | Needleleaf Evergreen Tree | Shade tolerant needleleaf |
|  | *Betula* spp. | Shade intolerant broadleaf deciduous | Shade intolerant broadleaf summergreen (IBS) | - | Shade intolerant broadleaf deciduous (PFT3) | Broadleaf deciduous tree (fraction: 0.5) | - | Broadleaf cold deciduous extrtropical tree | Cold-temperate broadleaf deciduous, Early-succession, drought-tolerant (CTeBS1) + Cold-temperate broad-leaved deciduous, early-succession, drought-intolerant (CT2BS2) | Broadleaf deciduous tree | Shade intolerant broadleaf deciduous |
|  | *Carpinus betulus* L. or *Tilia cordata* Mill. | (intermediate) shade tolerant broadleaf deciduous | Temperate shade tolerant broadleaf summergreen (TeBS) |  | Shade tolerant broadleaf deciduous (PFT4) | Broadleaf deciduous tree (fraction: 0.5) |  | Broadleaf cold deciduous extratropical tree | Cold-temperate broad-leaved deciduous, late-succession, drought-tolerant (CTeBS3) + | - | Shade tolerant broadleaf deciduous |
|  | Grass | C3 metabolism | C3 | - | C3 | - | - | Cool C3 grass | Cold-temperate broad-leaved deciduous, Late-succession, drought-intolerant (CTeBS4) | C3 Grass | C3 |
| **BCI** |  | Shade intolerant tropical broadleaf evergreen | Tropical shade intolerant broadleaf evergreen (TrIBE) | - | Tropical shade intolerant evergreen (PFT5) | Broadleaf evergreen tree (fraction: 1) | - | Broadleaf evergreen tropical tree | Tropical broadleaf evergreen, pioneer, light demanding (TrBE3) + Tropical broadleaf evergreen, short, intermediate shade tolerant (TrBE4) | Broadleaf evergreen tropical tree | Tropical broadleaf evergreen shade intolerant |
|  |  | Share tolerant tropical broadleaf evergreen | Tropical shade tolerant broadleaf evergreen (TrBE) | Tropical broadleaf evergreen (TrBE) | Tropical shade tolerant evergreen (PFT6) |  | - | Broadleaf evergreen tropical tree | Tropical broad-leaved evergreen, Canopy, Shade tolerant (TrBE1) + Tropical broad-leaved evergreen, Sub-canopy, Shade tolerant (TrBE2) | Broadleaf deciduous tree | Tropical broadleaf evergreen shade tolerant |
|  |  | Tropical broadleaf deciduous | Tropical shade tolerant broadleaf raingreen (TrBR) | - | 4 Tropical drought-deciduous (PFT7) | Broadleaf evergreen tree (fraction: 1) | - | Broadleaf hydro-deciduous tropical tree | Tropical broad-leaved rain-green (TrBR) | - | Tropical broadleaf deciduous |
|  |  | C4 metabolism | C4 | - | C4 | - | - | C4 grass | C3-type photosynthesis grass (TeH) + C4-type photosynthesis grass (TrH) | C4 Grass | C4 |

**Table S2** Description of model-specific modes of biomass reductions that was invoked to enable the complete forest removal after 30 years of simulation. It also documents the capabilities of the models with regards to representing such an event.

| **Model** | **Mode of biomass reduction** | **Notes** |
| --- | --- | --- |
| LPJ-GUESS | Patch-destroying disturbance | A feature of LPJ-GUESS that mimics a natural disturbance event which kills all trees. |
| JULES-RED | Mortality rate increase | reduction of biomass down to 97.5% of vegetation (stems, but size independent selection so should be linear with biomass). |
| CABLE-POP | Patch-destroying disturbance | A disturbance event that destroyed all 60 patches was simulated. |
| ORCHIDEE | Stand-replacing disturbance | A configuration of ORCHIDEE that mimics a natural disturbance event which kills all trees and adds their biomass to the plant litter. |
| SEIB-DGVM | Stand replacing fire | For woody PFTs, all trees were killed, and their above ground biomass lost. For grass PFTs, all aboveground biomass is lost. |
| FATES | Mortality rate increase | Mortality of tree PFTs was increased to 100% for a single year and then reset to pre-disturbance levels. |
| EDv3 | Patch-destroying disturbance | All cohorts of all patches are removed and reset to the initial cohort structure. |
| BiomeE | Reset vegetation to initial state | Replace current cohorts with the initial cohorts (where the plant size is small). |
| BiomeEP | Reset vegetation to initial state | Replace current cohorts with the initial cohorts (where the plant size is small). |

**Table S3** Parameters adjusted from default parameter set to obtain P0 output. Parameters are labelled by the processes they are involved in: photosynthesis/productivity, carbon allocation, mortality, reproduction and establishment, structural, other physiology or other. The adjusted parameters or factors still remain within plausible ranges of observations.

| **Model** | **Parameter adjusted** | **Notes** |
| --- | --- | --- |
| LPJ-GUESS | FIN: default parameters  BIA: default parameters  BCI: *structural*: for all PFTS (TrBE, TrBR and TrIBE)  maximum crown area = default: 50. Updated: 130 m2  leaf to sapwood area ratio = default: 6000 updated: 10000 | Default parameters are from Smith *et al*., (2014). The updated values are based on observations and lead to an improved fit against all variables at BCI. These updated values have previously been found to give more realistic dynamics for Tropical Forests than the default values (Pugh *et al*., 2019). |
| BiomeE | FIN: Default parameters  BIA: *mortality*: Annual background mortality of PFT3 (Intermediate shade-tolerant broadleaf deciduous) was adjusted. | Size-related mortality activated. |
| SEIB-DGVM | FIN: Default parameters  BIA: *mortality*: Annual background mortality of PFT4 (intermediate shade-tolerant broadleaf deciduous) was adjusted from 0.95 to 0.040.  BCI: Default parameters |  |
| FATES | FIN: *structural*: Allocation to carbon storage was increased for the two needleleaf PFTs to 3.3 and to 2.4 for the broadleaf PFT.  Reproduction and establishment: Seed allocation increased to 0.015 for all PFTs. Seed germination rate decreased for the broadleaf PFT to 0.1. Seed decay was increased for the broadleaf PFT to 0.8. SLA was increased for the two needleleaf PFTs to 0.008.  *Other physiology*: Leaf maintenance respiration base rate was decreased for the two needleleaf PFTs to 1.406. The vertical scaling of respiration was increased in the needleleaf PFTs.  *Mortality*: Background mortality was increased for the light demanding needleleaf to 0.017, decreased for the shade tolerant needleleaf to 0.011 and increased for the broadleaf PFT to 0.02. We turned on size dependent mortality with an inflection point parameter of 250 and 175 for needleleaf and broadleaf PFTs, and rate parameters of 0.04 for all tree PFTs.  Branch turnover decreased to 50 years for the broadleaf PFT. The disturbance fraction was set to 0.1.  BIA: *structural*: allocation to carbon storage increased to 3.23 for the needleleaf PFT and 2.28 for the broadleaf PFTs.  Other physiology: growth respiration was decreased for the needleleaf PFT to 0.09 and increased for the broadleaf PFTs to 0.12. SLA increased for the needleleaf PFT to 0.008 and leaf maintenance respiration base rate decreased to 1.214.  *Mortality:* background mortality increased to 0.015 for the light demanding broadleaf PFT and decreased to 0.013 for the shade tolerant broadleaf PFT. We turned on size-dependent mortality with inflection point of 375 and rate of 0.2 for all tree PFTs (the large inflection point meant it had little effect in practice).  We used a disturbance fraction of 0.2.  *Reproduction and establishment:* we decreased allocation to seeds to 0.05 for the needleleaf and increased it to 0.15 for the broadleaf PFTs. We decreased the seed germination rate to 0.1 for the needleleaf and increased it to 0.55 for the broadleaf PFTs. Branch turnover was increased to 75 years for the broadleaf PFTs.  *Photosynthesis:* Vcmax decreased to 52 for the needleleaf PFT, 49.5 for the light demanding broadleaf and 48.5 for the shade tolerant broadleaf PFT.  BCI: *structural:* allocation to carbon storage decreased to 1.742 for the drought-deciduous PFT.  SLA top was decreased to 0.0217 for the drought-deciduous PFT. *Mortality:* background mortality rate was increased for the light-demanding and drought-deciduous PFTs to 0.0183.  *Photosynthesis:* Vcmax was increased for the light demanding and drought-deciduous PFTs to 51.105.  *Other physiology:* leaf maintenance respiration used the Atkin *et al.,* (2017) model for all sites.  Deterministic height sorting was used for canopy organisation (for FIN and BIA).  *Structural*: older versions of allometric parameters were used. | FATES PFTs are largely uncalibrated and do not include early and late successional PFTs. Multiple adjustments were made to divide biome level PFTs into early and late successional. We initially ran an ensemble with Latin hypercube sampling to find reasonable parameter sets for each biome, and then hand tuned parameters to get closer to benchmarking datasets. |
| ORCHIDEE | FIN: default parameters.  BIA: default parameters.  BCI: default parameters. | Each test location was simulated as a single PFT. The number of circumference classes in the model was set to 10. These 10 classes were remapped on the diameter classes used in this study. Remapping resulted in fewer classes of the output files being populated then the number of classes in ORCHIDEE. |
| EDv3 | *Photosynthesis/productivity*: GPP was adjusted using a site-specific scaling factor to ensure that the simulated AGcwood falls within the range of reference data. The scaling factors for FIN, BIA and BCI are 1.1, 2.0, and 0.8 respectively. |  |
| BiomeEP | FIN: default parameters.  BIA: default parameters.  BCI: default parameters. | Size-related mortality activated. |
| CABLE-POP | FIN:  *Structural:*  Ksapwood: 0.04  Kbiometric: 35  *Mortality:*  CrowdingFactor: 0.1  Pmort: 4.0  GE_min: 0.016  Mort_max: 0.15  BIA:  *Structural:*  Ksapwood: 0.05  Kbiometric: 20  *Mortality:*  CrowdingFactor: 0.06  Pmort: 2.25  GE_min: 0.012  Mort_max: 0.15  BCI:  *Structural:*  WoodDensity: 300  KSapwood: 0.11  Kbiometric: 20  *Mortality:*  CrowdingFactor: 0.05  Pmort: 1.2  GE_min: 0.01  Mort_max: 0.12 | The following parameters were manually adjusted to site conditions using observations of stem size distribution, biomass from regrowth curves, and equilibrium biomass.  Ksapwood: sapwood turnover rate (default: 0.067 yr-1)  Kbiometric: parameter in height-diameter relationship (default: 50.0)  CrowdingFactor: Parameter in crowding mortality formulation (Eq. A11 in Haverd *et al*., 2014, default: 0.043)  Pmort: Exponent in resource mortality equation (Eq. A10 in Haverd *et al*., 2014, default: 5.0).  GE_min: minimum Growth efficiency resource mortality equation (Eq. A10 in Haverd *et al*., 2014, default: 0.012).  Mort_max: Maximum mortality rate in resource mortality equation (Eq. A10 in Haverd *et al*., 2014, default: 0.3 yr-1).  WoodDensity: Default: 340 kg m-3 |
| JULES-RED | Site: Relevant PFT (*Mortality, assimilate for reproduction fraction*)  FIN: BDT (0.025, 0.06), NET (0.0275, 0.06), C3 (0.125, 0.6)  BIA: BDT (0.002, 0.08), NET (0.025, 0.06), C3 (0.125, 0.6)  BCI: BET-Tr (0.05, 0.045), BDT (0.05, 0.04), C4 (0.125, 0.6)  Default Parameters  In order of: BET-Tr, BET-Te, BDT, NET, NDT, C3, C4, ES, DS  Mortality: 0.05,0.05,0.05,0.05,0.05,0.125,0.125,0.075,0.075  Reproduction: 0.06,0.06,0.06,0.06,0.06,0.6,0.6,0.15,0.15 | Adjusted the mortality and fraction of assimilate to reproduction. JULES PFTs that were not relevant for the sites assumed the default mortality and recruitment parameters.    JULES PFTs:  BET-Tr: Broadleaf Evergreen Tropical Tree  BET-Te: Broadleaf Evergreen Temperate Tree  BDT: Broadleaf Deciduous Tree  NET: Needleleaf Evergreen Tree  NDT: Needleleaf Decidious Tree  C3: C3 Natural Grass  C4: C4 Natural Grass  ES: Evergreen Shrub  DS: Deciduous Shrub |

**Table S4** Mapping of model-output variables to D-BEN variables, where these deviate from the “D-BEN” definition (Table S3.1 in Notes S1). Additional variables not used in D-BEN but relevant to the analysis where D-BEN output could not be developed in time are also reported.

| **D-BEN variable** | **FATES** | **ORCHIDEE** | **JULES-RED** | **CABLE-POP** | **BiomeEP** | **BiomeE** | **SEIB-DGVM** | **EDv3** |
| --- | --- | --- | --- | --- | --- | --- | --- | --- |
| cmort | contains non-woody biomass | “cmort” at stand-level; “cmort_size” at cohort-level. | no sizeclasses <1 |  |  |  |  |  |
| cwood |  | stand-level | no sizeclasses <1 |  |  |  |  |  |
| nstem_size |  | cohort-level | no sizeclasses <1 |  |  |  |  |  |
| cwood_size |  | cohort-level | no sizeclasses <1 |  |  |  |  |  |
| cmort_size |  | cohort-level | no sizeclasses <1 |  |  |  |  |  |
| AGcwood |  | stand-level | AGB for BIA and BCI, “AGB”-with leaves removed for FIN |  |  | AGB, leaves negligible |  |  |
| AGcwood_size (Figure 2) | cwood_size*0.75 | cwood_size | cwood_size*0.75 | cwood_size*0.75 | cwood_size*0.75 | cwood_size*0.75 | cwood_size*0.75 | cwood_size*0.75 |
|  | New variables: cveg_tot, Bgrowth |  |  |  |  |  |  |  |

**Table S5** Upper and lower simulation year that make up the period from which each model is considered in equilibrium. Some models were run for a longer time period than the prescribed 450 years in order to achieve a mature forest in equilibrium state. The equilibrium period was selected by the modelling teams, in cases where an C_wood,AG_  -dynamic equilibrium was not immediately obvious.

| lower | upper | model | site |
| --- | --- | --- | --- |
| 715 | 810 | BiomeE-Standalone | FIN |
| 700 | 810 | BiomeE-Standalone | BIA |
| 500 | 810 | BiomeE-Standalone | BCI |
| 600 | 900 | BiomeEP | FIN |
| 600 | 900 | BiomeEP | BIA |
| 600 | 900 | BiomeEP | BCI |
| 320 | 450 | CABLE-POP | FIN |
| 320 | 450 | CABLE-POP | BIA |
| 320 | 450 | CABLE-POP | BCI |
| 500 | 530 | EDv3 | FIN |
| 500 | 530 | EDv3 | BIA |
| 500 | 530 | EDv3 | BCI |
| 300 | 450 | FATES | FIN |
| 300 | 450 | FATES | BIA |
| 250 | 450 | FATES | BCI |
| 200 | 420 | JULES-RED | FIN |
| 200 | 420 | JULES-RED | BIA |
| 150 | 420 | JULES-RED | BCI |
| 320 | 450 | LPJ-GUESS | FIN |
| 320 | 450 | LPJ-GUESS | BIA |
| 320 | 450 | LPJ-GUESS | BCI |
| 150 | 320 | ORCHIDEE | FIN |
| 150 | 320 | ORCHIDEE | BIA |
| 150 | 320 | ORCHIDEE | BCI |
| 300 | 420 | SEIB-DGVM | FIN |
| 240 | 450 | SEIB-DGVM | BIA |
| 100 | 450 | SEIB-DGVM | BCI |

**Methods S2** Demographic carbon balance

Since demographic models track the carbon of individuals/cohorts, their carbon mass at recruitment, growth increment, carbon content and finally the carbon loss at death has to be accounted for. Normally models have to show that their overall carbon balance between carbon incoming and outgoing to the atmosphere (e.g. gross primary productivity - (respiration + mortality) = 0) closes. However, the reporting and accounting of “demographic” fluxes is more complicated, which created the need for such a test. In order to correctly compare forest stand WBgrowth, cmort or cwood to observations, we have to make sure that the variables contain the appropriate values.

We allow for some variability in the accounting and define the demographic carbon balance as the mean between the absolute difference of cwood pool output and flux-derived cwood pool output (from regrowth to year 450) should be smaller than 10% of mean equilibrium cwood (Eq S1):

$\frac{1}{450}\sum_{t=31}^{450} \left| {cwood}_{t}-\sum_{i=31}^{t} {(WBgrowth}_{i}-{cmort}_{i}) \right|<0.1 \bar{cwood}_{equilibrium}$ (Eq S1)

Equilibrium suffix: The equilibrium period length was expert judgement for testing, for the purpose of comparison in this study, the equilibrium period was set to the last 60 simulation years.

#### CABLE-POP demographic carbon imbalance

Reasons for the non-closure of the carbon balance in CABLE-POP could not be identified but are likely due to inconsistencies in the aggregation from cohort and patch to landscape level and the associated non-linearities in the calculations (e.g. the patch weightings as described in (Haverd *et al*., 2014).

#### BiomeEP demographic carbon imbalance

The carbon balance in BiomeEP did not fully close for all the sites, likely because of hidden issues when grouping cohorts into forest-level outputs.


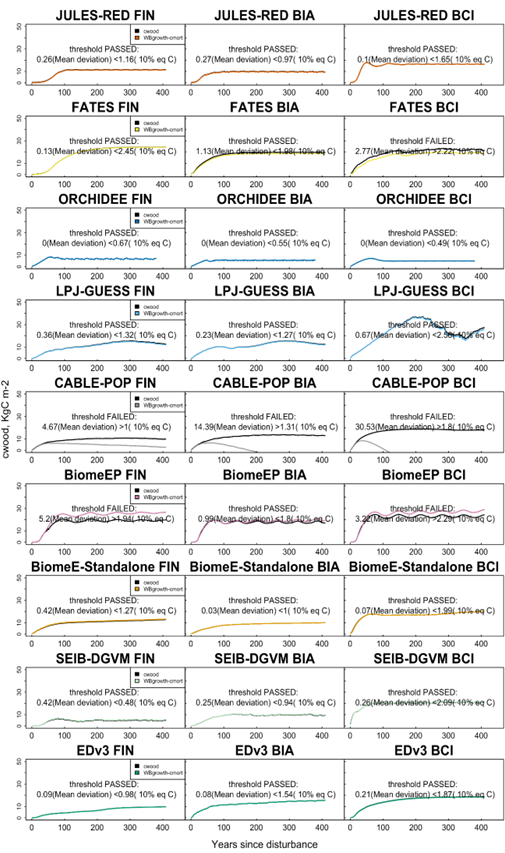


**Fig. S1** Demographic (woody) carbon budget test results, and visualisation of the temporal trajectory of the outputted carbon pool variable C_wood_, and the flux-derived carbon pool.

**Notes S1** Simulation protocol used to perform the benchmarking simulations.

- See file Supplementary_Notes_1_simulation_protocol

Screenshot of content:

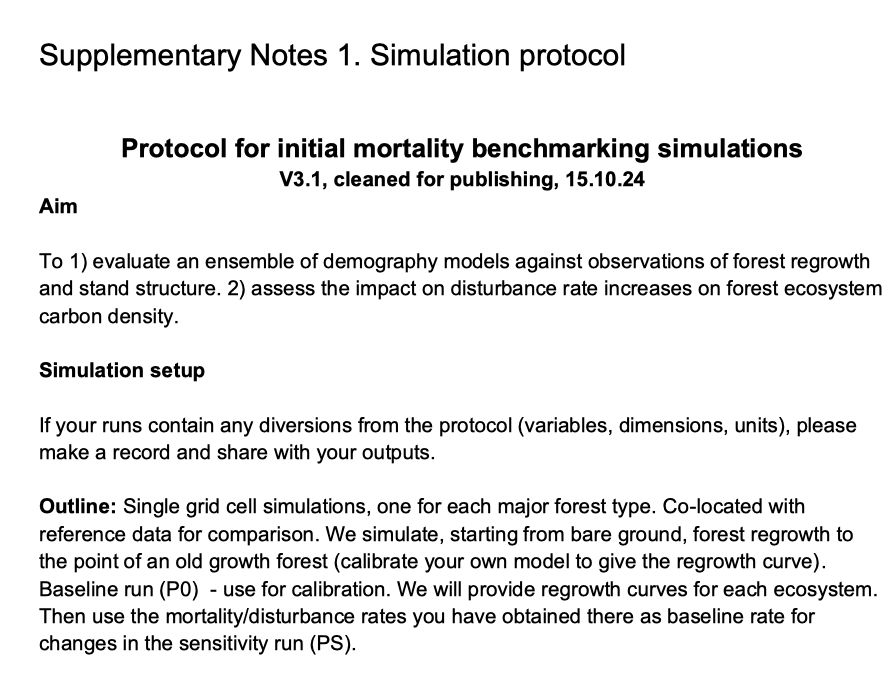


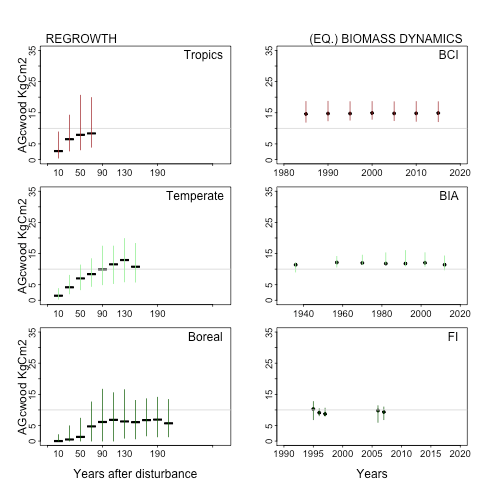


**Fig. S2** Observed chronosequence regrowth (left) and mature forest dynamics (right) benchmarking data. The horizontal bars in the REGROWTH plots highlight that data was binned in 20-year age bins. Vertical bars in the REGROWTH plots show the observational data ranges within the 10^th^ and 90^th^ percentile of the data. Vertical bars in the (Eq.) BIOMASS DYNAMICS plots show the full observational data range (also see table S6).

####
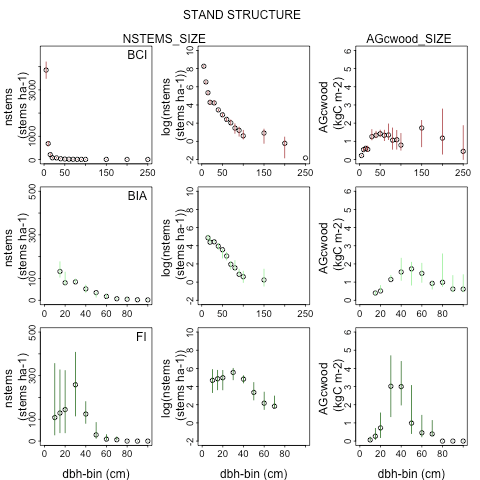


**Fig. S3** Benchmarking data of stand structure, number of stems (left two columns), and woody biomass by size class (right). Data for BCI are taken from median, and upper and lower confidence interval values from spatial bootstrapping (6.25ha) of a 50ha plot with 95% confidence interval, for the latest year recorded (2015). Data for BIA, is the median of five sites, alongside the minimum and maximum across all sites for each dbh-class for the latest year recorded (2012). Data for FIN is based on median, maximum and minimum of 57 sites, from the census conducted in years 2006-2007 (also see table S6).


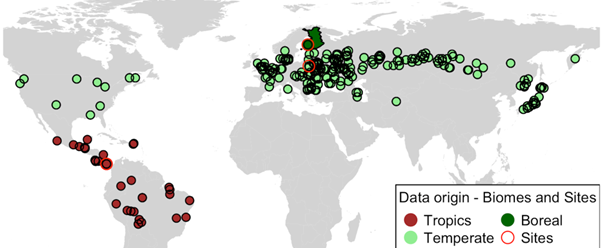


**Fig. S4** Distribution of data used for this study. Single sites used for site-based comparison of dynamic equilibrium woody biomass and stand structure are highlighted in red. Other data points are used to derive the regrowth chronosequence against which to compare the site regrowth. Tropical site: BCI, Barro Colorado Island; Temperate site: BIA, Bialowieza in Poland; Boreal: FIN, in southern Finland.

**Table S6** Benchmark data overview in terms of range, type and justification for the method to derive observed ranges, alongside with some comments/observations on the behaviour of the data, and filtering criteria.

| **Benchmark** | **Site** | **Origin** | **Spatial domain** | **Range** | **Number of observations per benchmark value** | **Method of obtaining variability range in benchmark** | **Comments** | **benchmarking filename:** |
| --- | --- | --- | --- | --- | --- | --- | --- | --- |
| Regrowth, C_wood,AC_ | FIN, BIA, BCI | Repo *et al*., (2021); Teobaldelli *et al*., 2009; Poorter *et al*,. 2016 | landscape | Lower boundary: 10^th^ upper boundary: 90^th^ percentile | 61 – 725 (Tropics)  58 – 412 Temperate)  35 – 481 (Boreal /Fin) | Percentile ranking of values within each age bin (n>20). Median reported. | Especially during early ages in regrowth data, many sites have little carbon mass/establishment and the data is skewed to low end. Older age bins with less than 20 were omitted and usually are made up of lower biomass values than younger mature forest age bins. The number of samples in each age bin determining the range is always highest for young forest, and lowest for the highest-age forest. We did not filter out arid or less fertile sites from Poorter (2016) but used the whole dataset for chronosequence creation. Poorter spans the whole Neotropics. Teobaldelli uses a combined and cleaned dataset from Usoltsev (2001) and Cannell (1982) spanning a wide spatial domain (Fig S3). Note that the tropical data is AGB, and not C_wood,AC._ | benchmark_regrowth_curves.csv ; available a priori |
| Mature forest C_wood,AC_ | FIN | Peltoniemi & Mäkipä 2011 | site |  | 5-30 | Percentile ranking of single site values. Median reported | Some years with <5 site records exist, and are in the same range as the benchmarks, but are not used here. | benchmark_eq_dynamics.csv ; available a priori |
|  | BIA | (Brzeziecki *et al.*, 2016) (Włoczewski, 1954; Brzeziecki *et al*., 2016). | site | Lower boundary: min boundary: max | 30 | All values used. Median reported | Because we only have 5 values per year (5 years), one for each site, we allow for all values to make up the plausible range of annual values in this benchmark. There is no large variability in the range, but the median is normally close to the lower ranges |  |
|  | BCI | Condit *et al*., 2019; Davies *et al*., 2021) | site | 95% CI | NA | Spatial Bootstrapping over 6.25 ha quadrants with 1000 replicates, median. | Creating pseudoreplicate stands within an area of 50ha, by sampling stands of size 6.25. |  |
| Mature forest turnover times | FIN | (Peltoniemi & Mäkipää, 2011) | site |  | 7-59 sites , nyears variable, depending on site |  | Pre-processed into following methods S3 and benchmark variables extracted from this format. | benchmark_turnover_times.csv |
|  | BIA | (Włoczewski, 1954; Brzeziecki *et al*., 2016). | site |  | 5 site ,6 years |  | directly used all TreeMort post-processed database entries for all six censuses for all five sites. n=30 |  |
|  | BCI | (Condit *et al.*, 2019; Davies *et al.*, 2021) | site |  | 1 site ,6 years |  | Pre-processed following methods S3 |  |
| Mature forest WBgrowth | FIN, | Peltoniemi & Mäkipä 2011 | site |  | 7-59 sites, nyears variable, depending on site |  | Pre-processed following methods S3 format and benchmark variables extracted from this format. | benchmark_WBgrowth.csv |
|  | BIA | Włoczewski, 1954; Brzeziecki *et al*., 2016). | site |  | 5 site ,6 years |  | Pre-processed following methods S3 ; entries for all six censuses for all five sites. n=30 |  |
|  | BCI | Condit *et al*., 2019; Davies *et al*., 2021) | site |  | 1 site ,6 years |  | Pre-processed following methods S3 |  |
| Mature forest Stand Structure, nste_size and cwood_size | FIN | Peltoniemi & Mäkipä 2011 | site | Median  Lower boundary: 10^th^ upper boundary: 90^th^ percentile | 7-59 | All values used | Size classes of all sites are pooled for the oldest available sampling year of a monitoring site, which can be 1991, 1995, 2006 or 2007, assuming that the later years in the sites always contain 'older' forest. | benchmark_stand_structure.csv ; available a priori |
|  | BIA | Włoczewski, 1954; Brzeziecki *et al*., 2016). | site | Lower boundary: min boundary: max | 5 | All values used | Size classes of all sites are pooled, data from year 2012 census was used. |  |
|  | BCI | Condit *et al*., 2019; Davies *et al*., 2021) | site | 95% CI | NA | Spatial bootstrapping | CIs are quite wide for large-size classes because of the naturally low sample number of big trees. |  |

#### Methods S3 Observational Data

#### Biome-level *C*_wood,AG_ regrowth curves

For temperate biome-level data [(Teobaldelli *et al*., 2009)](https://www.zotero.org/google-docs/?BEU6lX), we filtered first by latitude (>23°, <60°) and selected for broadleaf deciduous species. We subsequently removed remaining species that are tropical, subtropical or evergreen or do not reflect the growth habit of PFTs we simulate at BIA.  While evergreens are part of the species mix at BIA (40%), we omit pure evergreen sites as their dynamics may otherwise be skewing the observations of regrowth dynamics for BIA. To obtain *C*_wood,AG_ only, observations which contained non-woody carbon pools were removed from the dataset.

For boreal biome-level data, we used biomass chronosequence data calculated for Repo *et al.,* [(2021)](https://www.zotero.org/google-docs/?ZC5G3M) based on Finnish national forest inventory plots (Korhonen *et al.*, 2021). To arrive at aboveground woody carbon, we removed the foliage biomass fraction based on stand-age-based biomass expansion factors (BEFs) using age-dependent foliage-total biomass relationships calculated from Lehtonen *et al.,* (2004).

Tropical data [(Poorter *et al*., 2016)](https://www.zotero.org/google-docs/?D55Ek4) required no post-processing (except for a unit conversion from kg dry matter to kg carbon), as we follow the common assumption that while aboveground total biomass is reported, this is roughly equivalent to aboveground woody biomass (for more details see the section on deriving mature forest C_wood,AG_ dynamics for BCI).

#### Mature forest woody turnover time (*τ)* and Woody biomass growth (WBgrowth)

WBgrowth fluxes and turnover time benchmarks were derived from tree-level data, aggregated to plot-level and are based on allometric equations that calculate aboveground biomass (AGB) rather than aboveground woody biomass (C_wood,AG_). However, this discrepancy is disregarded because of the lack of leaf-expansion factors for all species and mitigated by the supposition that AGB and C_wood,AG_ represent consistent and proportional quantities at the stand level. It is important to note that WBgrowth, here approximated using annual biomass productivity (P), is likely slightly overestimated in the observations due to the inclusion of a leaf fraction in aboveground biomass (AGB). Nevertheless, turnover time, which depends on relative changes, remains unaffected and is directly comparable against simulated turnover time.

For the European temperate and boreal trees we estimated AGB using allometric equations from Forrester *et al*., (2017) that use stem diameter and, in some cases, height. When available, species-specific equations were used such as eq 3 for *Picea abies* ([L.](https://en.wikipedia.org/wiki/Carl_Linnaeus)) [H. Karst.](https://en.wikipedia.org/wiki/Gustav_Karl_Wilhelm_Hermann_Karsten), *Pinus sylvestris* L. and *Betula pendula* Roth, and eq. 23 for *Carpinus betulus* L. with parameters from Table 2 or Table A1 in Forrester *et al*., (2017). To estimate AGB of tropical trees, we used allometric equations from Chave *et al*., (2014), AGB and height estimations were calculated adapting the functions from the R package BiomasaFP (ForestPlots.net *et al.*, 2021).

Annual biomass loss L was estimated sensu Kohyama *et al.,* (2019):


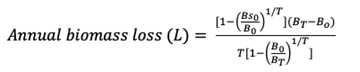
 (Eq. S2)

where T is the time (t) interval between census at t = 0 and t = T ; B_0_ and B_T_ are the AGB at t = 0 and t = T, and B_S0_ is the initial AGB at t = 0 for survivors of interval T. This approach accounts for heterogeneity in vital rates within the population. Annual AGB productivity, here assumed to be similar to WBgrowth was calculated as the per difference in B between t = 0 and t = T:


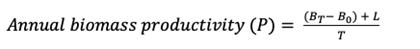
 (eq. S3)

The latter approach does not account for any trees which recruit and die during the census period but avoids the incompatibilities of complex methods (Kohyama *et al.*, 2018) with the variable sampling intensity in space found in concentric or angle-count sampling protocols.

Turnover time*𝜏* is calculated from AGB and mortality flux:

$\tau=\frac{AGB}{L}$ (eq. S4)

**Mature forest *C*_wood,AG_ dynamics**.

*C*_wood,AG_ dynamics for BIC was obtained from six censuses, from 1990 and every five years thereafter. Lianas and strangler figs were removed from the analysis. Total aboveground biomass (AGB) was estimated for each tree at each census from the measured DBH and wood density using the pantropical broadleaf allometric equation from Chave *et al*., , eq. 7 and Réjou-Méchain *et al*., (2017). The DBH of trees measured at a height >1.3 m was corrected based on a taper model to obtain the corresponding measurement at 1.3 m (Cushman *et al.*, 2021). Wood density (WD) was obtained using the R package BIOMASS (Réjou-Méchain *et al.*, 2017). The resulting estimate is often assumed to be woody AGB because in the tropics the fraction of AGB in leaves, fruits, flowers, etc., is very low, with leaf biomass being between 5% (Delitti *et al.*, 2006) and 2% from the total aboveground biomass (>25cm dbh) (w/ Helene Muller-Landau pers comm.) depending on the size of the trees (Delitti *et al.*, 2006). To obtain an upper and lower observational range, we performed bootstrapping over subplots of 250m x 250m (6.25 ha) with 1,000 replicates and report 95% confidence interval and median for each year.

For BIA, we used old-growth forest data from C_wood,AG_ is based on AGB from Forrester *et al*., (2017). We report the median, maximum and minimum of the five data points.

For FIN, we calculated C_wood,AG_ based on regional species-specific allometric equations (Repola, 2008, 2009), also removing the conifer foliage fraction (Repola, 2009) to avoid bias on C_wood,AG._ To provide complete sets of data to generate allometric relationships, tree height data had to be gap-filled using the randomForest (Liaw & Wiener, 2002) package in R (R Core Team, 2023), default settings, using predictors DBH, log(DBH), latitude, maximum measured tree height at the site, site quality, and species. This gap-filled data was also used to calculate stand structure benchmarks as presented below. We dropped years where <5 sites report biomass values before calculating median, and 95% percentile ranges values.

**Methods S4** Determination of the self-thinning period and slope

Data on C_wood,AG_ and nstems was extracted from chronosequence data in Teobaldelli *et al*., (2009),where we also kept datapoints on stem number/ha, which were marked as “indirect estimation”. We extracted for Broadleaf groups and conifer groups for comparison against BIA and FIN respectively. Modelled periods of when self-thinning is strongest were extracted based on a combination of identification criteria of the self-thinning period: 1) Where models contain diagnostic output for multiple mortality mechanisms explicitly (i.e. LPJ-GUESS[greff+thinning], CABLE-POP[crowding], FATES[cstarv]), the selection is based on the 95th percentile of self-thinning related mortality rates explicitly ( thinning/competition based mortality occurs to some extent during any time of the simulation (except directly after establishment), so a threshold above which self-thinning is the dominant mechanism that causes the self-thinning boundary had to be chosen to account for this.

For models with no explicit thinning-related mortality mechanism, the thinning period was either 2) chosen as the period of the 95th percentile of total mortality rate (e.g. BiomeE), or 3) chosen semi-automatically, as the consecutive points between the furthest to the bottom-left and the top-right points in the timeseries in self-thinning space. Lastly (4) the period was adjusted manually, so that the trajectory of the simulation reflects what looks like a thinning trajectory.


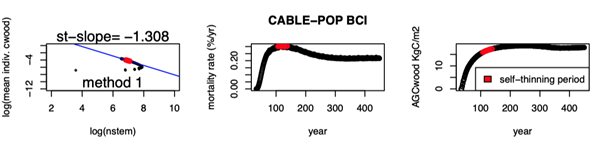


**Fig. S5** Example of the plots that can visualise the self-thinning period and length. Plot 1(left) black dots are model output on average individual biomass and stem numbers over time, in "self-thinning space" (usually starting from bottom-left, and then moving over to bottom right, then up to the top right). The coloured dots are the data points used to fit the self-thinning line. Plot 2 (center) mortality rate over the course of regrowth, plot 3 (right) C_wood_ trajectory over the course of regrowth; the dots that coincide with self-thinning are highlighted in red. The self-thinning slope is reported as title of the first plot, and also in Table S7 below. Visualisations of the self-thinning period as part of the whole simulation period for each model and each site are found in Fig S10.


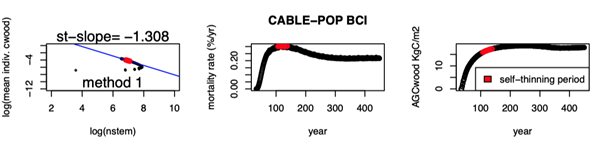


**Fig. S6** An example of using method 1. The example uses CABLE-POP at Barro Colorado Island, where the self-thinning period is automatically identified as all instances where the mortality rate is above the 95^th^ percentile of a thinning-related mortality mechanism, in the case of CABLE-POP: crowding mortality ( see middle plot for crowding mortality, the 95^th^ percentile of the rates are highlighted in red).


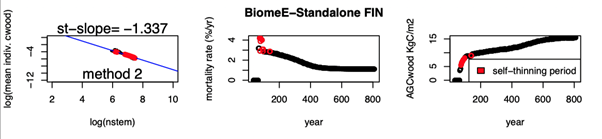


**Fig. S7** An example of using metho 2d. The example uses BiomeE at Finland, where the self-thinning period is automatically identified as all instances where the mortality rate is above the 95^th^ percentile of total mortality).


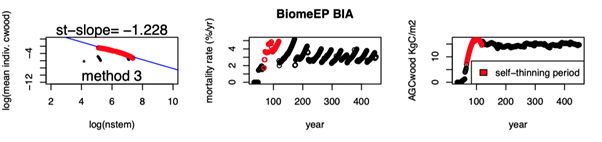


**Fig. S8** An example of using method 3. The example uses BiomeEP at Bialowieza, where the self-thinning data itself (shown in the first plot) is used to automatically determine the self-thinning period: The period selected is all consecutive years that fall between the “most bottom-right” point, to the “most top left” point, as these are all aligned along the emergent self-thinning behaviour of the model. The selected points are then highlighted in red in the other two plots, on the mortality and the C_wood,AG_  timeseries and align well with the timing of initial self-thinning during early establishment. Auto-adjusted self-thinning periods can have some small manual adjustments, if large outliers are within the timeseries. They are still considered auto-adjusted sequences


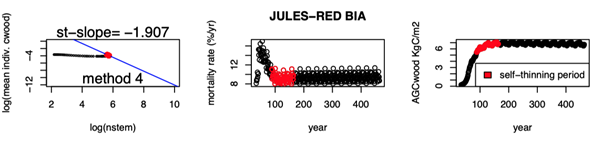


**Fig. S9** An example of using method 4. The example uses JULES-RED, where self-thinning period was manually adjusted, where method 3 selected inappropriate values. Manual adjustment was still made trying to adhere to selecting the years which are the most likely to align along a self-thinning slope trajectory. The upper and lower boundary-years are reported in Table S8 below.

For method 4 (Figure S9), where in doubt, the model developer was consulted in the selection of the points (Figure S10). For example, the basic principle of the self-thinning was documented as in the below graph, and the general behaviour of the model plotted onto it (step 1). Then, the self-thinning plot of the model was shown, and the starting and endpoint suggested highlighted, and confirmed with the developer, with potential iterations (step 2).

**
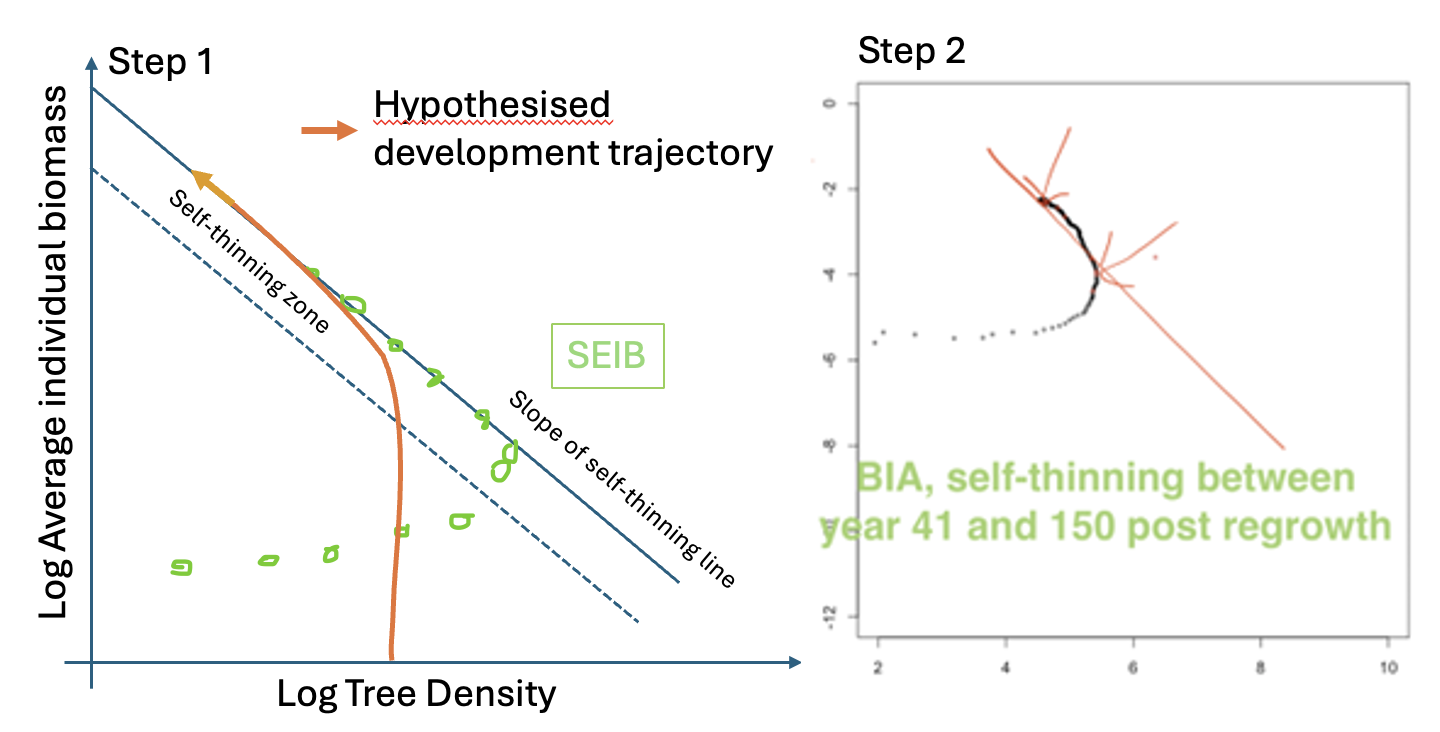
**

**Fig. S10** Two-step process for manual self-thinning selection method 4. Step 1: Modified from Figure 9, (Powell, 2000). SEIB-DGVM general behaviour was plotted into the figure to contextualise its dynamics with general self-thinning behaviour. Step 2: An example of SEIB-SGVM at BIA in “self-thinning space” and the procedure of selecting the appropriate start and end point for the self-thinning period.

**Table S7** Self-thinning selection method per model and site and resulting duration and self-thinning slope identification methods 1-4 are briefly explained:1) from self-thinning-related mortality above 95th percentile of that mortality rate, 2) from total mortality above 95th percentile of total mortality rate, 3) automatic adjustment: selection of the consecutive points between the furthest to the bottom-left and the top-right points in the timeseries in self-thinning space, replicating the self-thinning trajectory. 4) manual adjustment: start and end were selected manually by eyeballing the points in self-thinning space and selecting those points that replicate the most a self-thinning trajectory

| Model | site | lower | upper | duration | id_method | slope |
| --- | --- | --- | --- | --- | --- | --- |
| BiomeE-Standalone | BCI | 14 | 106 | 92 | 2 | -1.49 |
| BiomeE-Standalone | BIA | 55 | 118 | 63 | 3 | -1.17 |
| BiomeE-Standalone | FIN | 33 | 71 | 38 | 2 | -1.76 |
| BiomeEP | BCI | 25 | 93 | 68 | 3 | -1.26 |
| BiomeEP | BIA | 31 | 95 | 64 | 3 | -1.17 |
| BiomeEP | FIN | 36 | 115 | 79 | 3 | -1.31 |
| CABLE-POP | BCI | 72 | 106 | 34 | 1 | -1.33 |
| CABLE-POP | BIA | 88 | 216 | 128 | 1 | -1.55 |
| CABLE-POP | FIN | 55 | 175 | 120 | 1 | -1.38 |
| EDv3 | BCI | 31 | 55 | 24 | 4 | -1.69 |
| EDv3 | BIA | 12 | 42 | 30 | 2 | -1.49 |
| EDv3 | FIN | 43 | 86 | 43 | 2 | -1.10 |
| FATES | BCI | 18 | 30 | 12 | 1 | -2.36 |
| FATES | BIA | 46 | 107 | 61 | 1 | -1.70 |
| FATES | FIN | 115 | 290 | 175 | 3 | -1.73 |
| JULES-RED | BCI | 42 | 80 | 38 | 3 | -0.74 |
| JULES-RED | BIA | 55 | 131 | 76 | 4 | -1.77 |
| JULES-RED | FIN | 101 | 161 | 60 | 3 | -1.00 |
| LPJ-GUESS | BCI | 40 | 119 | 79 | 1 | -1.56 |
| LPJ-GUESS | BIA | 21 | 105 | 84 | 1 | -1.34 |
| LPJ-GUESS | FIN | 41 | 115 | 74 | 1 | -1.66 |
| ORCHIDEE | BCI | 11 | 283 | 272 | 3 | -1.06 |
| ORCHIDEE | BIA | 8 | 108 | 100 | 3 | -1.15 |
| ORCHIDEE | FIN | 1 | 257 | 256 | 3 | -1.52 |
| SEIB-DGVM | BCI | 10 | 25 | 15 | 4 | -1.82 |
| SEIB-DGVM | BIA | 41 | 150 | 109 | 4 | -2.01 |
| SEIB-DGVM | FIN | 50 | 100 | 50 | 4 | -1.42 |

**Fig. S11** Self-thinning method selection for each individual model and site*.* Column 1) grey dots are model output in log10 of average individual biomass and stem numbers over time, in "self-thinning space" (usually starting from bottom-left, and then moving over to bottom right, then up to the top right). The coloured dots are the data points used to fit the self-thinning line. Column 2) mortality rate over the course of regrowth, column 3) C_wood_ trajectory over the course of regrowth; the dots that coincide with self-thinning are highlighted in red

See file FigS11_Supplementary_materials_Benchmark_self_thinning_all_models_point_selection_methods_3plots.pdf

- Screenshot of content:


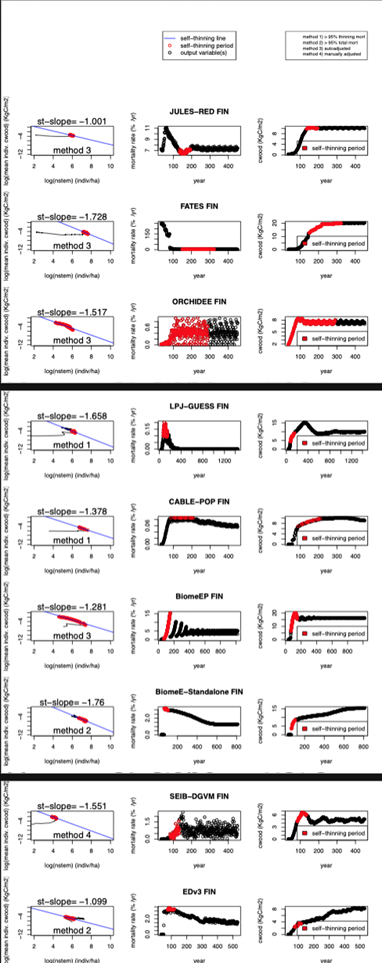


**Table S8** Detailed forest phase classification. More verbose description of forest phase classification than is shown in Table 2 the main manuscript, taking into account differences for evaluation of the phases between VDM types.

| **Phase** | **Variables involved/ dominant** | **Description of mortality dynamics in concert with auxiliary variables** |
| --- | --- | --- |
| Open canopy phase - presence of grasses | %C_veg_, nstems (n ha^-1^), CA (m^2^ ha^-1^), cmort_rate_ (% year^-1^) | Caused by delayed onset in woody establishment; grasses dominate the percentage of total vegetation carbon during this period. This co-occurs with initially low woody mortality rates in models where no stems in the lower size-classes are present, or high mortality rates (if there is competition with grasses for a resource) in models combined with a lack of transition of small stem size-classes into larger ones, where small size-classes continue to lose out over grasses, unless this competitive relationship is not explicit in the model. There is a marked delay in seedling crown area increase, which should not go above 10000 m^2^ ha^-1^. |
| Open canopy phase - growth | nstems (n ha^-1^), %C_veg_, cmortrate (% year^-1^), CA (m^2^ ha^-1^) | Seedlings establish quickly, and grass may be present at the same time for a few years (often shown as grasses making up a significant, but not dominant percentage C in C_veg_ after disturbance). The saplings increase in biomass until the canopy closes and they move into the self-thinning phase. The initial number of seedlings after disturbance is such that growth occurs without a lot of mortality initially (i.e. transition from one size-class into another can be observed in the first years, accompanied with only a small to no reduction in number of stems caused by mortality), and gaps are initially slowly closing until the next phase when self-thinning commences and total stem number decreases. Crown area should be lower or around 10000 m^2^ ha^-1^, and increase quickly.  For models without inter-PFT-competition, note that grasses may be present and form a larger %C_veg_ initially, but there are no mechanisms of suppression by woody PFTs. Instead grasses just shrink their total %C_veg_ as the woody PFTs increase in biomass. |
| Closed canopy- self-thinning | cmort_rate_ (% year^-1^), nstems (n ha^-1^),   CA (m^2^ ha^-1^), %C_veg_, CAI (-) | A mortality spike and steep decline, where not all established individuals grow into a larger size-class, but self-thinning pressure occurs, also hinted at by canopy closure.  The self-thinning phase is marked by transition of small size-classes into larger size-classes, with a substantial decrease in total nstems. Initial CA should be only a bit lower or around 10000 m^2^ ha^-1^, or increase very quickly.  The grass %C_veg_ is negligible.  For a multi-story PPA approach, Crown projection area can go >10000 m^2^ ha^-1^ since a multi-story forest emerges, and new saplings are allowed to emerge and form lower canopy layers. Crown area index is used to indicate canopy closure.  This means that the number of seedlings (total nstems) can still increase, even during the thinning-period (crown-packing). |
| Closed canopy-late successional phase | %C_veg,_ cmort_rate_ (% year^-1^) | A shift in composition is evident in %C_veg_ of the PFTs present and cmort_rate_  can show a slight directional change in the trajectories, where both the PFT, but also the forest structure now shift into a secondary succession stage, moving towards a dynamic equilibrium. |

***Table S9*** *Forest Recovery Phase Classification. Initial phase and self-thinning timing. The results are from iterations with modelling groups and forest recovery benchmark variables (see Table 2, Supplementary Notes S2). (-) means that for model-specific reasons this is not a relevant comparison to make. For example: for CABLE-POP: Self-thinning has something to do with crowding mortality, which is dependent on number-density and Crown Area, but not directly on light or resource availability. Therefore, the timing of selecting the onset of self-thinning using the selected variables is difficult. Likewise, ORCHIDEE uses the concept of self-thinning as the general mechanism by which to induce mortality, making it not possible to assess a self-thinning period per se.*

| **Model\Site** | **FIN** | **BIA** | **BCI** |
| --- | --- | --- | --- |
| JULES-RED | **Phase 1: open canopy-grass coexistence (5 years).** Evidence: Low mortality rate for trees. Presence of grasses in terms of CA, less pronounced with as % of total C_veg_. | **Phase 1: open canopy-grass coexistence (5 years).** Evidence: Low mortality rate for trees. Presence of grasses in terms of CA, less pronounced with as % of total C_veg_. | **Phase 1: open canopy-grass coexistence (5 years).** Evidence: Low mortality rate for trees. Quickest transition to closed-canopy within 70 years |
| FATES | **Phase 2: open canopy-growth phase (70 years)** Evidence: no grasses present | **Phase 1: open canopy grass coexistence phase (6 years)** | **Phase 2: open canopy grass coexistence phase (5 years)** |
| LPJ-GUESS | **Phase 1: open canopy - grass coexistence (5 years).** Evidence: high wood mortality rate (since in competition with grasses); high %total of grasses. | **Phase 1: open -canopy growth (10 years).** Evidence:  Evidence: start with CA < 10000 m^2^ ha^-1^;. Period with transition of smaller size classes into larger ones, without nstem loss. | Phase 1: **open canopy growth (8 years)**. Evidence: v. short period of low mortality rate, followed by rate spike, when self-thinning ( and a reduction in nstem) commences. |
|  |  |  |  |
| CABLE-POP | **Phase 2: open-canopy growth (ca 20 years).** Evidence: nstems initially increasing, then sharply decreasing. | **Phase 2: open-canopy growth (ca 10 years).** Evidence: nstems initially increasing, then sharply decreasing. | **Phase 2: open-canopy growth (ca 2 years).** Evidence: nstems initially increasing, then sharply decreasing. |
| BiomeE: | **Phase2: open canopy growth** (ca 15 years) | **Phase 2: open canopy-growth** (10 years) | **Phase 2:** **open canopy-growth** (7 years) |
| BiomeEP: | **Phase 1:** **open canopy-grass coexistence** (duration: 1 years) | Phase 1: **open canopy-grass coexistence** (ca 10 years) | **Phase 1:** **open canopy-grass coexistence** (ca 10 years) |
| ORCHIDEE | **Phase 3: closed canopy, self-thinning** | **Phase 3: closed canopy, self-thinning** | **Phase 3: closed canopy, self-thinning** |
| SEIB-DGVM | **Phase 1: open canopy- crass coexistence+ growth (50 years).** Evidence: Delayed onset in woody establishment, due to strong interference with grasses | **Phase2: open canopy-grass coexistence + growth (49 years)**. Evidence: Delayed onset in woody establishment due to strong interference with grasses. | **Phase 2: open canopy-growth (10 years)**. Evidence: low canopy area, increasing fast. |
| EDv3 | **Phase 1: open canopy-grass coexistence** (62 years). Evidence: presence of grasses | **Phase 1: open canopy- grass coexistence** (42 years). Evidence: presence of grasses | **Phase 1: open canopy- grass coexistence** (42 years). Evidence: presence of grasses |

**Table S11** Independent identification of self-thinning phase onset from two methods, self-thinning onset determination and forest phase determination. Self-thinning presents also the timing when according to the forest phase criteria and the modeller’s opinion their model commences self-thinning. This is here displayed against the self-thinning onset year that was derived independently using one of the four self-thinning period methods. The timing is confirmed as TRUE, if the two method’s years are within a 15-year margin of each other.

| **Model** | **Initial recovery phase:** | **Self thinning (year post disturbance)** | **Self-thinning onset**  **(year post disturbance)** | **ST timing confirmed** | **Site** |
| --- | --- | --- | --- | --- | --- |
| JULES-RED | Phase 1: Open canopy grass coexistence | 100 | 101 | TRUE | FIN |
| JULES-RED | Phase 1: Open canopy grass coexistence | 60 | 55 | TRUE | BIA |
| JULES-RED | Phase 1: (Brief!) Open canopy grass coexistence | 55 | 42 | TRUE | BCI |
| FATES | Phase 2: Open canopy growth | 48 | 115 | FALSE | FIN |
| FATES | Phase 1: (Brief) Open canopy- grass coexistence | 20 | 48 | FALSE | BIA |
| FATES | Phase 1: (Brief) Open canopy-grass coexistence | 6 | 18 | TRUE | BCI |
| ORCHIDEE | Phase 3: Closed-canopy, Self-thinning |  |  | (-) | FIN |
| ORCHIDEE | Phase 3: Closed-canopy, Self-thinning |  |  | (-) | BIA |
| ORCHIDEE | Phase 3: Closed-canopy, Self-thinning |  |  | (-) | BCI |
| LPJ-GUESS | Phase 1: Open canopy phase grass coexistence | 35 | 41 | TRUE | FIN |
| LPJ-GUESS | Phase 2: Open canopy growth | 15 | 21 | TRUE | BIA |
| LPJ-GUESS | Phase 2: (Brief) Open canopy growth | 10 | 40 | FALSE | BCI |
| CABLE-POP | Phase 2: Open-canopy growth (ca 20 years) | 20 | 72 | (-) | FIN |
| CABLE-POP | Phase 2: Open-canopy growth (ca 10 years) | 10 | 88 | (-) | BIA |
| CABLE-POP | Phase 2: Open-canopy growth (ca 2 years) | 2 | 55 | (-) | BCI |
| BiomeEP | Phase 1: Open canopy-grass coexistence (duration: 10 years) | 30 | 36 | TRUE | FIN |
| BiomeEP | Phase 1: Open canopy-grass coexistence (duration: 10 years) | 30 | 30 | TRUE | BIA |
| BiomeEP | Phase 1: Open canopy-grass coexistence (duration: 10 years) | 30 | 27 | TRUE | BCI |
| BiomeE | Phase 2: Open canopy-growth (15 years) | 20 | 33 | TRUE | FIN |
| BiomeE | Phase 2: Open canopy-growth (10 years) | 30 | 55 | TRUE | BIA |
| BiomeE | Phase 2: Open canopy-growth (7 years) | 15 | 14 | TRUE | BCI |
| SEIB-DGVM | Phase 1: Open-canopy, grass-coexistence phase  &  Open canopy phase – growth (50 years) | 50 | 50 | TRUE | FIN |
| SEIB-DGVM | Phase 1: Open-canopy, grass-coexistence phase  &  Open canopy phase – growth (40) | 40 | 41 | TRUE | BIA |
| SEIB-DGVM | Phase 1: Open-canopy, grass-coexistence phase  &  Open canopy phase – growth (10) | 10 | 10 | TRUE | BCI |
| EDv3 | Phase 1: Open canopy-grass coexistence (62 years) |  | 43 |  | FIN |
| EDv3 | Phase 1: Open canopy-grass coexistence (42 years) |  | 12 |  | BIA |
| EDv3 | Phase 1: Open canopy-grass coexistence (62 years) |  | 31 |  | BCI |

**Notes S2** Forest phase alignment, additional variables used for evidence for identifying the forest Phases.

- For PDF see file: Notes_S2_forest_phase_alignment_combined.pdf
- Screenshot of content:


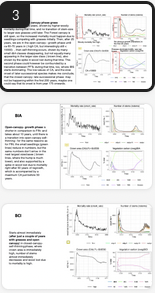

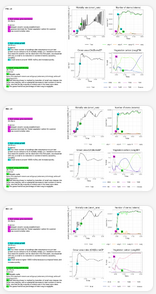


**Table S10** Forest Carbon and dynamics variables summarised across the first 30 years of regrowth and equilibrium. Aboveground woody carbon (Cwood,AG, KgC m-2) after 30 years of disturbance recovery, and at equilibrium, by model and site. WBgrowth fluxes and cmort rates (%) minimum and maximum rates within the first 30 years, and the minimum and maximum across models across these years. No model output marked by -. Min and max columns display the ensemble minimum and maximum value.

| JULES-RED | FATES | ORCHIDEE | LPJ-GUESS | CABLE-POP | BiomeEP | BiomeE-Standalone | SEIB-DGVM | EDv3 | min | max | site | state | variable |
| --- | --- | --- | --- | --- | --- | --- | --- | --- | --- | --- | --- | --- | --- |
| 11.58 | 13.72 | 7.35 | 10.71 | 10.04 | 14.50 | 13.23 | 4.00 | 8.14 | 4.00 | 14.50 | FIN | equilibrium | AGcwood |
| 9.89 | 11.52 | 6.92 | 10.53 | 11.03 | 13.21 | 9.40 | 7.38 | 13.05 | 6.92 | 13.21 | BIA | equilibrium | AGcwood |
| 16.57 | 12.99 | 9.71 | 16.96 | 14.63 | 17.26 | 17.74 | 15.96 | 15.05 | 9.71 | 17.74 | BCI | equilibrium | AGcwood |
| 0.82 | 0.45 | 5.26 | 2.65 | 5.17 | 3.13 | 4.88 | 0.86 | 1.46 | 0.45 | 5.26 | FIN | after 30 years regrowth | AGcwood |
| 4.29 | 4.87 | 5.43 | 3.88 | 6.12 | 5.87 | 3.97 | 1.95 | 5.06 | 1.95 | 6.12 | BIA | after 30 years regrowth | AGcwood |
| 9.60 | 5.35 | 8.36 | 4.94 | 9.34 | 7.66 | 12.84 | 11.02 | 4.99 | 4.94 | 12.84 | BCI | after 30 years regrowth | AGcwood |
| 0.03 | 0.03 | 0.24 | 0.14 | 0.19 | 0.14 | 0.26 | - | 0.09 | 0.03 | 0.26 | FIN | min within 30 first years | WBgrowth |
| 0.19 | 0.39 | 0.35 | 0.31 | 0.25 | 0.27 | 0.18 | - | 0.48 | 0.18 | 0.48 | BIA | min within 30 first years | WBgrowth |
| 0.44 | 0.63 | 0.34 | 0.43 | 0.41 | 0.35 | 0.93 | - | 0.35 | 0.34 | 0.93 | BCI | min within 30 first years | WBgrowth |
| 0.19 | 0.16 | 0.36 | 0.26 | 0.21 | 0.61 | 0.35 | - | 0.19 | 0.16 | 0.61 | FIN | max within 30 first years | WBgrowth |
| 0.41 | 0.54 | 0.55 | 0.42 | 0.26 | 0.80 | 0.23 | - | 0.58 | 0.23 | 0.80 | BIA | max within 30 first years | WBgrowth |
| 1.14 | 0.81 | 0.37 | 0.54 | 0.45 | 0.95 | 1.08 | - | 0.60 | 0.37 | 1.14 | BCI | max within 30 first years | WBgrowth |
| 3.60 | 4.93 | 2.50 | 1.94 | 0.72 | 0.30 | 3.53 | 0.29 | 3.80 | 0.29 | 4.93 | FIN | min within 30 first years | cmort_rate % |
| 3.66 | 6.88 | 5.22 | 4.02 | 1.13 | 0.64 | 2.99 | 0.80 | 5.75 | 0.64 | 6.88 | BIA | min within 30 first years | cmort_rate % |
| 5.00 | 9.48 | 2.66 | 3.61 | 1.86 | 0.59 | 5.71 | 5.77 | 5.17 | 0.59 | 9.48 | BCI | min within 30 first years | cmort_rate % |
| 3.65 | 5.16 | 3.08 | 2.79 | 2.41 | 1.55 | 4.27 | 1.20 | 5.47 | 1.20 | 5.47 | FIN | max within 30 first years | cmort_rate % |
| 4.02 | 28.21 | 8.02 | 5.34 | 2.79 | 2.68 | 3.68 | 1.41 | 7.91 | 1.41 | 28.21 | BIA | max within 30 first years | cmort_rate % |
| 5.21 | 27.24 | 2.98 | 5.78 | 3.73 | 2.29 | 6.72 | 7.03 | 5.64 | 2.29 | 27.24 | BCI | max within 30 first years | cmort_rate % |


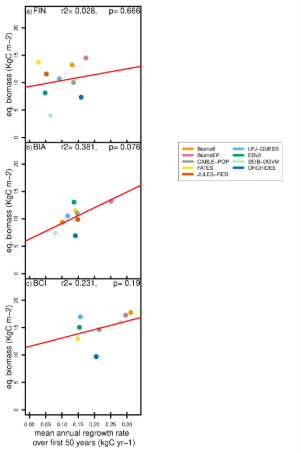


**Fig. S12** Mean equilibrium biomass plotted against mean annual regrowth rate over the first 50 years post-disturbance.The relationship is non-significant for all sites and worst in FIN, where no trend can be discovered. Some clustering is visible at BIA, and, if ORCHIDEE was to be excluded, the relationship may improve for BCI


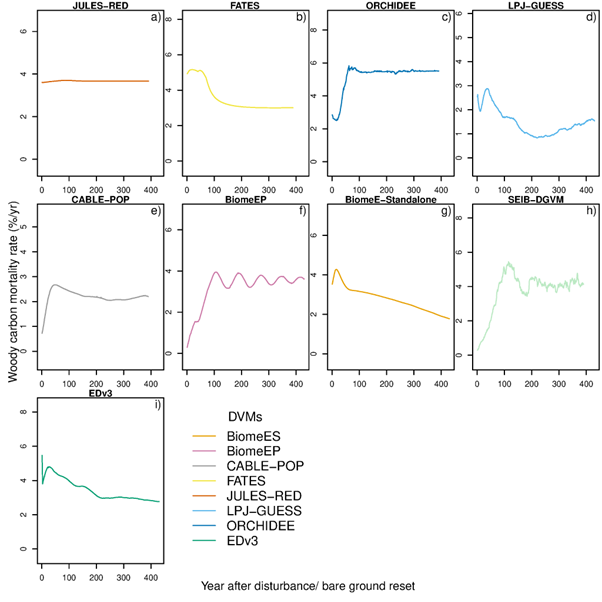


**Fig. S13** Woody mortality rates at FIN, for all models, smoothed using a 30-year running mean.


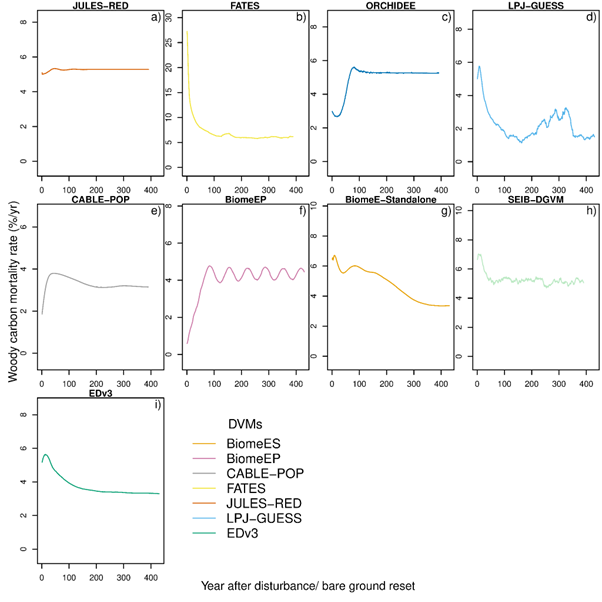


**Fig. S14** Woody mortality rates at BIA, for all models, for all models, smoothed using a 30-year running mean.


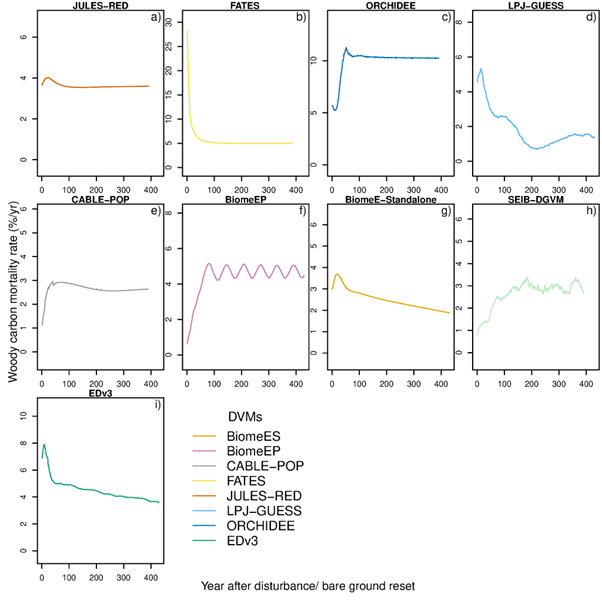


**Fig. S15** Woody mortality rates at BCI, for all models, for all models, smoothed using a 30-year running mean.

**Methods S5** Forest Recovery Phases and Phase Alignment

Forest recovery phase benchmarks were defined with the purpose to especially define the initial forest phase post disturbance. These were first defined by the first author and later iterated with by each modelling team in order to determine what processes are therefore explaining low or high growth or mortality rates in each model’s dynamics going forward.

For example, there seem to be three broad “shapes” emerging from the growth and mortality rates, that can be attributed to the initial phase/ starting point of the recovering forest post disturbance (Figure S16).


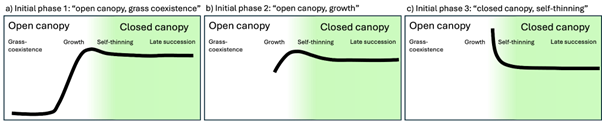


**Fig. S16** Example of a mortality rate trajectory within the forest recovery phases. Representative models for each behaviour can be a) SEIB at FIN, b) LPJ-GUESS at BIA, c) FATES at BCI.

A short description of a mortality trajectory dynamic within the forest recovery phases: **a)** a model starting its trajectory with a low mortality rate is normally the case when sapling density or size is very low, which is normally, but not always occurring with grasses coexisting. When grasses and saplings are allowed to compete for resources, the mortality rate then can also be higher than 0. **b)** a model starting its trajectory with large enough saplings that the presence of grasses is low, but mortality is not yet at its peak, as the canopy, while starting to close (some competition manifests), is not at its peak. Peak mortality, which is caused by even-sized self-thinning, occurs at the time of canopy closure. For PPA-based models this is not necessarily the case, as a second canopy layer can emerge, dampening the overall mortality peak until the canopy has packed more thoroughly, then initiating self-thinning in earnest. To accommodate or this distinction, the colouring of the open-closed canopy transition is shaded. **c)** If many small seedlings are initialised. For example with FATES, the model immediately starts at a self-thinning state (with very small trees), and over time will stabilise into an equilibrium amount of saplings.

**Methods S6** Stand structure benchmarks

LPJ-GUESS stand structure benchmarks

For the stand-structure benchmarks-comparison for LPJ-GUESS, we report “P1” (explained in Notes S1) simulations and explain the reason for this below . “P0” simulations were designed to produce stand-scale outputs conceptually as similar to the observations as possible. In those simulations, the modellers were asked to adjust their model’s settings or to provide model output of a type which allows the most appropriate comparison between model-output and the stand-scale observations.

A structural heterogeneity across patches in LPJ-GUESS, here as patch-mean intended to represent a single forest stand, can only be achieved by activating disturbance mortality, which kills e.g. large trees in the tropics allowing for regeneration and presence of smaller cohorts (see Figure S13,b), where the stem number in the smaller size classes is closer to the observations, in P1 simulations).


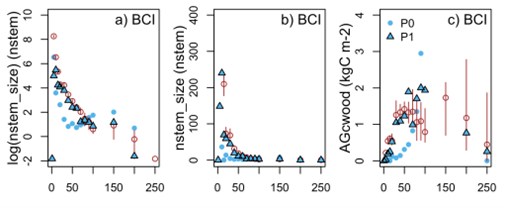


**Fig. S17** Stand structure from LPJ-GUESS run output at simulation year 450 with no patch-destroying disturbance (blue circles, P0), and a 100-year disturbance interval (default) turned on (blue triangles, P1). Observational data ranges are the same as elsewhere and reported in table S6 for stand structure, BCI.


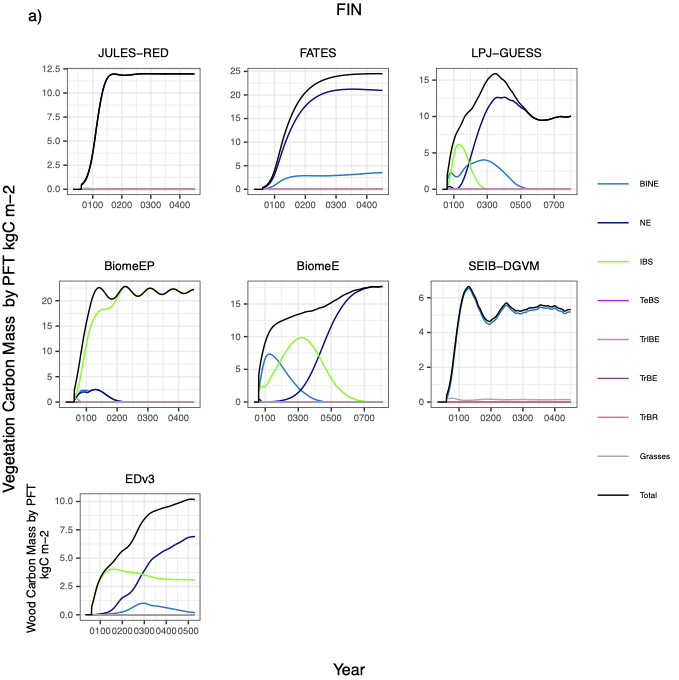


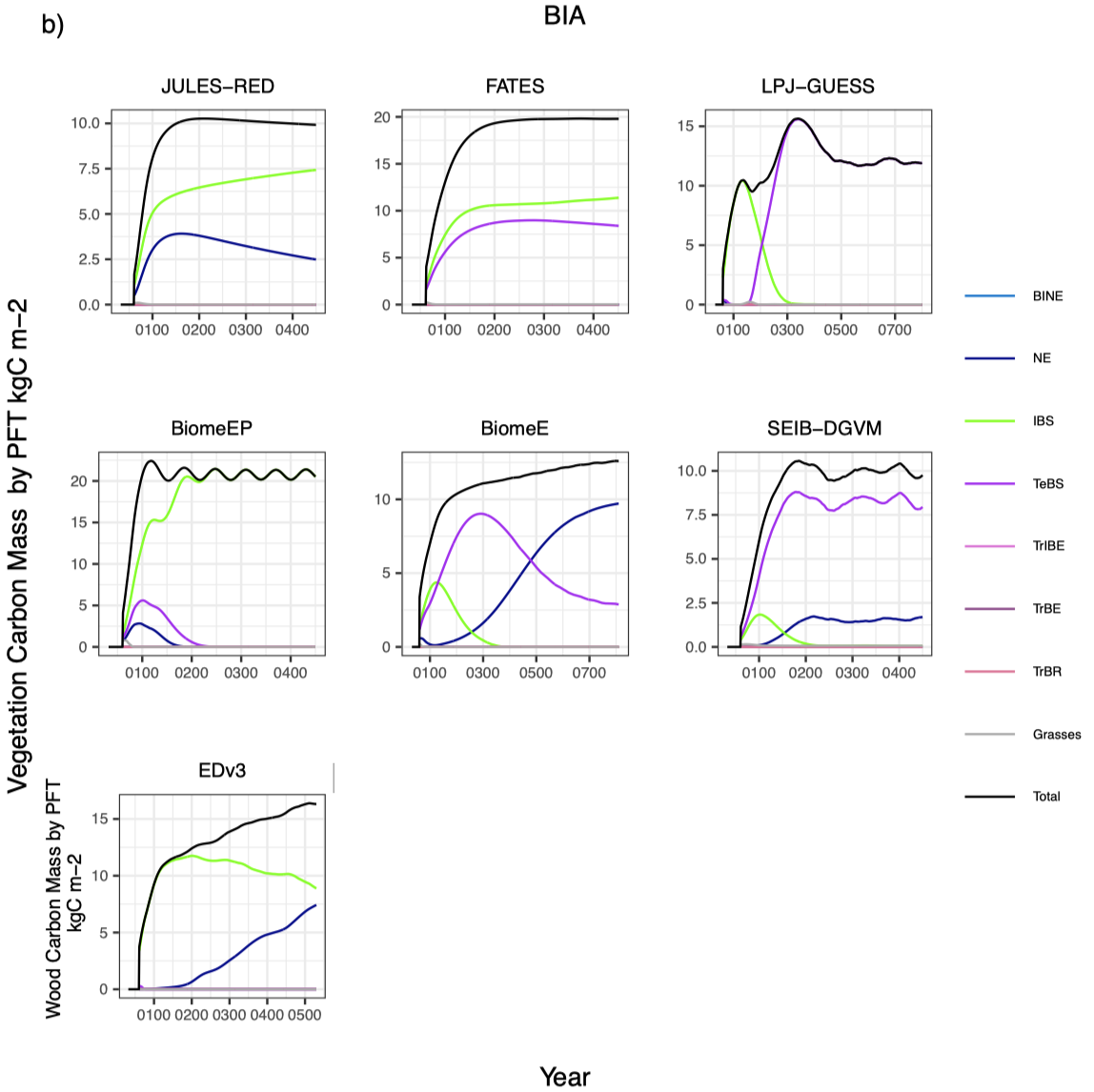


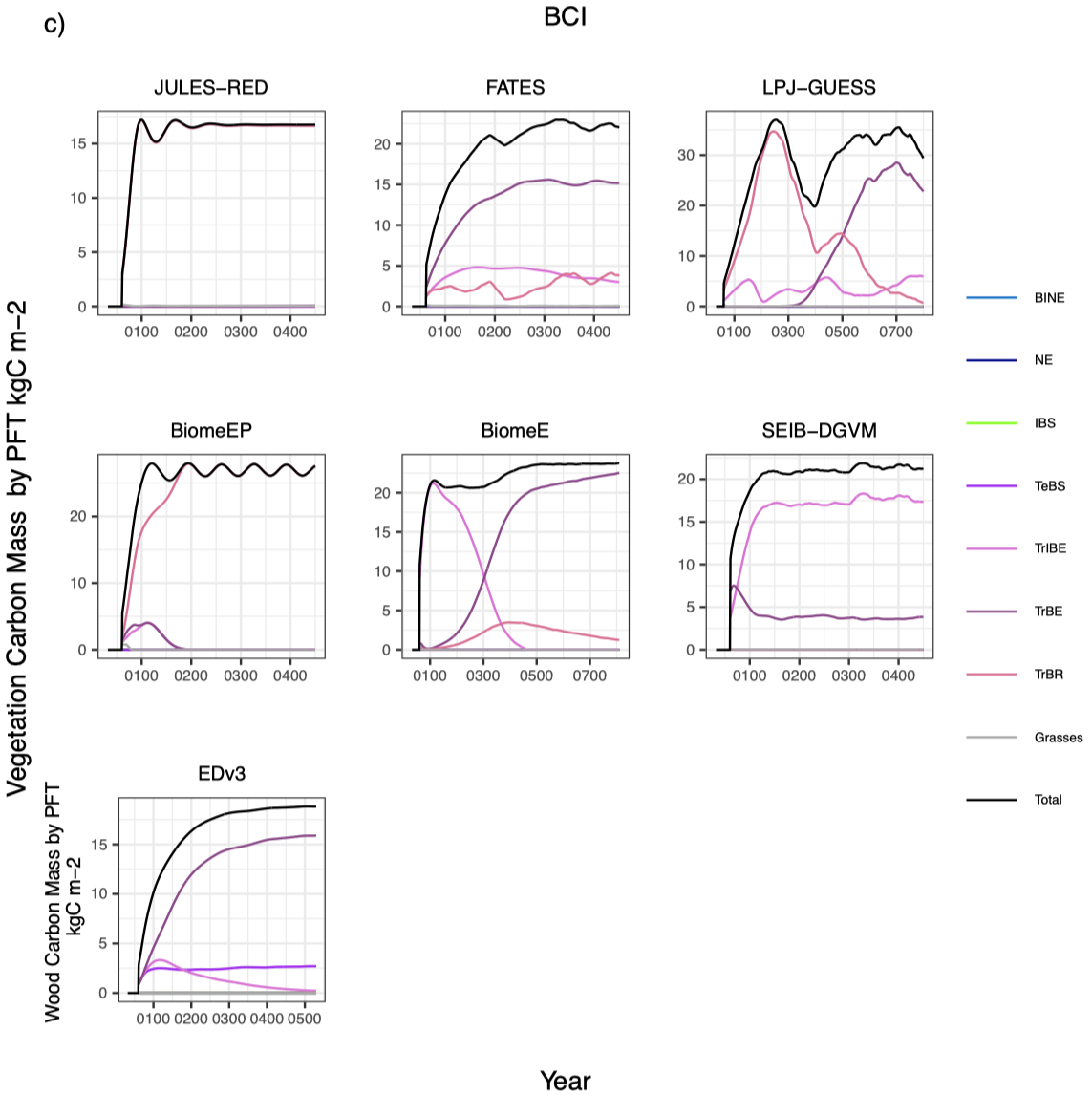


**Fig. S18** Succession patterns in models that simulate between-PFT competition as part of demographic dynamics. The definition and parameterization of PFTs differ between models, with PFT-species mapping provided in Table S1.


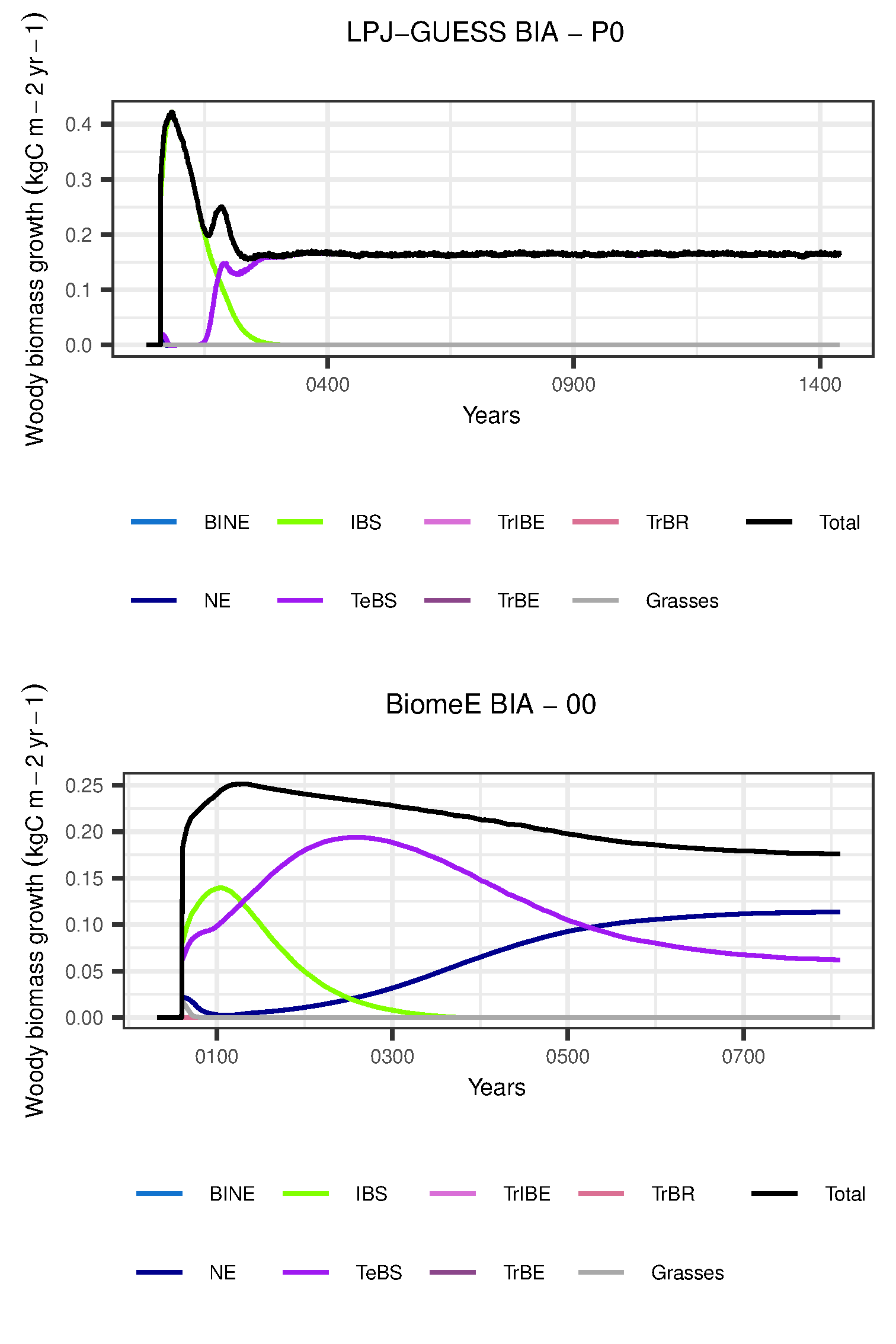


**Fig. S19** Growth rates at Bialowieza for LPJ-GUESS and BiomeE, showcasing the impact of PFT succession on the growth rate during recovery (30-year smoothed).

**Notes S3** Stand structure benchmark per model and site, with the observational data ranges reported in table S6.

- See file NotesS3_Benchmark_stand_structure_per_model.pdf
- Screenshot of content:


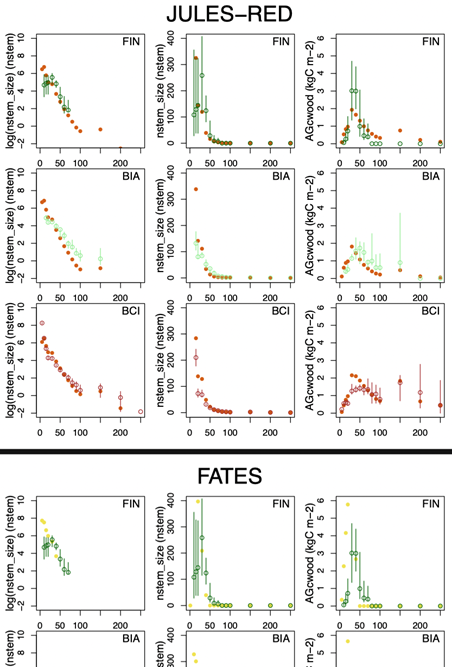


**Methods S7** ”Naïve” – unaligned regrowth rate analysis

This analysis relates to the statement in the manuscript: “Mean 50-year regrowth rate does not seem to be linked to final equilibrium biomass values (Figure S10)”

We use the first 50 years of Cwood,AG and calculate the average recovery rate:

$$Rec_{rate,50}= \frac{\sum_{i=1}^{50} C_{wood,AG}}{50}$$

(eq. S5)

Equilibrium $C_{wood,AG},{C_{wood,AG}}_{equilibrium}$ is calculated as:

${cwood}_{equilibrium}=\frac{\sum_{i=lower}^{upper} C_{wood,AG}}{upper-lower}$ (eq. S6)

Equilibrium time period ranges (lower, upper) can be found in Table S5.

We use a linear regression analysis to obtain p and r2 values for assessment of the ensemble’s prediction of this relationship. For this, we apply the function lm() in R, with default settings and extract the relevant values using the summary() function.

Given the above analysis, there seems to be no link between regrowth rate and equilibrium biomass. We believe that this is mainly caused by the initial establishment conditions of the model post-disturbance. Without alignment of the models along the forest recovery phases, applying a simple mean operation on the first 50 years of carbon during regrowth will lead to highly divergent results, and insignificant relationship between the first years of recovery and the mature forest stage. We expect that this behaviour is mitigated once initial establishment conditions are identified and model trajectories are aligned. For example, ORCHIDEE begins in a very different forest recovery phase — self-thinning — compared to SEIB, which starts with an open canopy and grass presence, which impacts the initial carbon present. The initial conditions of the model have little to do with the mature forest “equilibrium values” of biomass, but an investigation was beyond the scope of this study.

**Notes S4** Regrowth benchmark per model and site, with the observational data ranges reported in table S6.

- See file NotesS4_Benchmark_regrowth_per_model.pdf
- Screenshot of content:


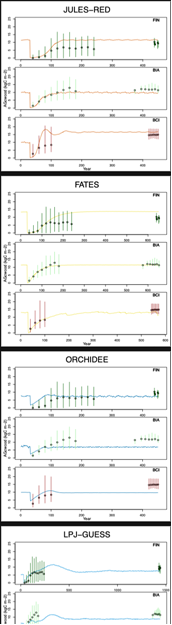

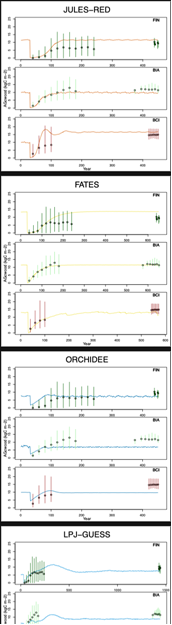


**Methods S8** Mean temperature analysis for regrowth sites

To analyse whether the growth temperatures driving the regrowth dynamics of the three sites FIN, BIA and BCI are representative of the regrowth dynamics that make up the benchmarking data, we analyse CRU JRA v2.2 forcing data. We extract all coordinates from the regrowth sites in the Tropics and Temperate regions that make up the regrowth benchmark dataset. For FIN, exact site locations were not available, and we randomly sampled 100 lat-lon combinations from central and southern Finland. We present a density plot (frequency distribution) of the full century mean annual temperatures at all regrowth sites (black), and the frequency distribution of the 30 year forcing data used for FIN, BIA and BCI (Fig. S20). Frequency distributions, and therefore assumed behaviour of the regrowth dynamics, are within the most frequent temperature ranges for all sites, except for FIN, where the last 30 years seem a bit warmer. One can interpret this as simulated FIN regrowth dynamics probably being above the median dynamics of the benchmarking data, whereas the other two sites might be behaving in their regrowth dynamics relatively similar to the sample median.


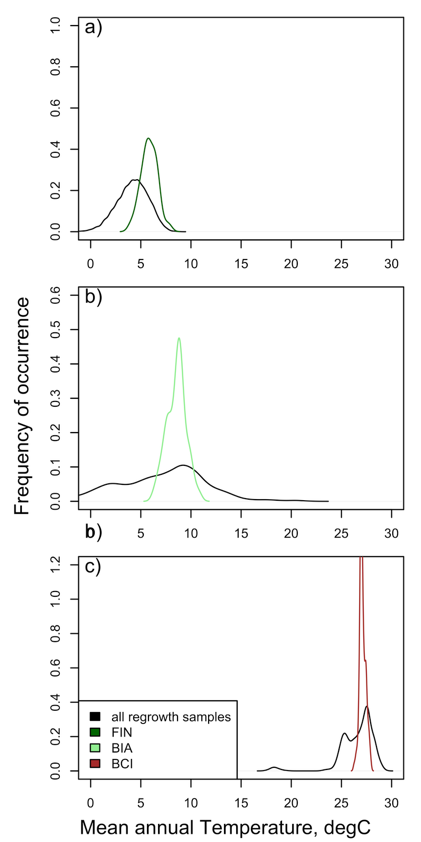


**Fig. S20** Frequency distribution of mean temperature for the years 1900-2023 for all sites for which regrowth datapoints exist (black), and frequency distribution of mean temperature for years 1991-2020, for the three simulation sites.

**Bibliography:**

**Argles APK, Moore JR, Huntingford C, Wiltshire AJ, Harper AB, Jones CD, Cox PM**. **2020**. Robust Ecosystem Demography (RED version 1.0): a parsimonious approach to modelling vegetation dynamics in Earth system models. *Geoscientific Model Development* **13**: 4067–4089.

**Atkin O, Abdul Bahar N, Bloomfield K, Griffin KL, Heskel MA, Huntingford C, Martinez-de la Torre A, Turnbull MH**. **2017**. Leaf Respiration in Terrestrial Biosphere Models. In: null GT and JG, ed. Plant Respiration: Metabolic Fluxes and Carbon Balance. Switzerland: Springer International Publishing AG, 107–142.

**Boucher O, Servonnat J, Albright AL, Aumont O, Balkanski Y, Bastrikov V, Bekki S, Bonnet R, Bony S, Bopp L, *et al.*** **2020**. Presentation and Evaluation of the IPSL-CM6A-LR Climate Model. *Journal of Advances in Modeling Earth Systems* **12**: e2019MS002010.

**Brzeziecki B, Pommerening A, Miścicki S, Drozdowski S, Żybura H**. **2016**. A common lack of demographic equilibrium among tree species in Białowieża National Park (NE Poland): evidence from long-term plots (B Collins, Ed.). *Journal of Vegetation Science* **27**: 460–469.

**Cannell MGR**. **1982**. *World forest biomass and primary production data*. London: Academic Press.

**Clark DB, Mercado LM, Sitch S, Jones CD, Gedney N, Best MJ, Pryor M, Rooney GG, Essery RLH, Blyth E, *et al.*** **2011**. The Joint UK Land Environment Simulator (JULES), model description – Part 2: Carbon fluxes and vegetation dynamics. *Geoscientific Model Development* **4**: 701–722.

**Condit R, Pérez R, Aguilar S, Lao S, Foster R, Hubbell S**. **2019**. Complete data from the Barro Colorado 50-ha plot: 423617 trees, 35 years. : 573292682 bytes.

**Cushman KC, Bunyavejchewin S, Cárdenas D, Condit R, Davies SJ, Duque Á, Hubbell SP, Kiratiprayoon S, Lum SKY, Muller-Landau HC**. **2021**. Variation in trunk taper of buttressed trees within and among five lowland tropical forests. *Biotropica* **53**: 1442–1453.

**Davies SJ, Abiem I, Abu Salim K, Aguilar S, Allen D, Alonso A, Anderson-Teixeira K, Andrade A, Arellano G, Ashton PS, *et al.*** **2021**. ForestGEO: Understanding forest diversity and dynamics through a global observatory network. *Biological Conservation* **253**: 108907.

**Delitti WBC, Meguro M, Pausas JG**. **2006**. Biomass and mineralmass estimates in a ‘cerrado’ ecosystem. *Revista Brasileira de Botânica* **29**: 531–540.

**Fisher RA, Muszala S, Verteinstein M, Lawrence P, Xu C, McDowell NG, Knox RG, Koven C, Holm J, Rogers BM, *et al.*** **2015**. Taking off the training wheels: the properties of a dynamic vegetation model without climate envelopes, CLM4.5(ED). *Geoscientific Model Development* **8**: 3593–3619.

**ForestPlots.net, Blundo C, Carilla J, Grau R, Malizia A, Malizia L, Osinaga-Acosta O, Bird M, Bradford M, Catchpole D, *et al.*** **2021**. Taking the pulse of Earth’s tropical forests using networks of highly distributed plots. *Biological Conservation* **260**: 108849.

**Forrester DI, Tachauer IHH, Annighoefer P, Barbeito I, Pretzsch H, Ruiz-Peinado R, Stark H, Vacchiano G, Zlatanov T, Chakraborty T, *et al.*** **2017**. Generalized biomass and leaf area allometric equations for European tree species incorporating stand structure, tree age and climate. *Forest Ecology and Management* **396**: 160–175.

**Haverd V, Smith B, Cook GD, Briggs PR, Nieradzik L, Roxburgh SH, Liedloff A, Meyer CP, Canadell JG**. **2013**. A stand-alone tree demography and landscape structure module for Earth system models. *Geophysical Research Letters* **40**: 5234–5239.

**Haverd V, Smith B, Nieradzik L, Briggs P**. **2014**. A stand-alone tree demography and landscape structure module for Earth system models: Integration with inventory data from temperate and boreal forests. *Biogeosciences* **11**: 4039–4055.

**Haverd V, Smith B, Nieradzik L, Briggs PR, Woodgate W, Trudinger CM, Canadell JG, Cuntz M**. **2018**. A new version of the CABLE land surface model (Subversion revision r4601) incorporating land use and land cover change, woody vegetation demography, and a novel optimisation-based approach to plant coordination of photosynthesis. *Geoscientific Model Development* **11**: 2995–3026.

**Hurtt GC, Moorcroft PauLR, And SWP, Levin SA**. **1998**. Terrestrial models and global change: challenges for the future. *Global Change Biology* **4**: 581–590.

**Hurtt GC, Pacala SW, Moorcroft PR, Caspersen J, Shevliakova E, Houghton RA, Moore B**. **2002**. Projecting the future of the U.S. carbon sink. *Proceedings of the National Academy of Sciences of the United States of America* **99**: 1389–1394.

**Kohyama TS, Kohyama TI, Sheil D**. **2018**. Definition and estimation of vital rates from repeated censuses: Choices, comparisons and bias corrections focusing on trees. *Methods in Ecology and Evolution* **9**: 809–821.

**Kohyama TS, Kohyama TI, Sheil D**. **2019**. Estimating net biomass production and loss from repeated measurements of trees in forests and woodlands: Formulae, biases and recommendations. *Forest Ecology and Management* **433**: 729–740.

**Korhonen K, Ahola A, Heikkinen J, Henttonen H, Hotanen J-P, Ihalainen A, Melin M, Pitkänen J, Räty M, Sirviö M, *et al.*** **2021**. Forests of Finland 2014–2018 and their development 1921–2018. *Silva Fennica* **55**.

**Koven CD, Knox RG, Fisher RA, Chambers JQ, Christoffersen BO, Davies SJ, Detto M, Dietze MC, Faybishenko B, Holm J, *et al.*** **2020**. Benchmarking and parameter sensitivity of physiological and vegetation dynamics using the Functionally Assembled Terrestrial Ecosystem Simulator (FATES) at Barro Colorado Island, Panama. *Biogeosciences* **17**: 3017–3044.

**Krinner G, Viovy N, Noblet‐Ducoudré N de, Ogée J, Polcher J, Friedlingstein P, Ciais P, Sitch S, Prentice IC**. **2005**. A dynamic global vegetation model for studies of the coupled atmosphere-biosphere system. *Global Biogeochemical Cycles* **19**.

**Lawrence DM, Fisher RA, Koven CD, Oleson KW, Swenson SC, Bonan G, Collier N, Ghimire B, Kampenhout L van, Kennedy D, *et al.*** **2019**. CLM5.0 Technical Notes.

**Lehtonen A, Mäkipää R, Heikkinen J, Sievänen R, Liski J**. **2004**. Biomass expansion factors (BEFs) for Scots pine, Norway spruce and birch according to stand age for boreal forests. *Forest Ecology and Management* **188**: 211–224.

**Liaw A, Wiener M**. **2002**. Classiﬁcation and Regression by randomForest. *R News* **2**: 18–22.

**Lindeskog M, Smith B, Lagergren F, Sycheva E, Ficko A, Pretzsch H, Rammig A**. **2021**. Accounting for forest management in the estimation of forest carbon balance using the dynamic vegetation model LPJ-GUESS (v4.0, r9710): implementation and evaluation of simulations for Europe. *Geoscientific Model Development* **14**: 6071–6112.

**Ma L, Hurtt G, Ott L, Sahajpal R, Fisk J, Lamb R, Tang H, Flanagan S, Chini L, Chatterjee A, *et al.*** **2022**. Global evaluation of the Ecosystem Demography model (ED v3.0). *Geoscientific Model Development* **15**: 1971–1994.

**Moorcroft PR, Hurtt GC, Pacala SW**. **2001**. A Method for Scaling Vegetation Dynamics: The Ecosystem Demography Model (ed). *Ecological Monographs* **71**: 557–586.

**Moore JR, Argles APK, Zhu K, Huntingford C, Cox PM**. **2020**. Validation of demographic equilibrium theory against tree-size distributions and biomass density in Amazonia. *Biogeosciences* **17**: 1013–1032.

**Moore JR, Zhu K, Huntingford C, Cox PM**. **2018**. Equilibrium forest demography explains the distribution of tree sizes across North America. *Environmental Research Letters* **13**: 084019.

**Naudts K, Chen Y, McGrath MJ, Ryder J, Valade A, Otto J, Luyssaert S**. **2016**. Europe’s forest management did not mitigate climate warming. *Science* **351**: 597–600.

**Oleson K, Lawrence M, Bonan B, Drewniak B, Huang M, Koven D, Levis S, Li F, Riley J, Subin M, *et al.*** **2013**. Technical description of version 4.5 of the Community Land Model (CLM).

**Peltoniemi M, Mäkipää R**. **2011**. Quantifying distance-independent tree competition for predicting Norway spruce mortality in unmanaged forests. *Forest Ecology and Management* **261**: 30–42.

**Piao S, Ciais P, Friedlingstein P, de Noblet-Ducoudré N, Cadule P, Viovy N, Wang T**. **2009**. Spatiotemporal patterns of terrestrial carbon cycle during the 20th century. *Global Biogeochemical Cycles* **23**.

**Poorter L, Bongers F, Aide TM, Almeyda Zambrano AM, Balvanera P, Becknell JM, Boukili V, Brancalion PHS, Broadbent EN, Chazdon RL, *et al.*** **2016**. Biomass resilience of Neotropical secondary forests. *Nature* **530**: 211–214.

**Powell DC**. **2000**. *Potential vegetation, disturbance, plant succession, and other aspects of forest ecology*. Pacific Northwest Region: USA Forest Service.

**Prentice IC, Dong N, Gleason SM, Maire V, Wright IJ**. **2014**. Balancing the costs of carbon gain and water transport: testing a new theoretical framework for plant functional ecology. *Ecology Letters* **17**: 82–91.

**Pugh TAM, Arneth A, Kautz M, Poulter B, Smith B**. **2019**. Important role of forest disturbances in the global biomass turnover and carbon sinks. *Nature Geoscience* **12**: 730–735.

**Purves DW, Lichstein JW, Strigul N, Pacala SW**. **2008**. Predicting and understanding forest dynamics using a simple tractable model. *Proceedings of the National Academy of Sciences* **105**: 17018–17022.

**R Core Team**. **2023**. R: A language and environment for statistical computing. R.

**Rabin SS, Melton JR, Lasslop G, Bachelet D, Forrest M, Hantson S, Kaplan JO, Li F, Mangeon S, Ward DS, *et al.*** **2017**. The Fire Modeling Intercomparison Project (FireMIP), phase 1: experimental and analytical protocols with detailed model descriptions. *Geoscientific Model Development* **10**: 1175–1197.

**Réjou-Méchain M, Tanguy A, Piponiot C, Chave J, Hérault B**. **2017**. : an r package for estimating above-ground biomass and its uncertainty in tropical forests. *Methods in Ecology and Evolution* **8**: 1163–1167.

**Repo A, Rajala T, Henttonen HM, Lehtonen A, Peltoniemi M, Heikkinen J**. **2021**. Age-dependence of stand biomass in managed boreal forests based on the Finnish National Forest Inventory data. *Forest Ecology and Management* **498**: 119507.

**Repola J**. **2008**. Biomass equations for birch in Finland. *Silva Fennica* **42**.

**Repola J**. **2009**. Biomass equations for Scots pine and Norway spruce in Finland. *Silva Fennica* **43**.

**Ricciuto D, Sargsyan K, Thornton P**. **2017**. The Impact of Parametric Uncertainties on Biogeochemistry in the E3SM Land Model. *Journal of Advances in Modeling Earth Systems* **10**: 297–319.

**Sato H, Itoh A, Kohyama T**. **2007**. SEIB–DGVM: A new Dynamic Global Vegetation Model using a spatially explicit individual-based approach. *Ecological Modelling* **200**: 279–307.

**Sato H, Shibuya M, Hiura T**. **2023**. Reconstructing spatiotemporal dynamics of mixed conifer and broad-leaved forests with a spatially explicit individual-based dynamic vegetation model. *Ecological Research* **38**: 465–478.

**Smith B, Prentice IC, Sykes MT**. **2001**. Representation of vegetation dynamics in the modelling of terrestrial ecosystems: comparing two contrasting approaches within European climate space. *Global Ecology*: 25.

**Smith B, Wårlind D, Arneth A, Hickler T, Leadley P, Siltberg J, Zaehle S**. **2014**. Implications of incorporating N cycling and N limitations on primary production in an individual-based dynamic vegetation model. *Biogeosciences* **11**: 2027–2054.

**Stocker BD, Wang H, Smith NG, Harrison SP, Keenan TF, Sandoval D, Davis T, Prentice IC**. **2020**. P-model v1.0: an optimality-based light use efficiency model for simulating ecosystem gross primary production. *Geoscientific Model Development* **13**: 1545–1581.

**Strigul N, Pristinski D, Purves D, Dushoff J, Pacala S**. **2008**. Scaling from Trees to Forests: Tractable Macroscopic Equations for Forest Dynamics. *Ecological Monographs* **78**: 523–545.

**Teobaldelli M, Somogyi Z, Migliavacca M, Usoltsev VA**. **2009**. Generalized functions of biomass expansion factors for conifers and broadleaved by stand age, growing stock and site index. *Forest Ecology and Management* **257**: 1004–1013.

**Tjiputra JF, Roelandt C, Bentsen M, Lawrence DM, Lorentzen T, Schwinger J, Seland Ø, Heinze C**. **2013**. Evaluation of the carbon cycle components in the Norwegian Earth System Model (NorESM). *Geoscientific Model Development* **6**: 301–325.

**Usoltsev VA**. **2001**. *Forest biomass of Northern Eurasia: database and geography.* Yekaterinburg: Ural Branch of Russian Academy of Sciences.

**Vuichard N, Messina P, Luyssaert S, Guenet B, Zaehle S, Ghattas J, Bastrikov V, Peylin P**. **2019**. Accounting for carbon and nitrogen interactions in the global terrestrial ecosystem model ORCHIDEE (trunk version, rev 4999): multi-scale evaluation of gross primary production. *Geoscientific Model Development* **12**: 4751–4779.

**Wang H, Prentice IC, Keenan TF, Davis TW, Wright IJ, Cornwell WK, Evans BJ, Peng C**. **2017**. Towards a universal model for carbon dioxide uptake by plants. *Nature Plants* **3**: 734–741.

**Weng E, Dybzinski R, Farrior CE, Pacala SW**. **2019**. Competition alters predicted forest carbon cycle responses to nitrogen availability and elevated CO_2_: simulations using an explicitly competitive, game-theoretic vegetation demographic model. *Biogeosciences* **16**: 4577–4599.

**Weng E, Farrior CE, Dybzinski R, Pacala SW**. **2017**. Predicting vegetation type through physiological and environmental interactions with leaf traits: evergreen and deciduous forests in an earth system modeling framework. *Global Change Biology* **23**: 2482–2498.

**Weng ES, Malyshev S, Lichstein JW, Farrior CE, Dybzinski R, Zhang T, Shevliakova E, Pacala SW**. **2015**. Scaling from individual trees to forests in an Earth system modeling framework using a mathematically tractable model of height-structured competition. *Biogeosciences* **12**: 2655–2694.

**Włoczewski, T. 1954**. Materiały do badania zale_znosci miedzy z drzewostanem i gleba w przestrzeni i w czasie. Prace IBL 123: z 161–249.
